# Supplementary material for: omicsGMF: a multi-tool for dimensionality reduction, batch correction and imputation in bulk- and single-cell proteomics
Source: Nat Commun. 2026 May 20;17:6650. doi: 10.1038/s41467-026-73402-8 (PMC13381881; doi:10.1038/s41467-026-73402-8)
Supplement: Supplementary file 1 — Supplementary Information [file 41467_2026_73402_MOESM1_ESM.pdf]

omicsGMF: a multi-tool for dimensionality reduction, batch correction and imputation in bulk- and single-cell proteomics.

Alexandre Segers[1,2,3], Cristian Castiglione[4†], Christophe Vanderaa[1,2,5†], Lennart Martens[2,5,6,7], Davide Risso[8*], Lieven Clement[1,9*]

[1]Department of Mathematics, Computer Science and Statistics, Ghent University, Ghent, Belgium.
[2]Department of Biomolecular Medicine, Ghent University, Ghent, Belgium.
[3]Center for Medical Genetics, Ghent University Hospital, Ghent, Belgium.
[4]Bocconi Institute for Data Science and Analytics, Bocconi University, Milan, Italy.
[5]VIB-UGent Center for Medical Biotechnology, VIB, Ghent, Belgium.
[6]BioOrganic Mass Spectrometry Laboratory (LSMBO), IPHC UMR 7178, University of Strasbourg, CNRS, Strasbourg, France.
[7]Infrastructure Nationale de Protéomique, ProFI-UAR 2048, Strasbourg, France.
[8]Department of Statistical Sciences, University of Padova, Padova, Italy.
[9]Bioinformatics Institute Ghent, Ghent University, Ghent, Belgium.

*Corresponding authors (contributed equally): lieven.clement@ugent.be; davide.risso@unipd.it
†These authors contributed equally to this work.

Supplementary Figures

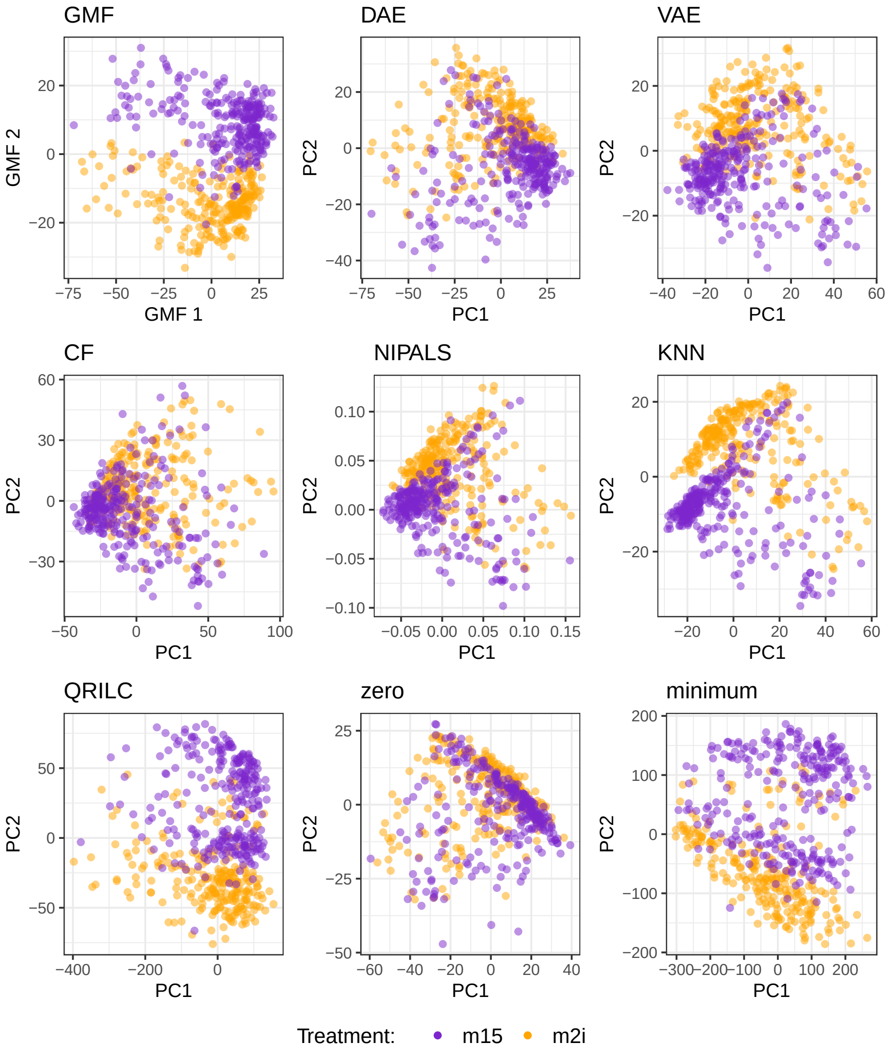

Supp. Fig. 1 Low-dimensional visualization of the Petrosius data [1], colored by inhibitor treatment. omicsGMF and NIPALS directly estimate latent factors that have a similar interpretation as regular PCA. The other visualizations are obtained by imputation of missing values using DAE, VAE, CF, KNN, QRILC, zero and minimum, followed by PCA. Source data are provided as a Source Data file.

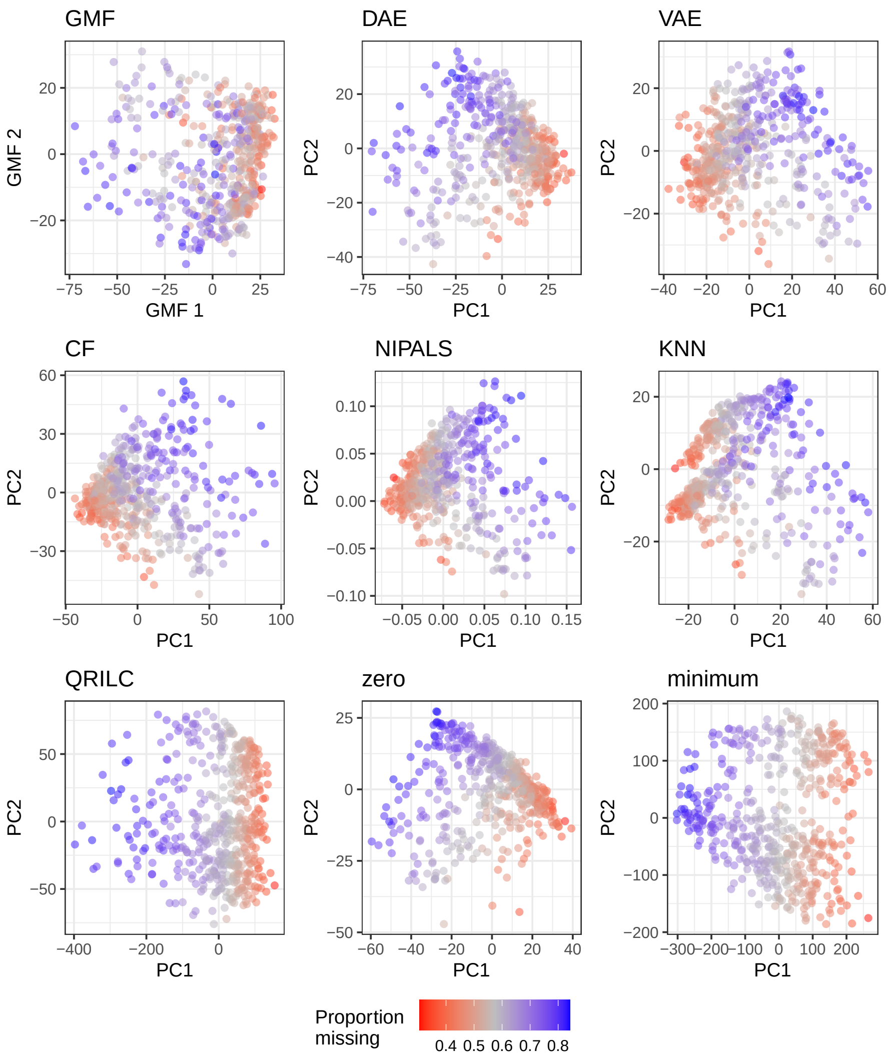

Supp. Fig. 2 Low-dimensional visualization of the Petrosius data [1], colored by proportion of missing values in the cell. omicsGMF and NIPALS directly estimate latent factors that have a similar interpretation as regular PCA. The other visualizations are obtained by imputation of missing values using DAE, VAE, CF, KNN, QRILC, zero and minimum, followed by PCA. Source data are provided as a Source Data file.

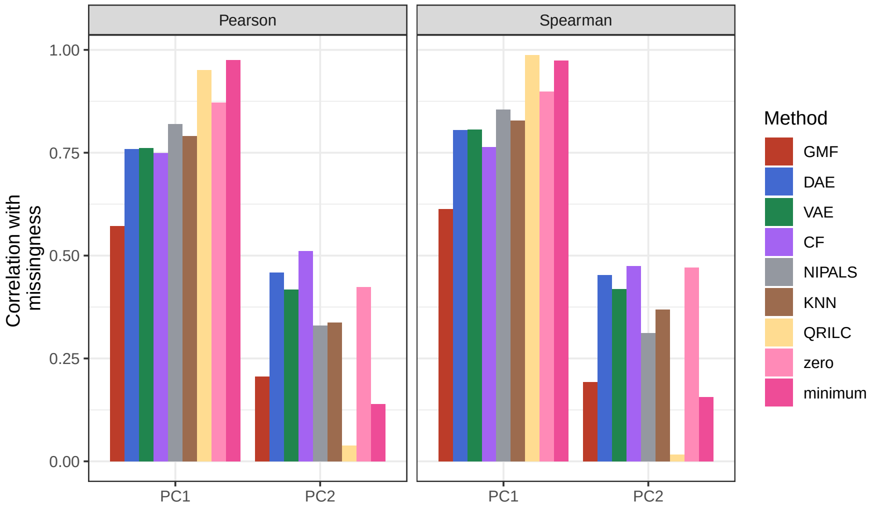

Supp. Fig. 3 Barplots of the Pearson (left) and Spearman (right) correlations between the first two principal components and the proportion of missing values in a cell from the Petrosius dataset [1]. Source data are provided as a Source Data file.

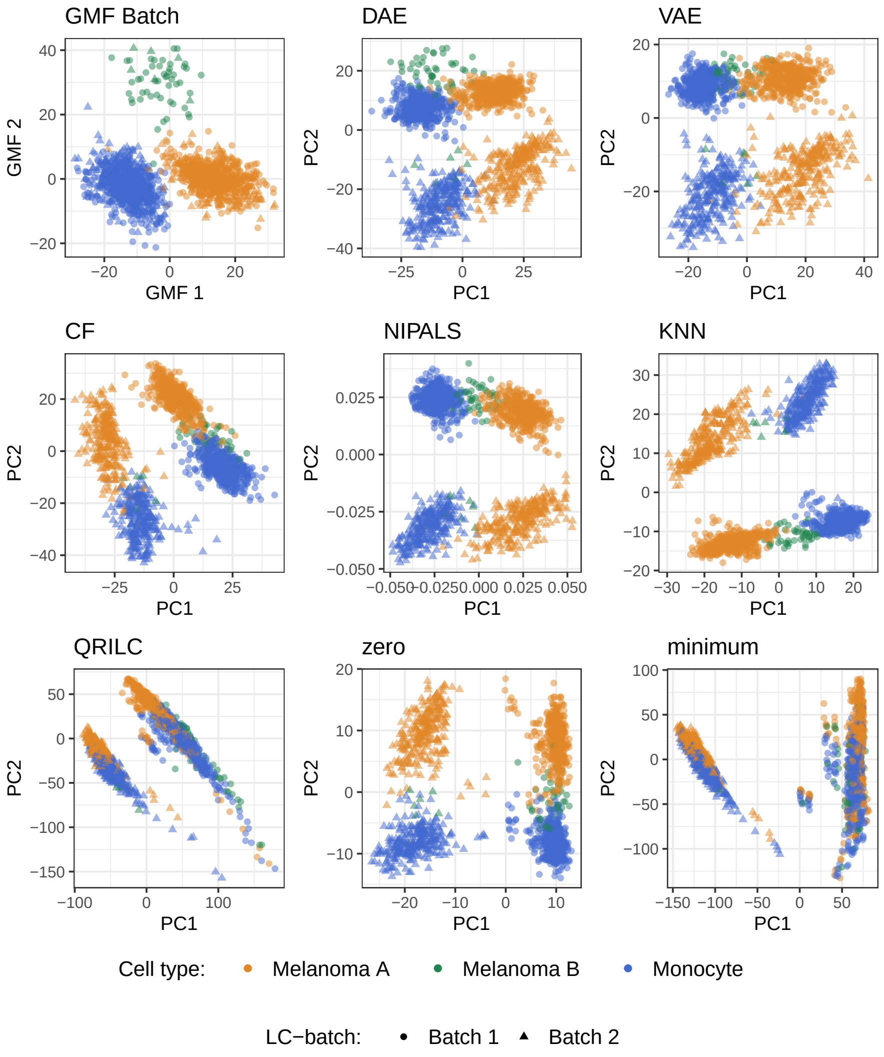

Supp. Fig. 4 Low-dimensional visualization of the Leduc data [2], colored by cell type. omicsGMF and NIPALS directly estimate latent factors that have a similar interpretation as regular PCA. Here, omicsGMF also simultaneously accounts for known batch effects, resulting in a better representation of the biological signal. The other visualizations are obtained by imputation of missing values using DAE, VAE, CF, KNN, QRILC, zero and minimum prior to PCA. Source data are provided as a Source Data file.

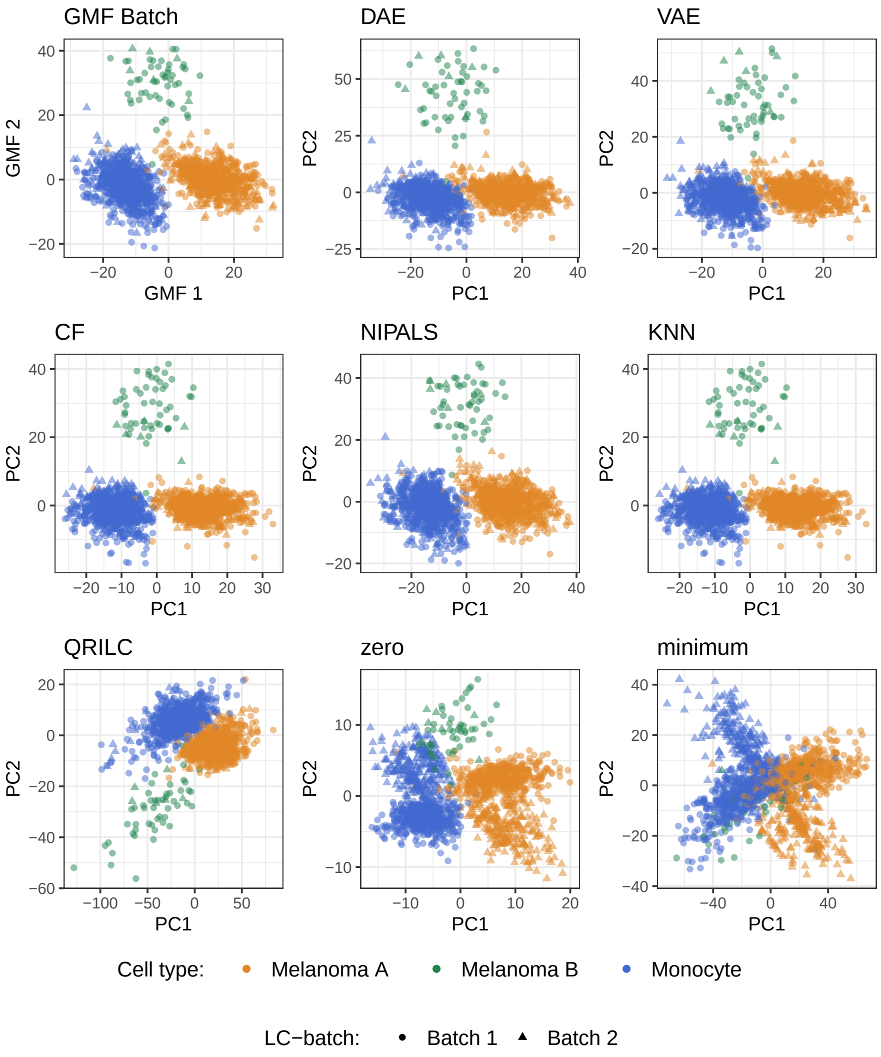

Supp. Fig. 5 Low-dimensional visualization of the Leduc data [2] after batch-correction, colored by cell type. omicsGMF directly accounts for known batch effects. The other visualizations are obtained by imputation of missing values using DAE, VAE, CF, NIPALS, KNN, QRILC, zero and minimum, followed by batch-correction using linear regression and PCA on the remaining residuals. Source data are provided as a Source Data file.

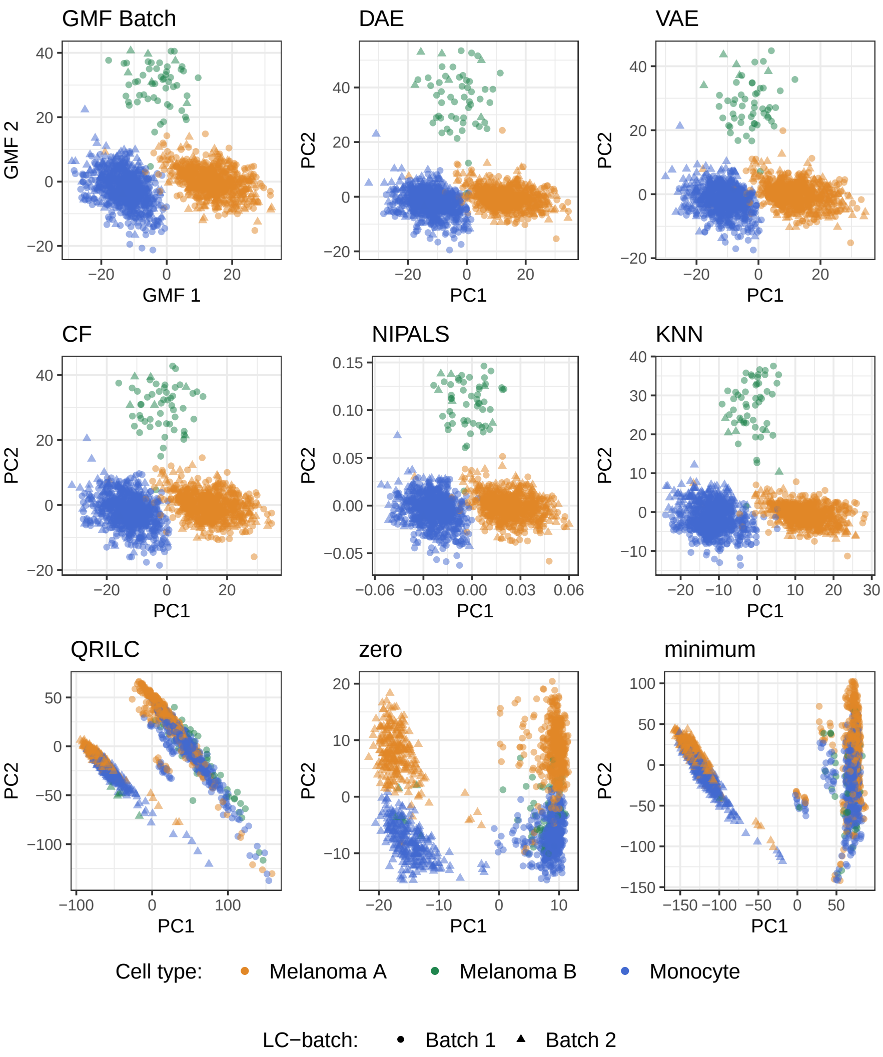

Supp. Fig. 6 Low-dimensional visualization of the Leduc data [2] after batch-correction, colored by cell type. omicsGMF directly accounts for known batch effects. The other visualizations are obtained by sequentially performing batch-correction using linear regression, imputation of missing values using DAE, VAE, CF, NIPALS, KNN, QRILC, and minimum prior to PCA. Source data are provided as a Source Data file.

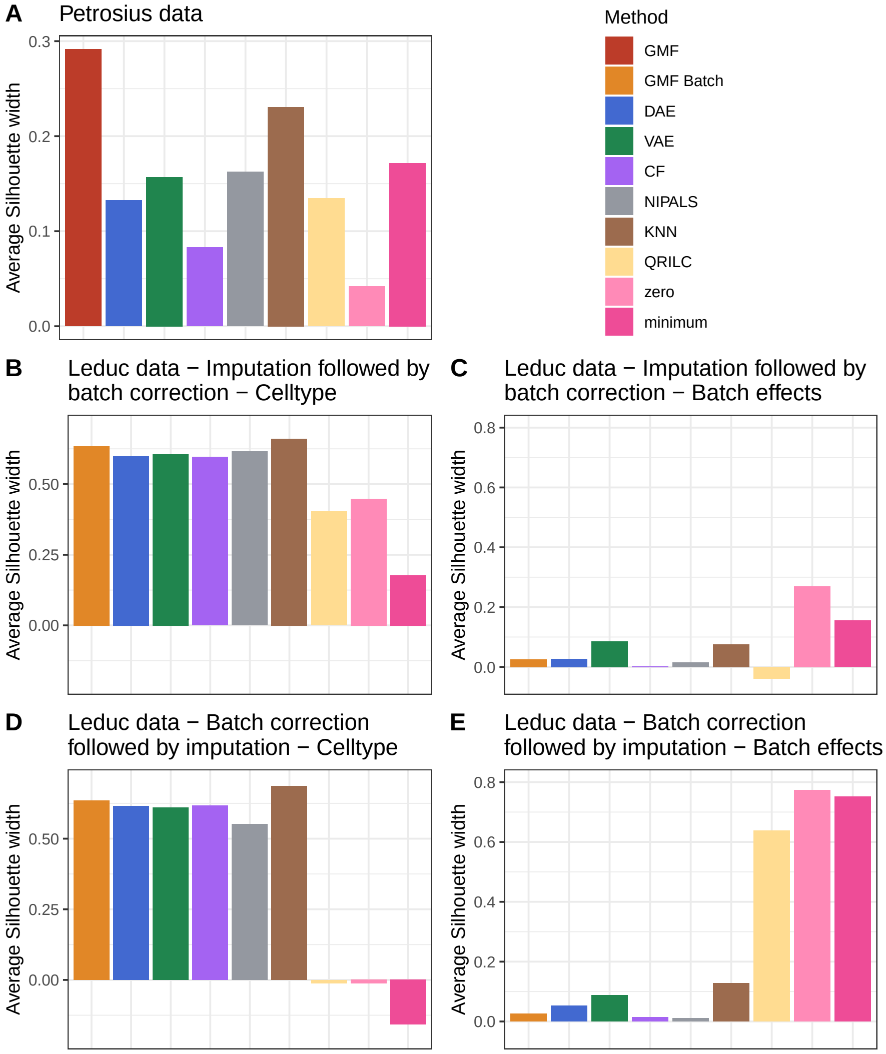

Supp. Fig. 7 Average silhouette width (ASW) on the first two principal components for A) The Petrosius dataset [1] and B-E) the Leduc dataset [2]. Panels A, B and D show ASW for different cell types, with higher ASW indicating better separation between different cell types, therefore resulting in better clustering. Panel C and E show mean ASW for batch evaluated in each cell type separately. Here, lower ASW indicates less separation of different batches within each cell type, thus indicating better clustering, as cells from the same batch should not cluster together after batch correction. omicsGMF (GMF) and omicsGMF while correcting for known batch effects (GMF Batch) directly infer the first two principal components, while the other methods perform imputation followed by PCA (Panel A), imputation followed by batch correction and PCA (Panel B and C) and batch correction followed by imputation and PCA (Panel D and E). Source data are provided as a Source Data file.

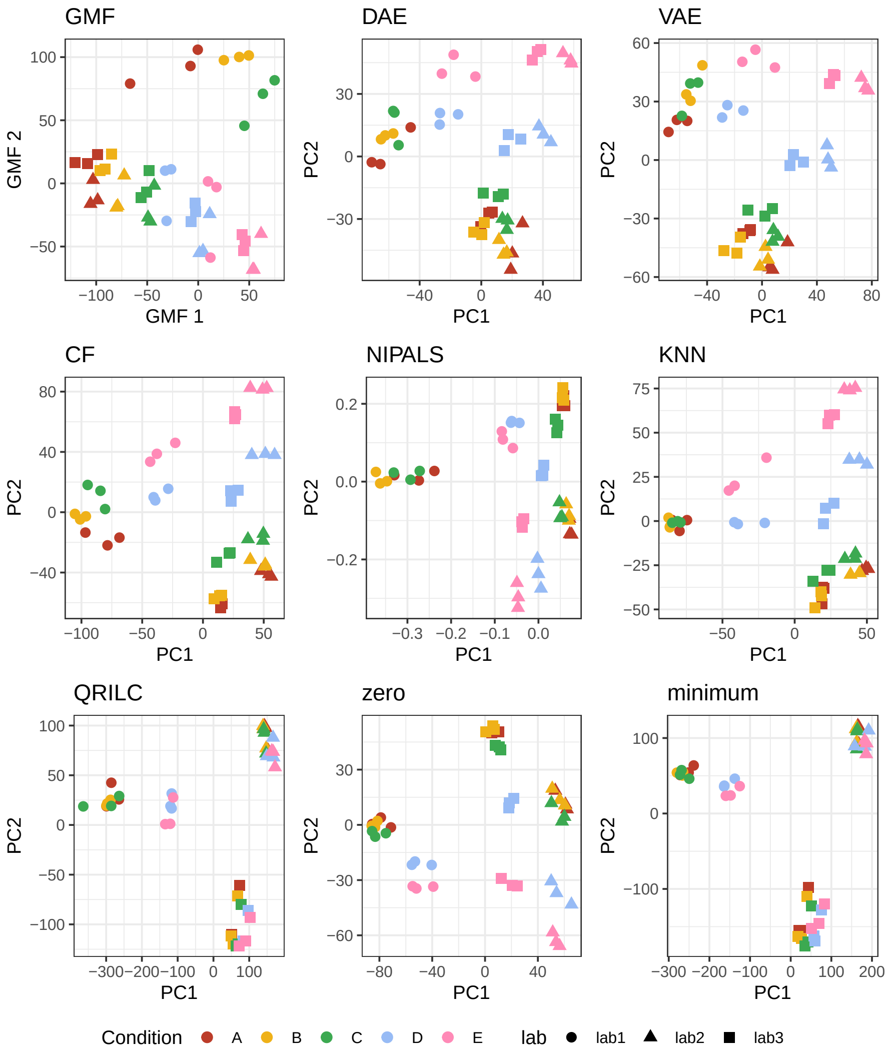

Supp. Fig. 8 Low-dimensional visualization of the complete CPTAC data [3], colored by spike-in concentration of human proteins. Distinct marker shapes indicate the different labs. omicsGMF and NIPALS directly estimate latent factors that have a similar interpretation as regular PCA. The other visualizations are obtained by imputation of missing values using DAE, VAE, CF, KNN, QRILC, zero and minimum, followed by PCA. Source data are provided as a Source Data file.

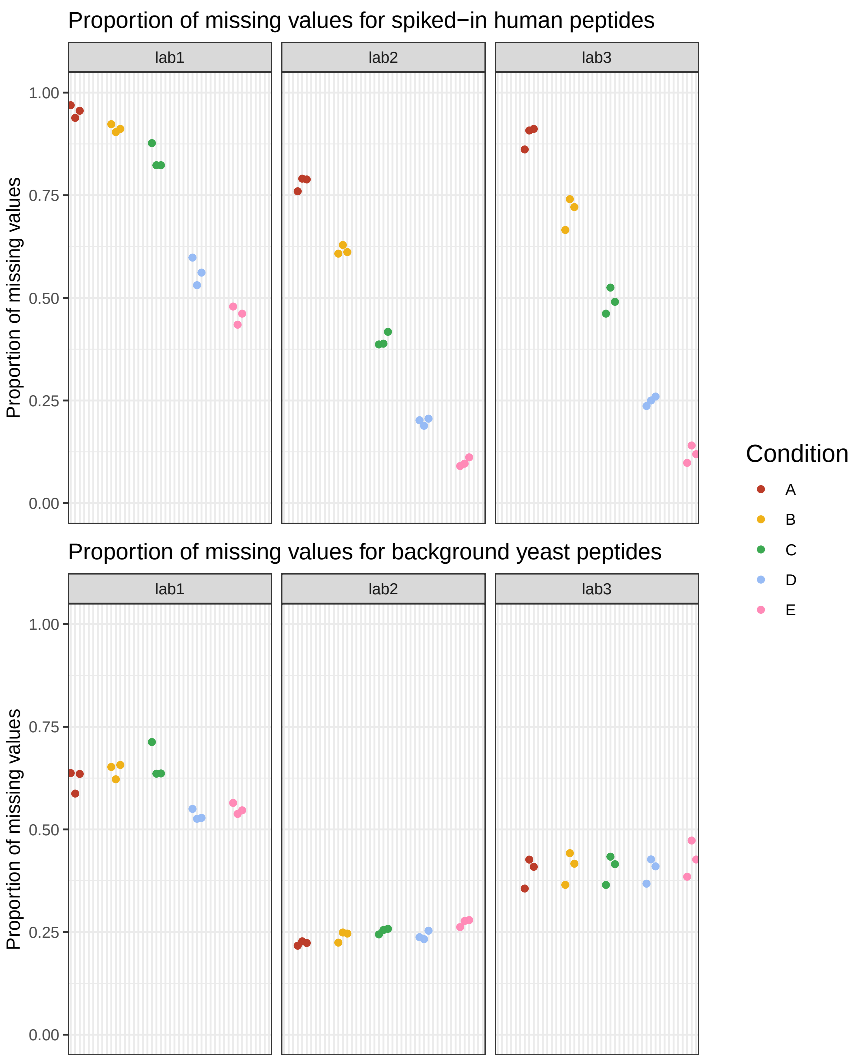

Supp. Fig. 9 Proportion of missing values for each sample of the complete CPTAC dataset [3], for spike-in human peptides (top) and the background yeast peptides (bottom) respectively. The proportion of missing values is represented in function of the spike-in concentration, and is stratified per lab. Source data are provided as a Source Data file.

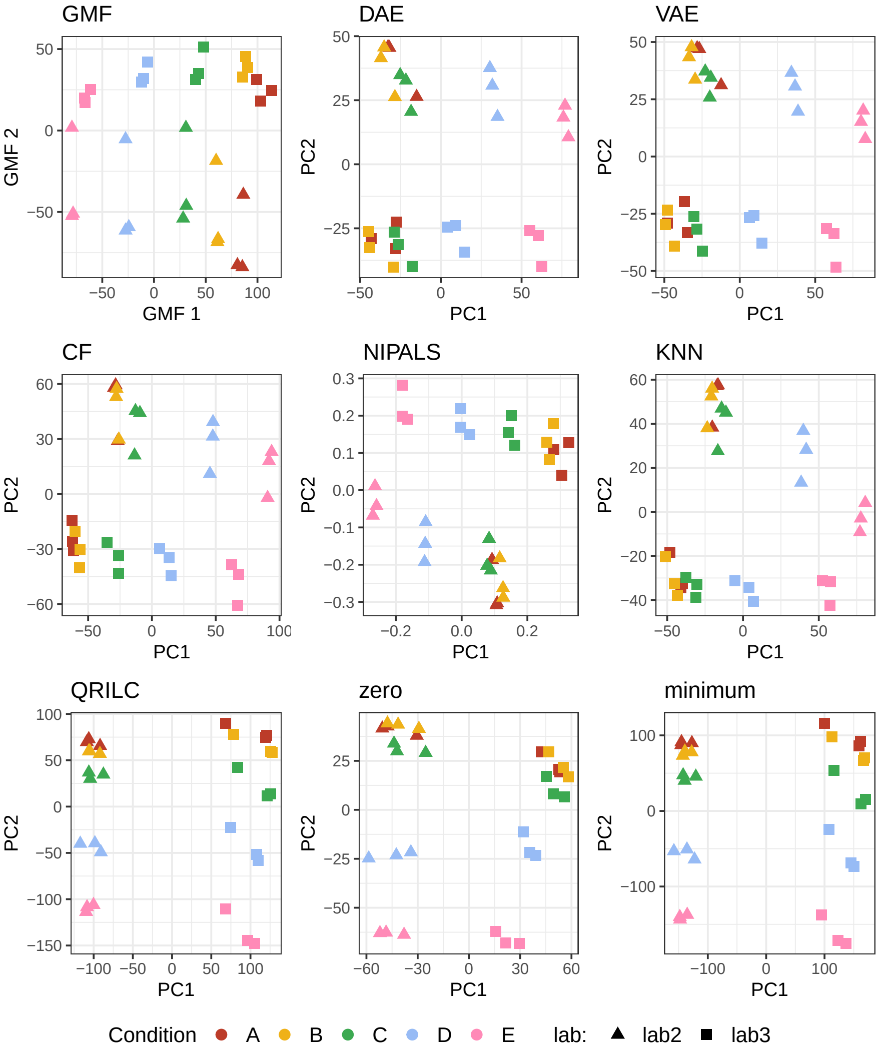

Supp. Fig. 10 Low-dimensional visualization of the CPTAC data [3] upon excluding Lab 1, which was reported to suffer from ionization issues. Samples are colored by spike-in concentration of human proteins, and different labs are represented with a distinct marker shape. omicsGMF and NIPALS directly estimate latent factors that have a similar interpretation as regular PCA. The other visualizations are obtained by imputation of missing values using DAE, VAE, CF, KNN, QRILC, zero and minimum, followed by PCA. Source data are provided as a Source Data file.

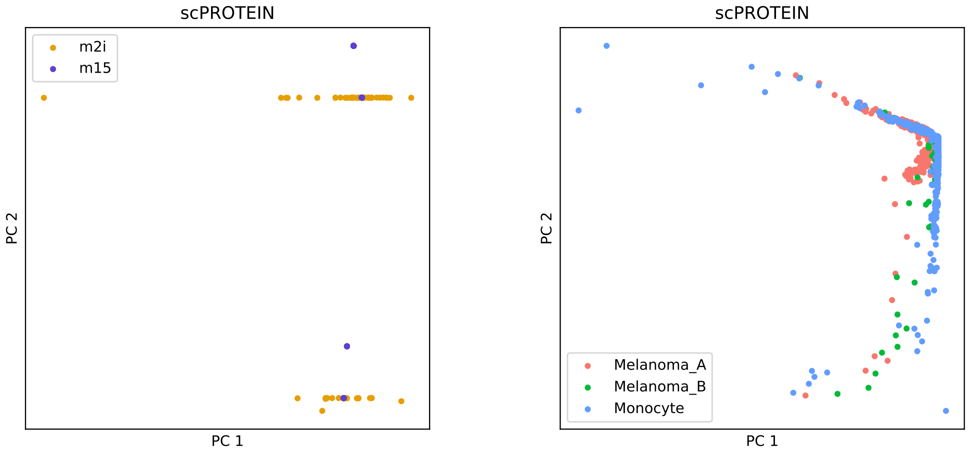

Supp. Fig. 11 Low-dimensional visualization of the Petrosius [1] (left), and Leduc [2] (right) data by the scPROTEIN [4] workflow. Samples are coloured by inhibitor treatment and cell types respectively. No sensible embeddings are obtained by scPROTEIN, which could be due to errors in its initial training step estimating the quality of the signal of each cell. Source data are provided as a Source Data file.

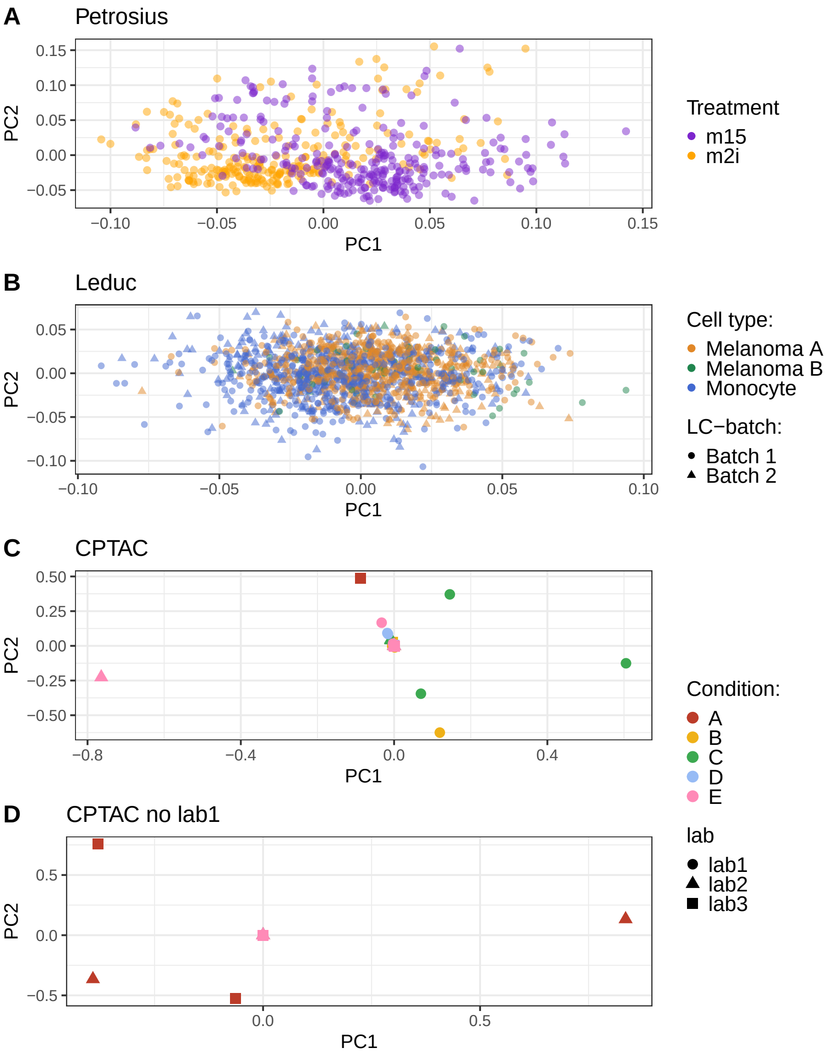

Supp. Fig. 12 Panels A, B, C and D show data from the label-free, single-cell Petrosius study [1], the labeled single cell Leduc dataset [2], the data from the label-free bulk CPTAC spike-in study [3], and the CPTAC spike-in study (data from Lab 1 excluded due to ionization issues). All visualizations are obtained with expectation-maximization PCA (EM-PCA). Note, however, that EM-PCA did not converge for the Leduc and CPTAC data. Source data are provided as a Source Data file.

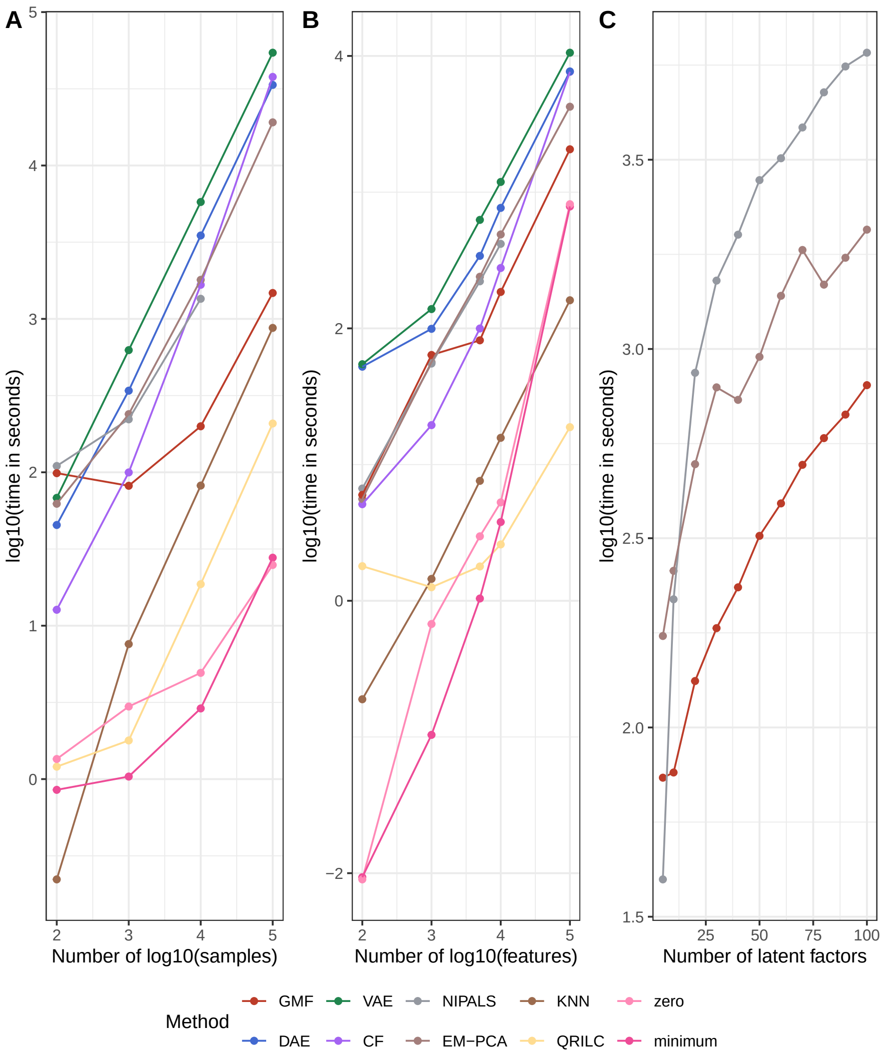

Supp. Fig. 13 Benchmark of computational time (in log10 of the number of seconds) in function of the number of samples (Panel A), the number of features (Panel B) and the number of latent factors for PCA-like algorithms (Panel C). Thousand samples are used in Panel B and C, five thousand features are used in Panel A and C, and ten latent factors are used in Panel A and B. omicsGMF is much faster compared to PCA-like algorithms that can deal with missing values (NIPALS and EM-PCA), and is faster than PIMMS imputation (DAE, VAE and CF) on large datasets. Methods like KNN, QRILC, zero and minimum imputation are faster than omicsGMF, but do not account for the correlation structure in the data. NIPALS could not return results in Panel A for 100000 samples. Source data are provided as a Source Data file.

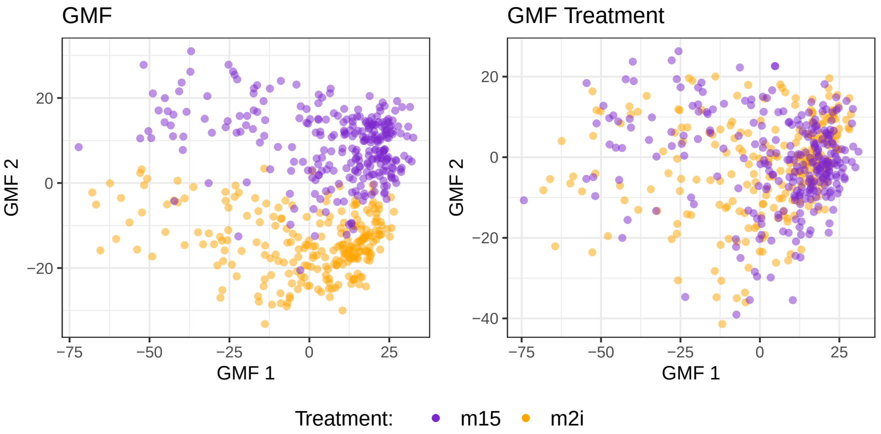

Supp. Fig. 14 Low-dimensional visualization of the Petrosius data [1], colored by inhibitor treatment. omicsGMF is used both without (left) and with (right) a dummy variable for the inhibitor treatment. Clearly, the treatment effect is filtered out if this known covariate is accounted for, resulting in visualizations that control for this effect. This showcases the use of omicsGMF when differently treated cells have to be clustered together. Source data are provided as a Source Data file.

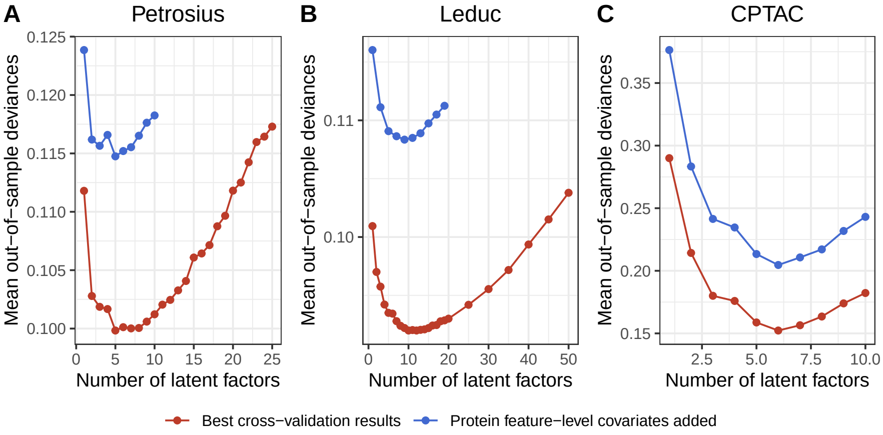

Supp. Fig. 15 Cross-validation with omicsGMF allows for comprehensive selection of the number of latent factors d in $\mathbf{U}$ for dimensionality reduction in combination with selection of known sample- or feature-level covariates. Each panel shows the mean of the out-of-sample deviances over three cross-validation folds in function of the number of latent factors d included in the model. In each fold, 30% of the values are masked for out-of-sample prediction. Panel A shows the cross-validation results for the Petrosius dataset [1], Panel B shows the cross-validation results for the Leduc dataset [2], and Panel C shows the cross-validation results for the CPTAC data [3], considering all three labs. Results are shown for the best cross-validation obtained for omicsGMF in Figure 4, i.e., without known sample-level covariates for Petrosius, with correction for the known batch-effect associated to multiplexing cells in the same run for Leduc (142 dummy variables) and without correcting for lab- and spike-in effects for CPTAC. Then, feature-level covariates, i.e., dummy variables for the protein to which peptides belong are added to these models. Cross-validation provides clear evidence against including these covariates in all three datasets. Source data are provided as a Source Data file.

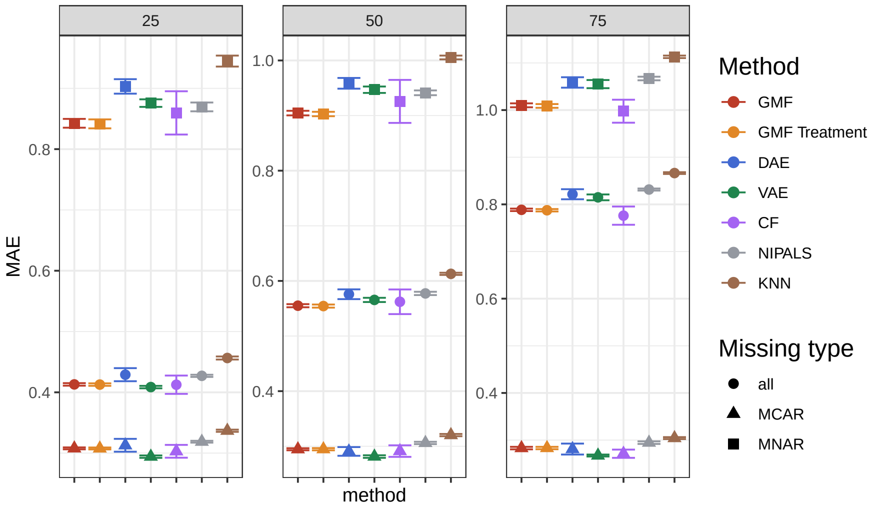

Supp. Fig. 16 Mean absolute error (MAE) of the imputed values in the Petrosius dataset [1] evaluated for omicsGMF (GMF), omicsGMF including a dummy variable for the treatment effect (GMF Treatment), DAE, VAE, CF, NIPALS and KNN-imputation. Missing values were simulated according to the procedure described by [5] (see Methods), which introduces both missing completely at random (MCAR) and missing not at random (MNAR) values in predefined proportions. In this study, the proportions of MNAR masked values were set to 25% (left), 50% (middle), and 75% (right). For each condition, 10 different random seeds were used, and the mean MAE across these seeds is shown, with error bars representing the standard error of the MAE. The MAE was calculated exclusively for masked values based on the difference between the imputed values and the original observed values prior to masking. Distinct marker shapes indicate the MAE for only MCAR masked values, MNAR masked values, and for all masked values combined (all). Source data are provided as a Source Data file.

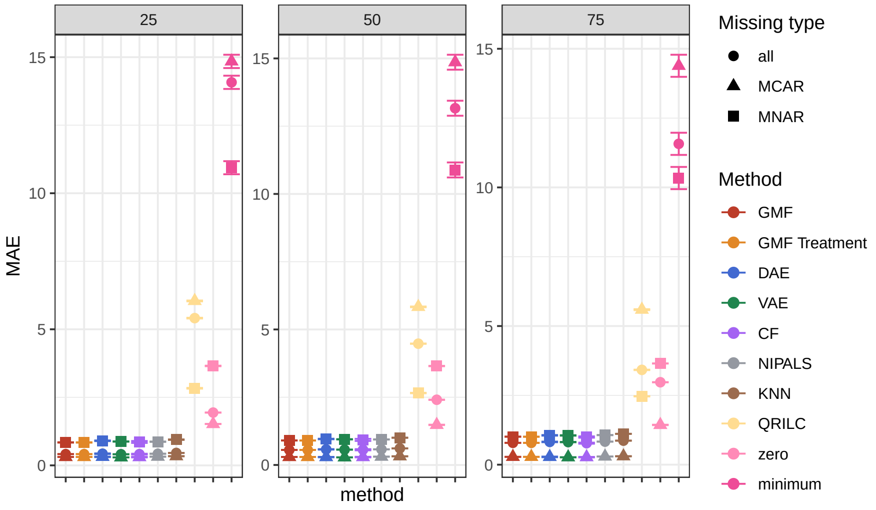

Supp. Fig. 17 Same figure as Supp. Fig 16, but with QRILC, zero and minimum imputation. Source data are provided as a Source Data file.

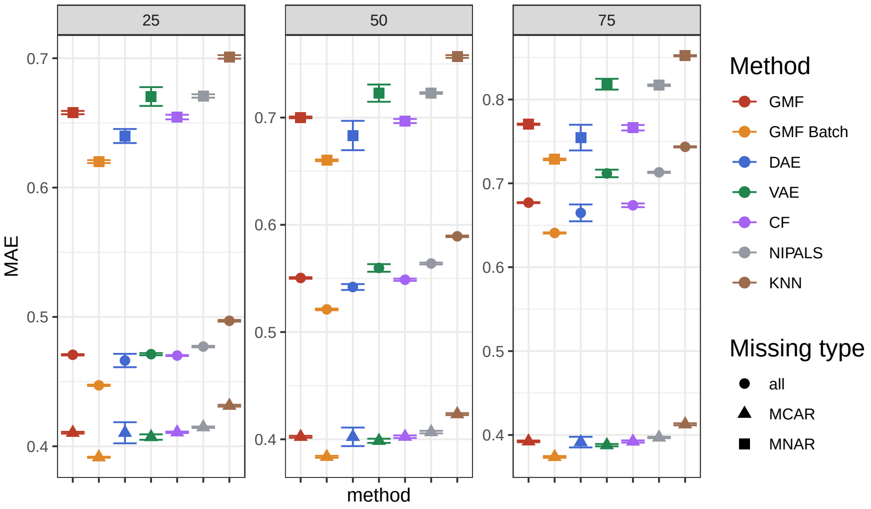

Supp. Fig. 18 Mean absolute error (MAE) of the imputed values in the Leduc dataset [2] evaluated for omicsGMF (GMF), omicsGMF including 142 dummy variables for the batch effects (GMF Batch), DAE, VAE, CF, NIPALS and KNN-imputation. Missing values were simulated according to the procedure described by [5] (see Methods), which introduces both missing completely at random (MCAR) and missing not at random (MNAR) values in predefined proportions. In this study, the proportions of MNAR masked values were set to 25% (left), 50% (middle), and 75% (right). For each condition, 10 different random seeds were used, and the mean MAE across these seeds is shown, with error bars representing the standard error of the MAE. The MAE was calculated exclusively for masked values based on the difference between the imputed values and the original observed values prior to masking. Distinct marker shapes indicate the MAE for only MCAR masked values, MNAR masked values, and for all masked values combined (all). Source data are provided as a Source Data file.

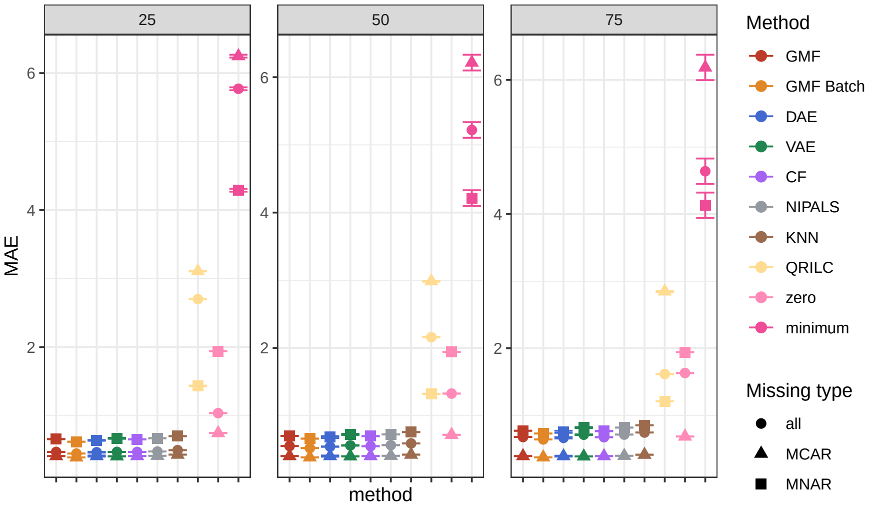

Supp. Fig. 19 Same figure as Supp. Fig 18, but with QRILC, zero and minimum imputation. Source data are provided as a Source Data file.

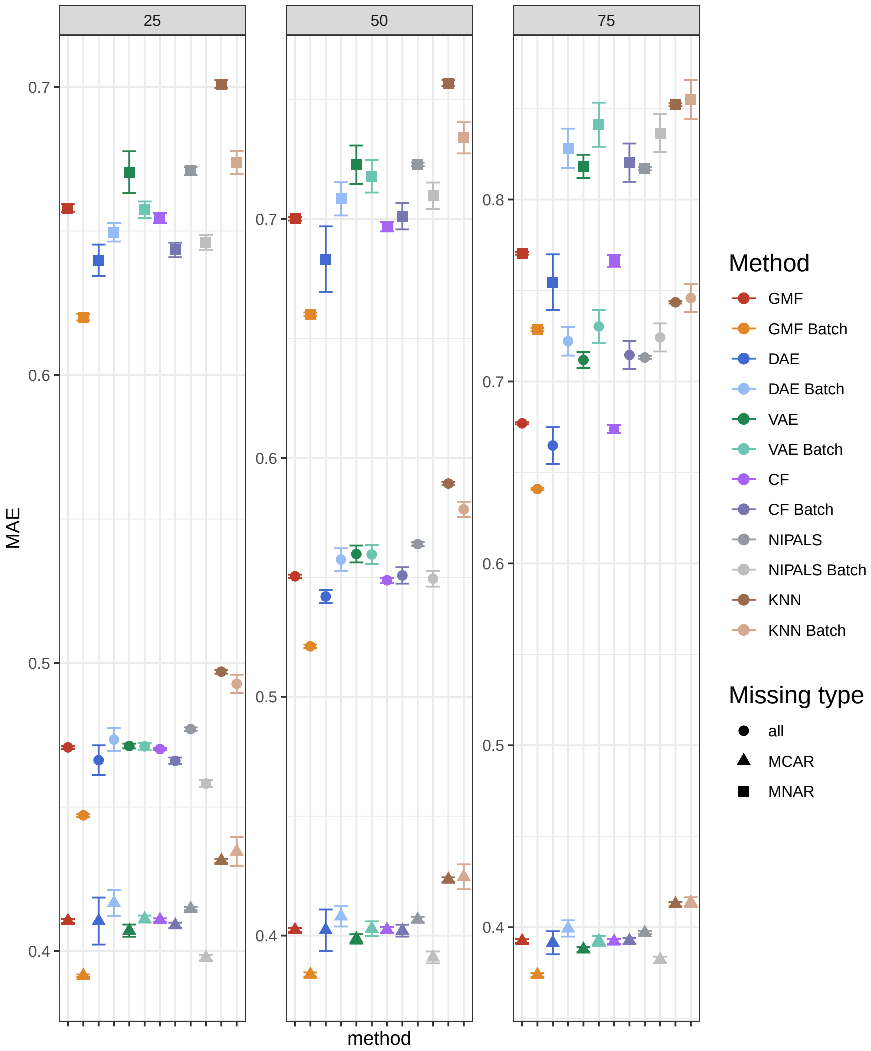

Supp. Fig. 20 Same figure as Supp. Fig 18, but with additional workflows in which batch effects were removed prior to imputation by linear regression including 142 dummy variables indicating TMT-run, except for omicsGMF that performs batch correction internally. These additional workflows are named with 'Batch'. Source data are provided as a Source Data file.

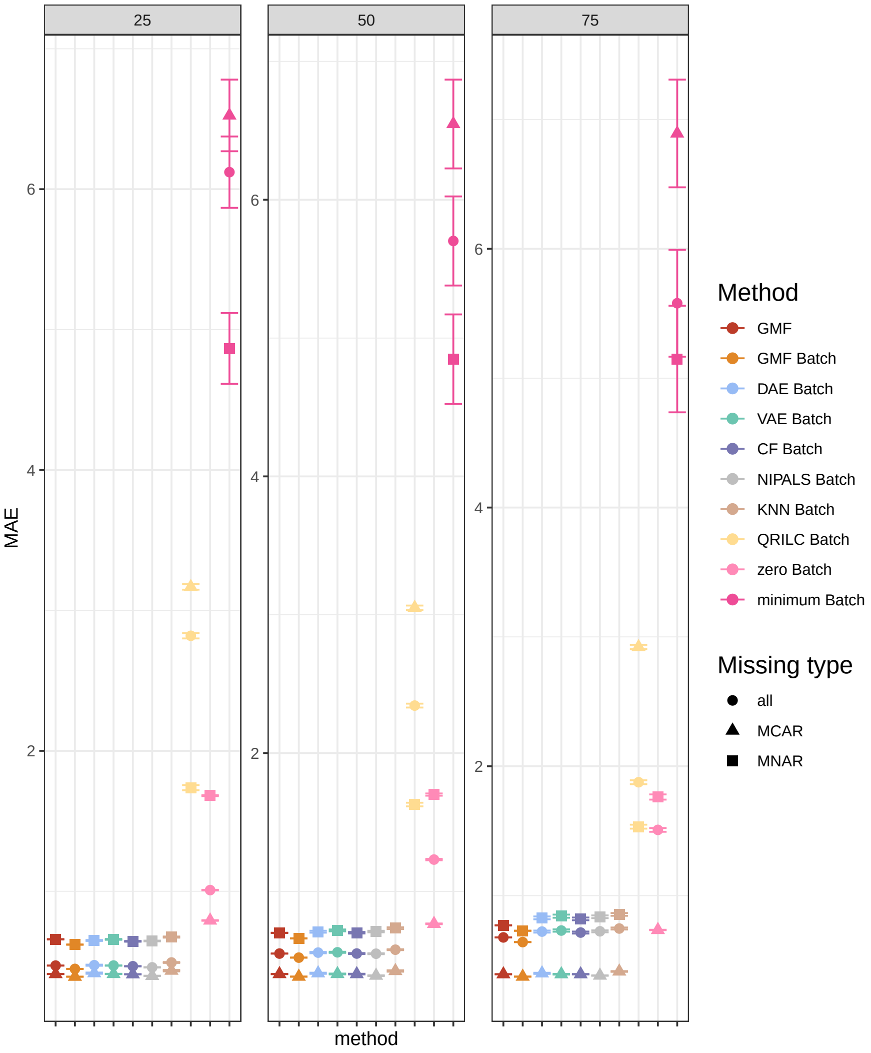

Supp. Fig. 21 Similar figure as Supp. Fig 20, but with QRILC, zero and minimum imputation. Source data are provided as a Source Data file.

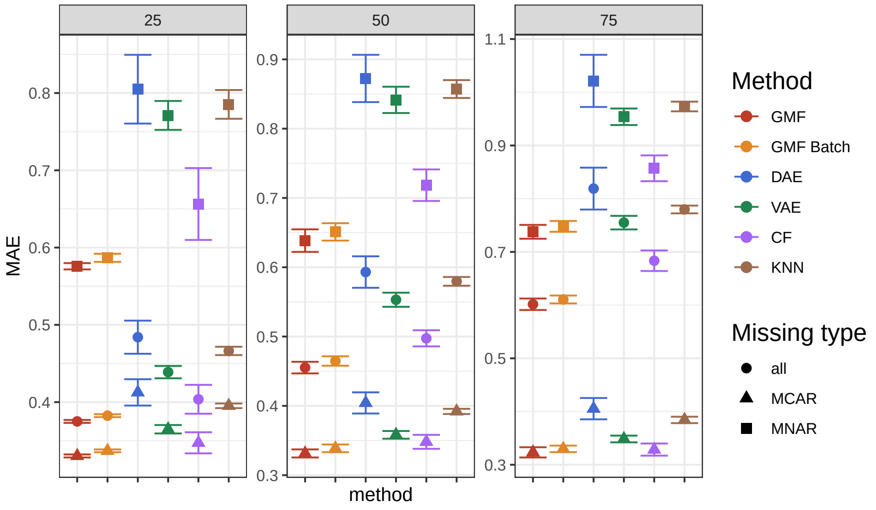

Supp. Fig. 22 Mean absolute error (MAE) of the imputed values in the complete CPTAC dataset [3] evaluated for omicsGMF (GMF), omicsGMF including 2 dummy variables for the batch effects (GMF Batch), DAE, VAE, CF and KNN-imputation. NIPALS was not included due to convergence issues. Missing values were simulated according to the procedure described by [5] (see Methods), which introduces both missing completely at random (MCAR) and missing not at random (MNAR) values in predefined proportions. In this study, the proportions of MNAR masked values were set to 25% (left), 50% (middle), and 75% (right). For each condition, 10 different random seeds were used, and the mean MAE across these seeds is shown, with error bars representing the standard error of the MAE. The MAE was calculated exclusively for masked values based on the difference between the imputed values and the original observed values prior to masking. Distinct marker shapes indicate the MAE for only MCAR masked values, MNAR masked values, and for all masked values combined (all). Source data are provided as a Source Data file.

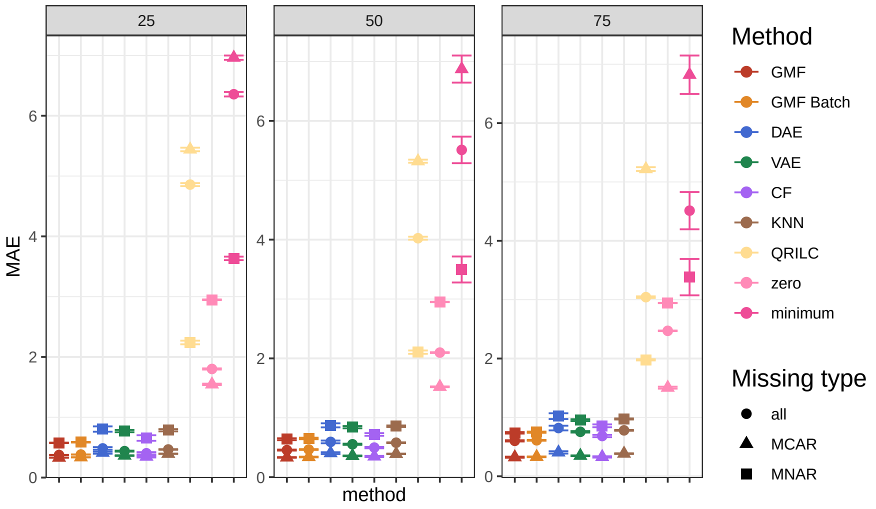

Supp. Fig. 23 Same figure as Supp. Fig 22, but with QRILC, zero and minimum imputation. Source data are provided as a Source Data file.

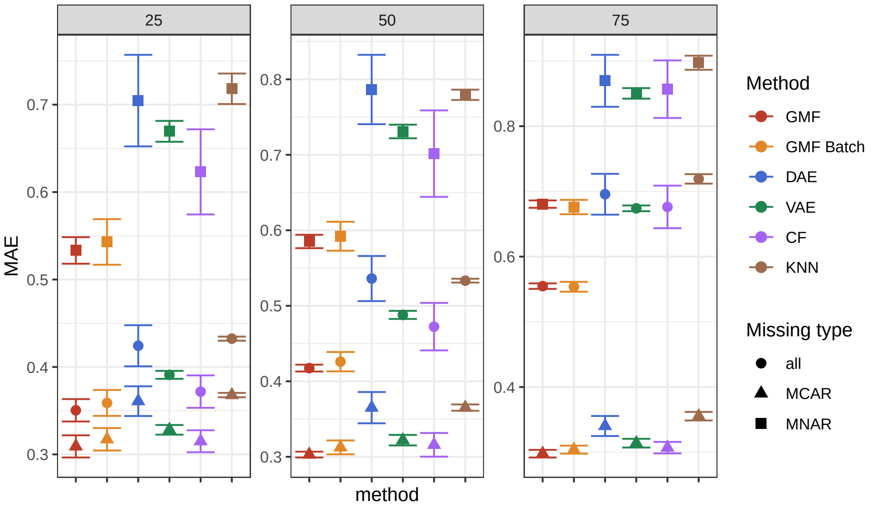

Supp. Fig. 24 Mean absolute error (MAE) of the imputed values in the CPTAC dataset [3], excluding data from Lab 1 that suffered from ionization issues. The MAE is evaluated for omicsGMF (GMF), omicsGMF including a dummy variable for the batch effects (GMF Batch), DAE, VAE, CF and KNN-imputation. NIPALS was not included due to convergence issues. Missing values were simulated according to the procedure described by [5] (see Methods), which introduces both missing completely at random (MCAR) and missing not at random (MNAR) values in predefined proportions. In this study, the proportions of MNAR masked values were set to 25% (left), 50% (middle), and 75% (right). For each condition, 10 different random seeds were used, and the mean MAE across these seeds is shown, with error bars representing the standard error of the MAE. The MAE was calculated exclusively for masked values based on the difference between the imputed values and the original observed values prior to masking. Distinct marker shapes indicate the MAE for only MCAR masked values, MNAR masked values, and for all masked values combined (all). Source data are provided as a Source Data file.

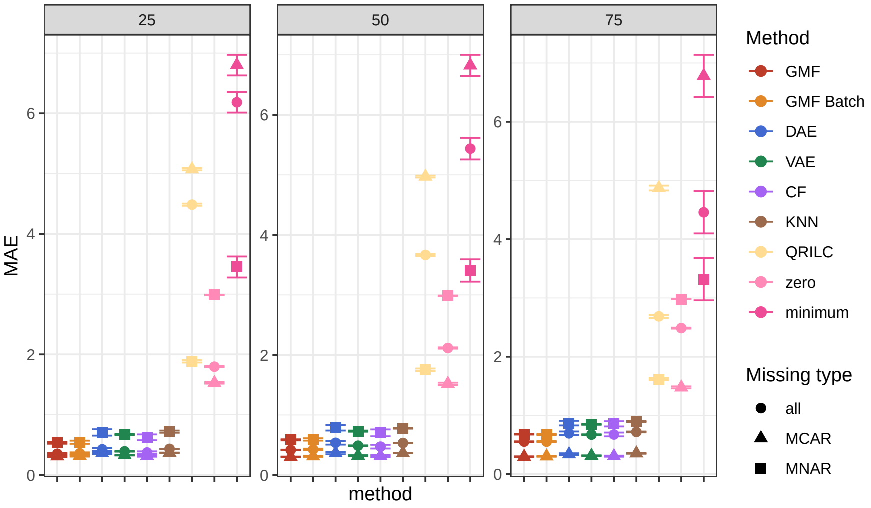

Supp. Fig. 25 Same figure as Supp. Fig 24, but with QRILC, zero and minimum imputation. Source data are provided as a Source Data file.

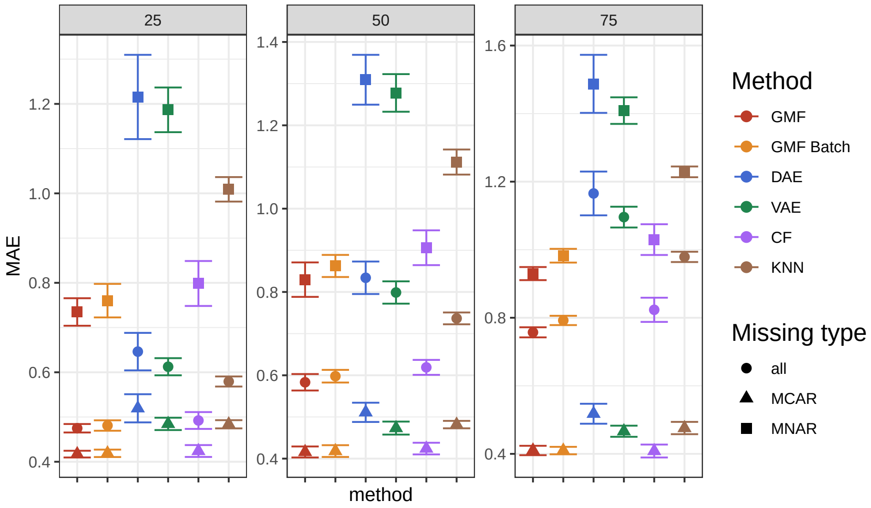

Supp. Fig. 26 Mean absolute error (MAE) for the imputed values from lab 1 of the CPTAC dataset [3] when feeding all data to imputation analysis. The MAE is evaluated for omicsGMF (GMF), omicsGMF including 2 dummy variables for the batch effects (GMF Batch), DAE, VAE, CF and KNN-imputation. NIPALS was not included due to convergence issues. Missing values were simulated according to the procedure described by [5] (see Methods), which introduces both missing completely at random (MCAR) and missing not at random (MNAR) values in predefined proportions. In this study, the proportions of MNAR masked values were set to 25% (left), 50% (middle), and 75% (right). For each condition, 10 different random seeds were used, and the mean MAE across these seeds is shown, with error bars representing the standard error of the MAE. The MAE was calculated exclusively for masked values based on the difference between the imputed values and the original observed values prior to masking. Distinct marker shapes indicate the MAE for only MCAR masked values, MNAR masked values, and for all masked values combined (all). Source data are provided as a Source Data file.

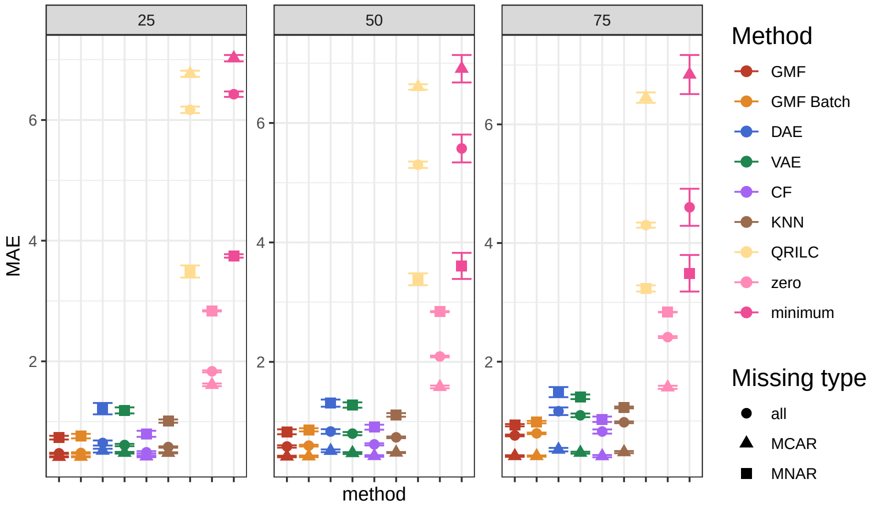

Supp. Fig. 27 Same figure as Supp. Fig 26, but with QRILC, zero and minimum imputation. Source data are provided as a Source Data file.

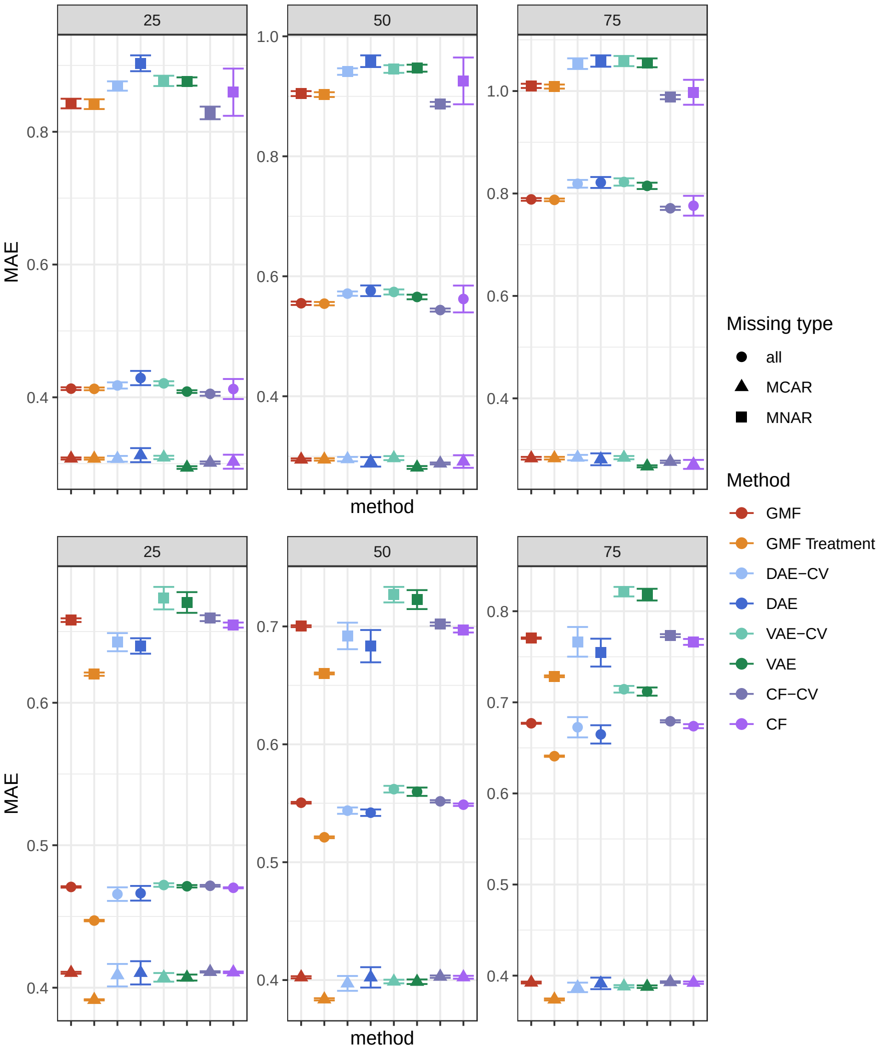

Supp. Fig. 28 Mean absolute error (MAE) of the imputed values in the Petrosius dataset [1] (top) and Leduc dataset [2] (bottom) evaluated for omicsGMF (GMF), omicsGMF including a dummy variable for the treatment effect or 142 dummy variables for the batch effects (GMF Batch), DAE-CV, DAE, VAE-CV, VAE, CF-CV and CF imputation. The methods including 'CV' use the dimensionality of the latent representation suggested by omicsGMF, while the methods without 'CV' use their default values (see Methods). Missing values were simulated according to the procedure described by [5] (see Methods), which introduces both missing completely at random (MCAR) and missing not at random (MNAR) values in predefined proportions. In this study, the proportions of MNAR masked values were set to 25% (left), 50% (middle), and 75% (right). For each condition, 10 different random seeds were used, and the mean MAE across these seeds is shown, with error bars representing the standard error of the MAE. The MAE was calculated exclusively for masked values based on the difference between the imputed values and the original observed values prior to masking. Distinct marker shapes indicate the MAE for only MCAR masked values, MNAR masked values, and for all masked values combined (all). Source data are provided as a Source Data file.

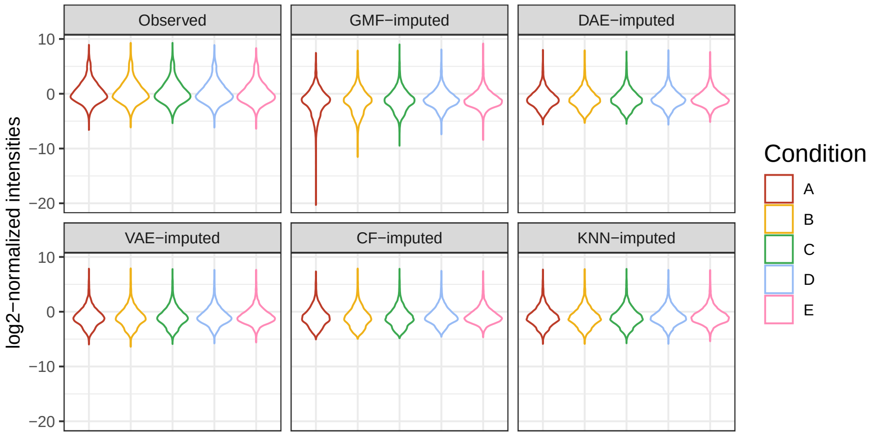

Supp. Fig. 29 Distributions of peptide intensities for background yeast proteins of the CPTAC study, excluding Lab 1 that suffers from ionization issues, stratified according to the spike-in condition. The first panel shows the distribution of observed values, and the other panels show the distributions of imputed intensities by omicsGMF, DAE, VAE, CF and KNN imputation, respectively. Source data are provided as a Source Data file.

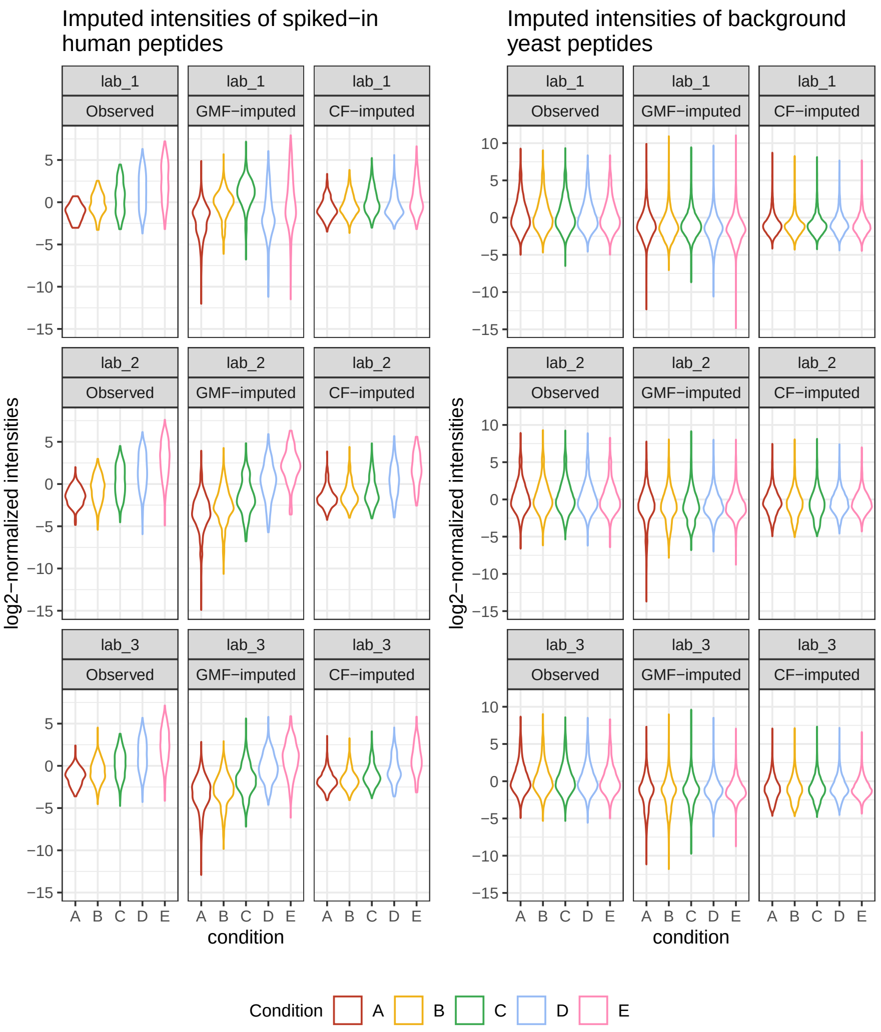

Supp. Fig. 30 Distributions of peptide intensities for human spike-in proteins (left) and background yeast proteins (right) of the complete CPTAC study, stratified according to spike-in condition and lab. The first panel shows the distribution of observed values, and the other panels show the distributions of imputed intensities by omicsGMF and CF, respectively. Source data are provided as a Source Data file.

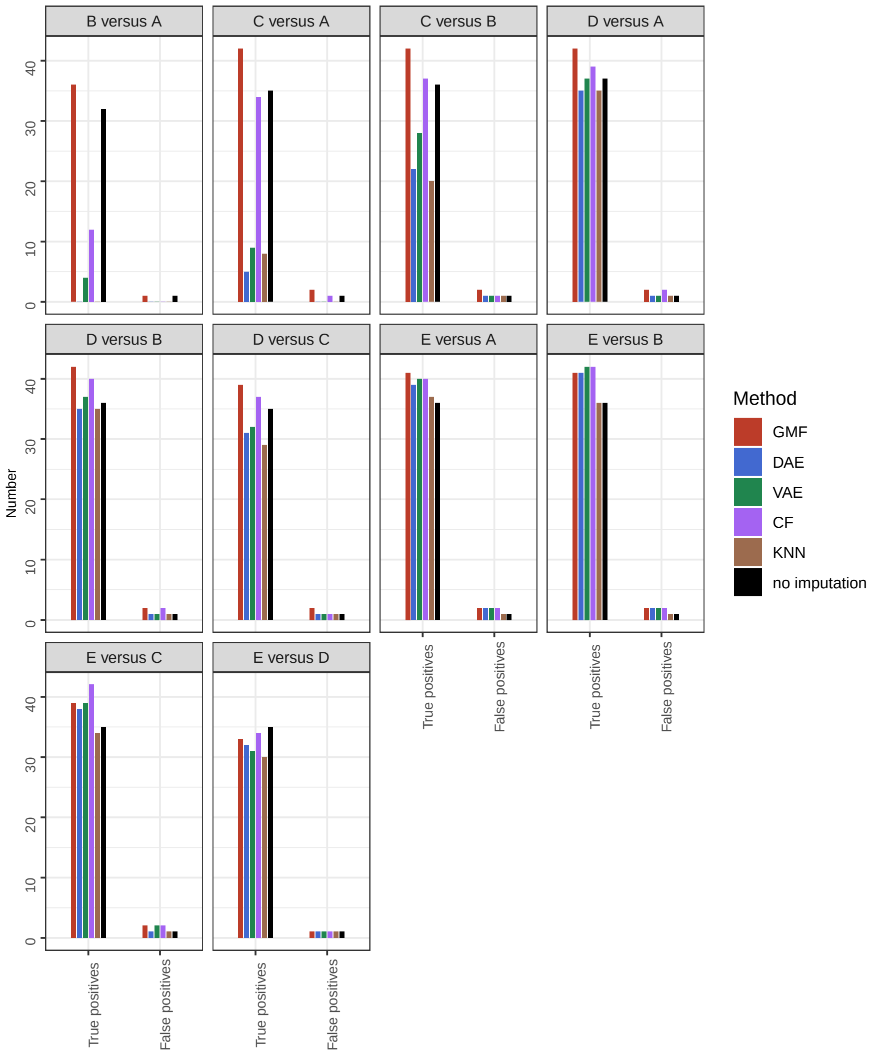

Supp. Fig. 31 Number of true positives and false positives retrieved in the CPTAC dataset [3] at a false discovery proportion of at most 5%. Data from Lab 1 suffering from ionization issues are excluded. Human UPS proteins are differentially spiked between conditions and yeast background proteins serve as true negative control. Source data are provided as a Source Data file.

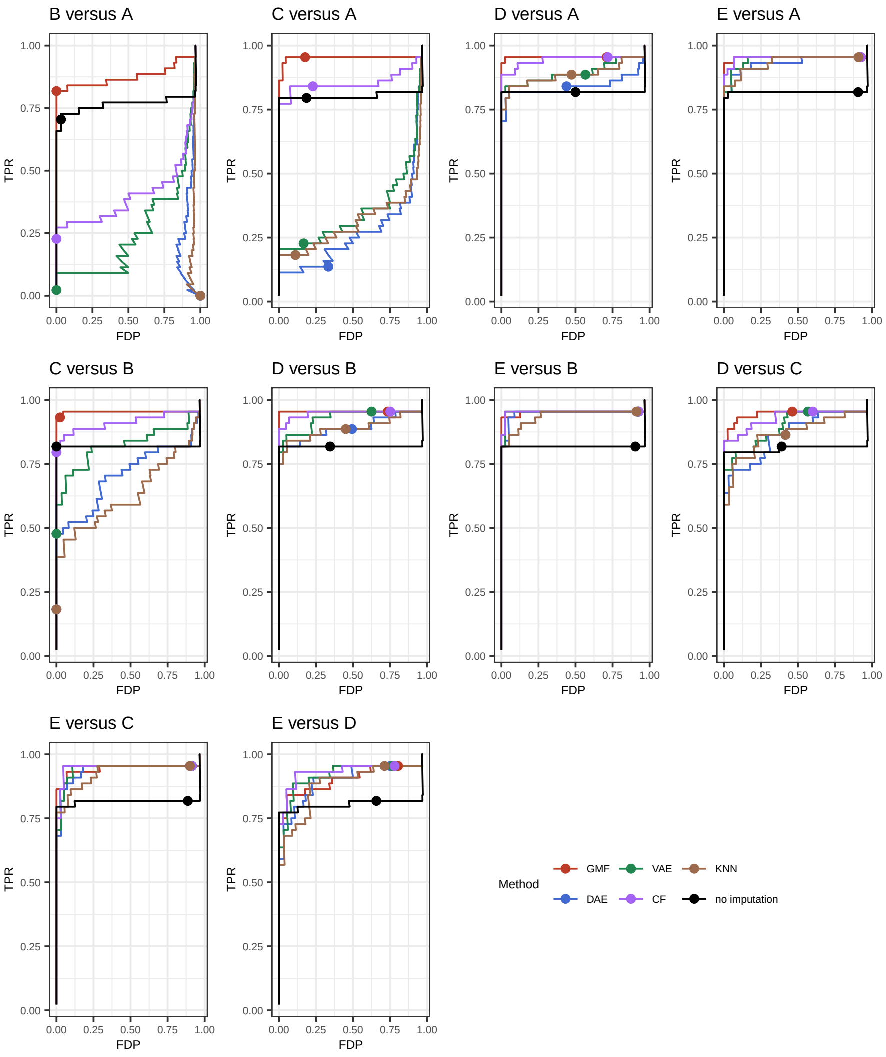

Supp. Fig. 32 Performance evaluation of differential abundance analyses using msqrob2 [6, 7] on the CPTAC dataset [3]. Data from Lab 1 suffering from ionization issues are excluded. The human UPS proteins are differentially spiked between the conditions and the yeast background proteins serve as a true negative control. Each curve shows the true positive rate (TPR) in function of the false discovery proportion (FDP). The dots on each curve represent working points when the false discovery rate level is set at the nominal 5% level. Results for all pairwise comparisons between the spike-in concentrations are shown. Source data are provided as a Source Data file.

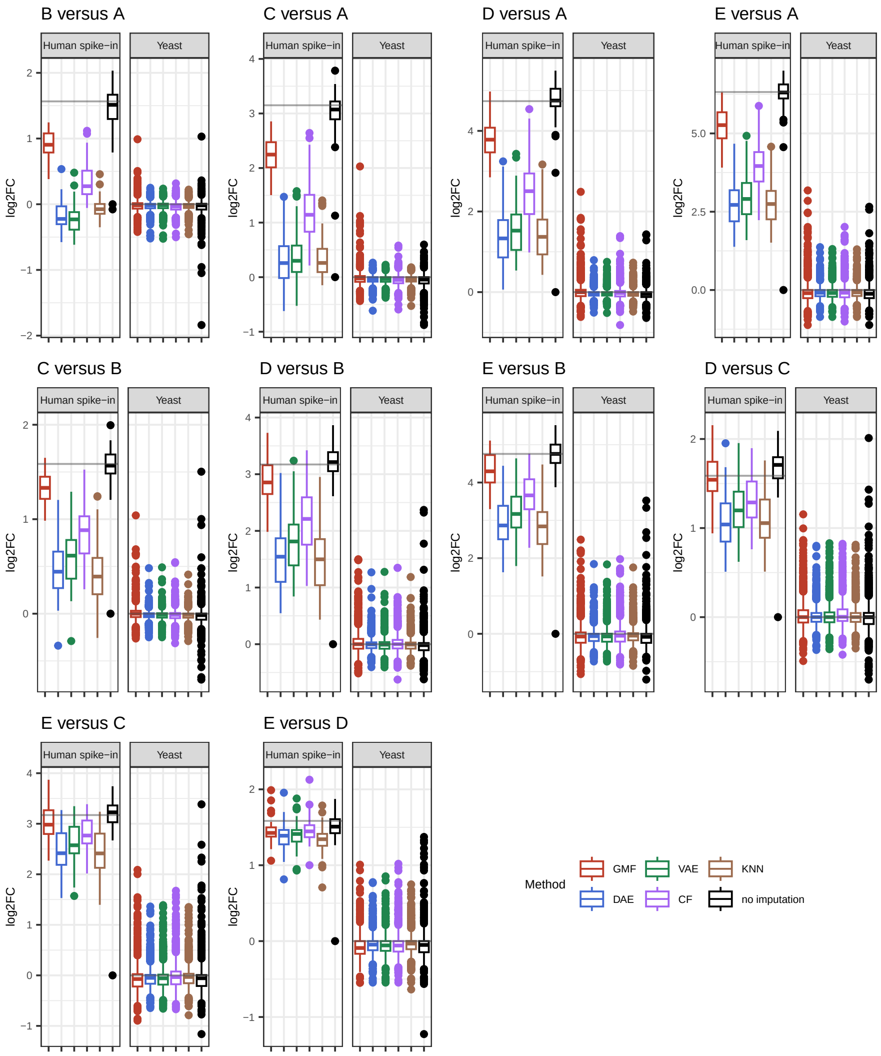

Supp. Fig. 33 Performance evaluation of differential abundance analyses using msqrob2 [6, 7] on the CPTAC dataset [3]. Data from Lab 1 suffering from ionization issues are excluded. The human UPS proteins are differentially spiked between the conditions and the yeast background proteins serve as a true negative control. Each plot shows the estimated log2 fold changes (FC) by msqrob2 for 44 human spike-in proteins, and 1477 reference yeast proteins. The boxplots are defined by the 25, 50, and 75% percentiles. The whiskers have a maximum length of 1.5 times the interquartile range. The grey line indicates the known log2 FC. Source data are provided as a Source Data file.

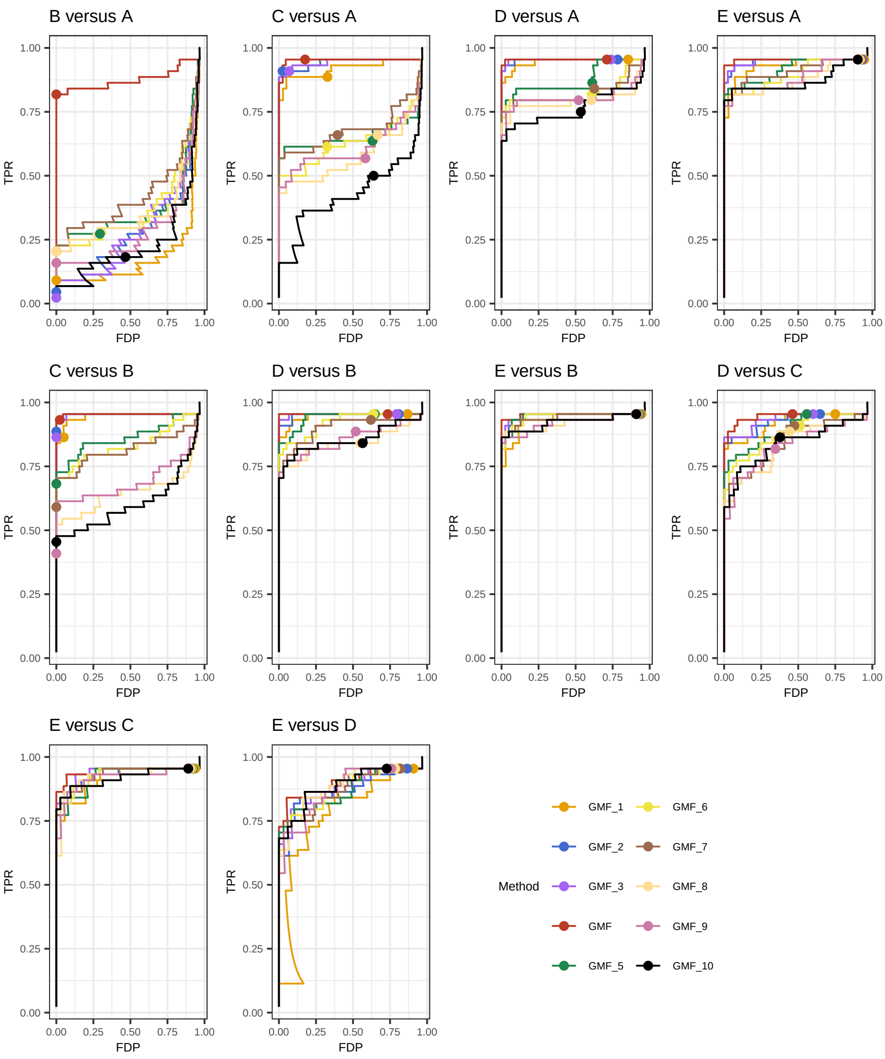

Supp. Fig. 34 Performance evaluation of differential abundance analyses using msqrob2 [6, 7] on the CPTAC dataset [3], for different numbers of latent factors used in omicsGMF. omicsGMF's cross-validation suggested four latent factors. Data from Lab 1, which suffers from ionization issues, are excluded. Human UPS proteins are differentially spiked between conditions while yeast background proteins serve as true negative control. Each curve shows true positive rate (TPR) in function of false discovery proportion (FDP). Dots on each curve represent working points for false discovery rate level at nominal 5% level. Results for all pairwise comparisons between spike-in concentrations are shown. Source data are provided as a Source Data file.

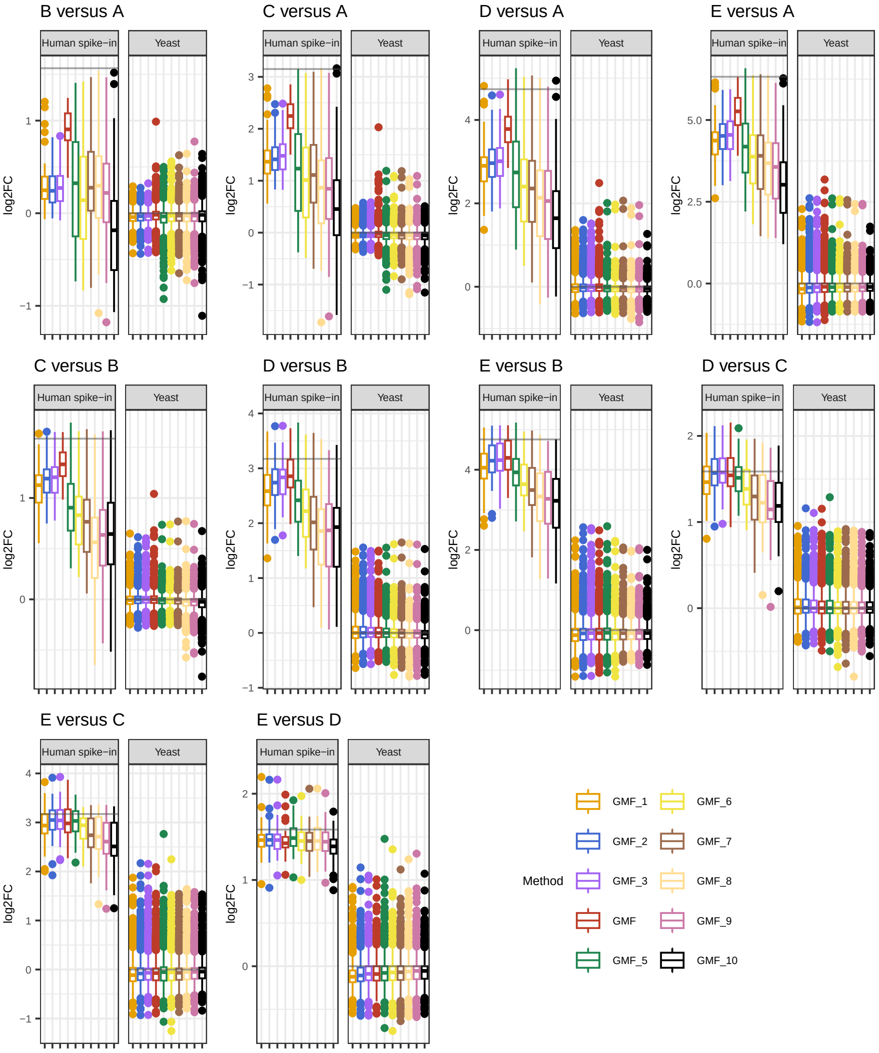

Supp. Fig. 35 Performance evaluation of differential abundance analyses using msqrob2 [6, 7] on the CPTAC dataset [3], for different numbers of latent factors used in omicsGMF. omicsGMF's cross-validation suggested four latent factors. Data from Lab 1, which suffers from ionization issues, are excluded. Human UPS proteins are differentially spiked between conditions while yeast background proteins serve as true negative control. Each plot shows estimated log2 fold changes (FC) by msqrob2 for 44 human spike-in proteins, and 1477 reference yeast proteins. The boxplots are defined by the 25, 50, and 75% percentiles. The whiskers have a maximum length of 1.5 times the interquartile range. Grey line indicates known log2 FC. Source data are provided as a Source Data file.

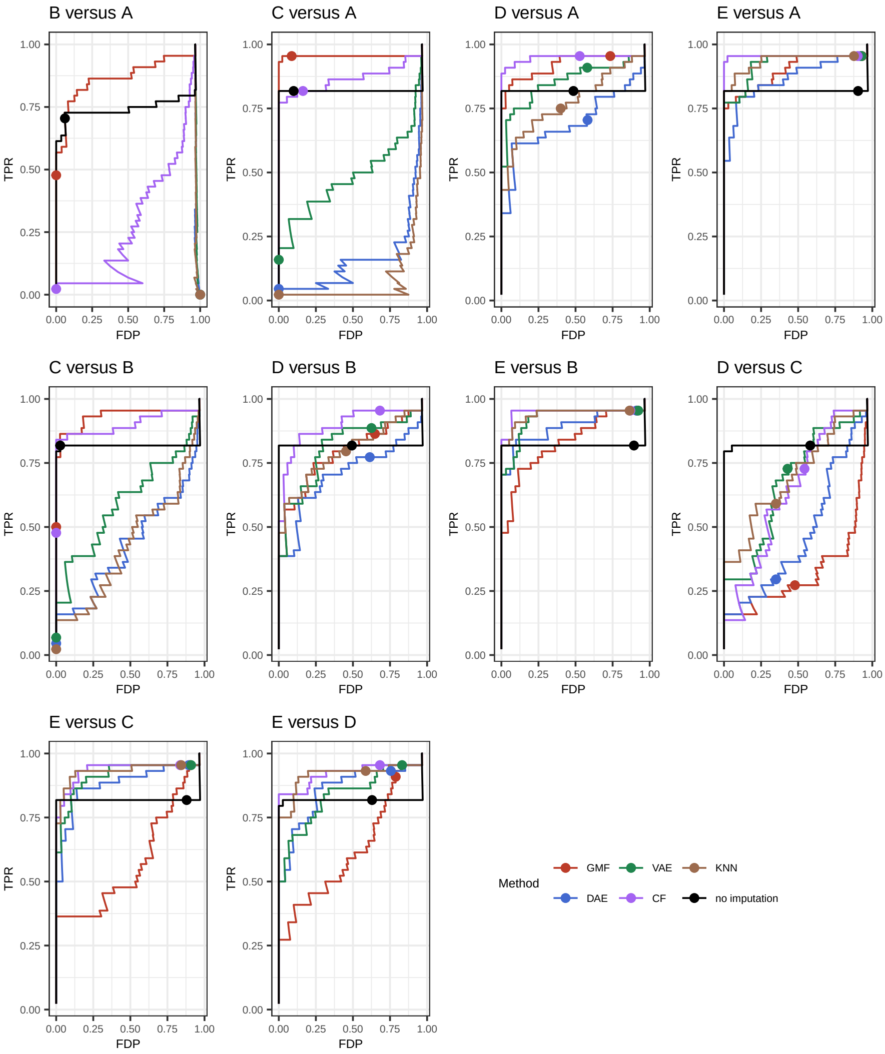

Supp. Fig. 36 Performance evaluation of differential abundance analyses using msqrob2 [6, 7] on the complete CPTAC dataset [3]. The human UPS proteins are differentially spiked between the conditions and the yeast background proteins serve as a true negative control. Each curve shows the true positive rate (TPR) in function of the false discovery proportion (FDP). The dots on each curve represent working points when the false discovery rate level is set at the nominal 5% level. Results for all pairwise comparisons between the spike-in concentrations are shown. Source data are provided as a Source Data file.

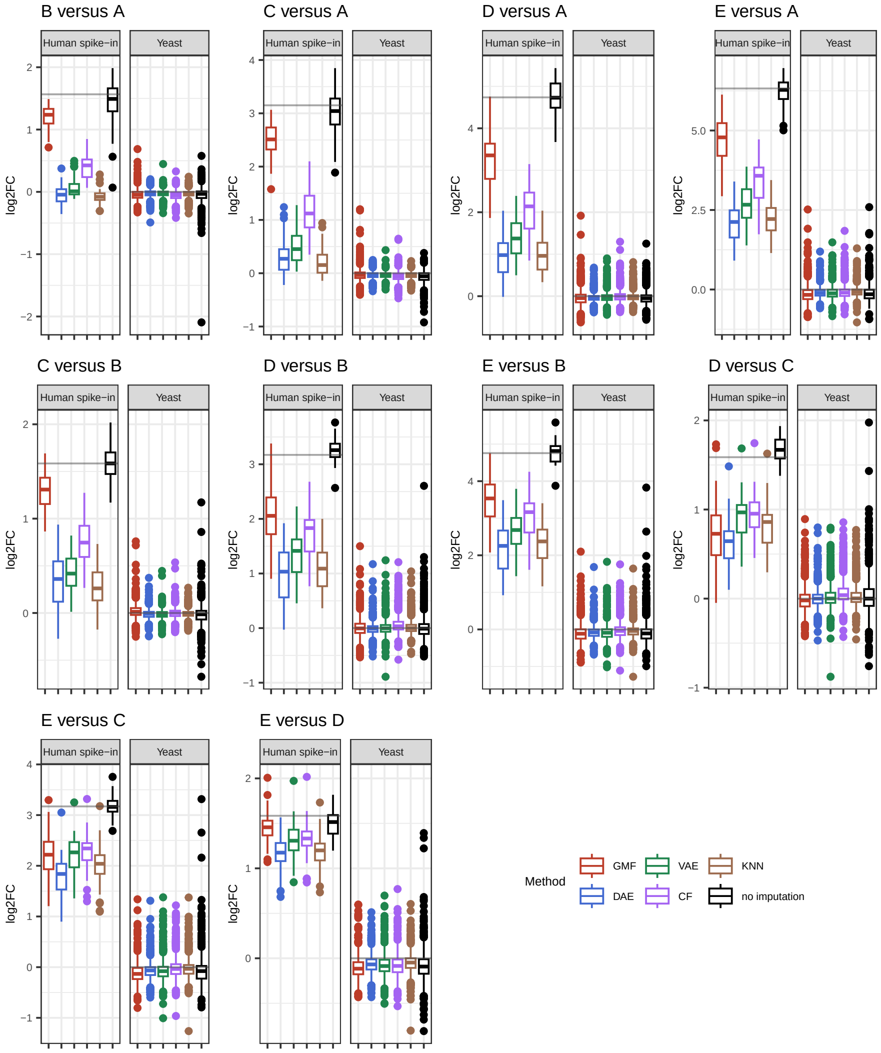

Supp. Fig. 37 Performance evaluation of differential abundance analyses using msqrob2 [6, 7] on the complete CPTAC dataset [3]. The human UPS proteins are differentially spiked between the conditions and the yeast background proteins serve as a true negative control. Each plot shows the estimated log2 fold changes (FC) by msqrob2 for 44 human spike-in proteins, and 1477 reference yeast proteins. The grey line indicates the known log2 FC. The boxplots are defined by the 25, 50, and 75% percentiles. The whiskers have a maximum length of 1.5 times the interquartile range. Source data are provided as a Source Data file.

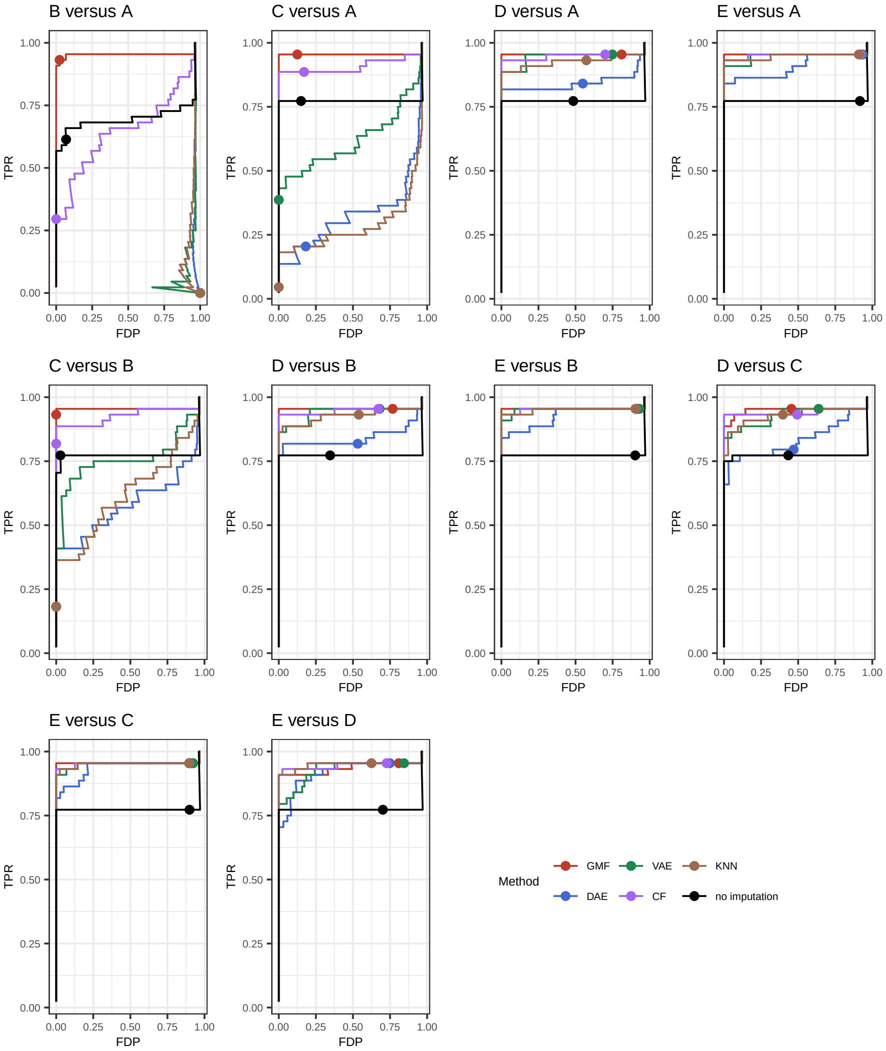

Supp. Fig. 38 Performance evaluation of differential abundance analyses using msqrob2 [6, 7] on the complete CPTAC dataset [3], when including an additional dummy variable for conditions D-E from Lab 1. The human UPS proteins are differentially spiked between the conditions and the yeast background proteins serve as a true negative control. Each curve shows the true positive rate (TPR) in function of the false discovery proportion (FDP). The dots on each curve represent working points when the false discovery rate level is set at the nominal 5% level. Results for all pairwise comparisons between the spike-in concentrations are shown. Source data are provided as a Source Data file.

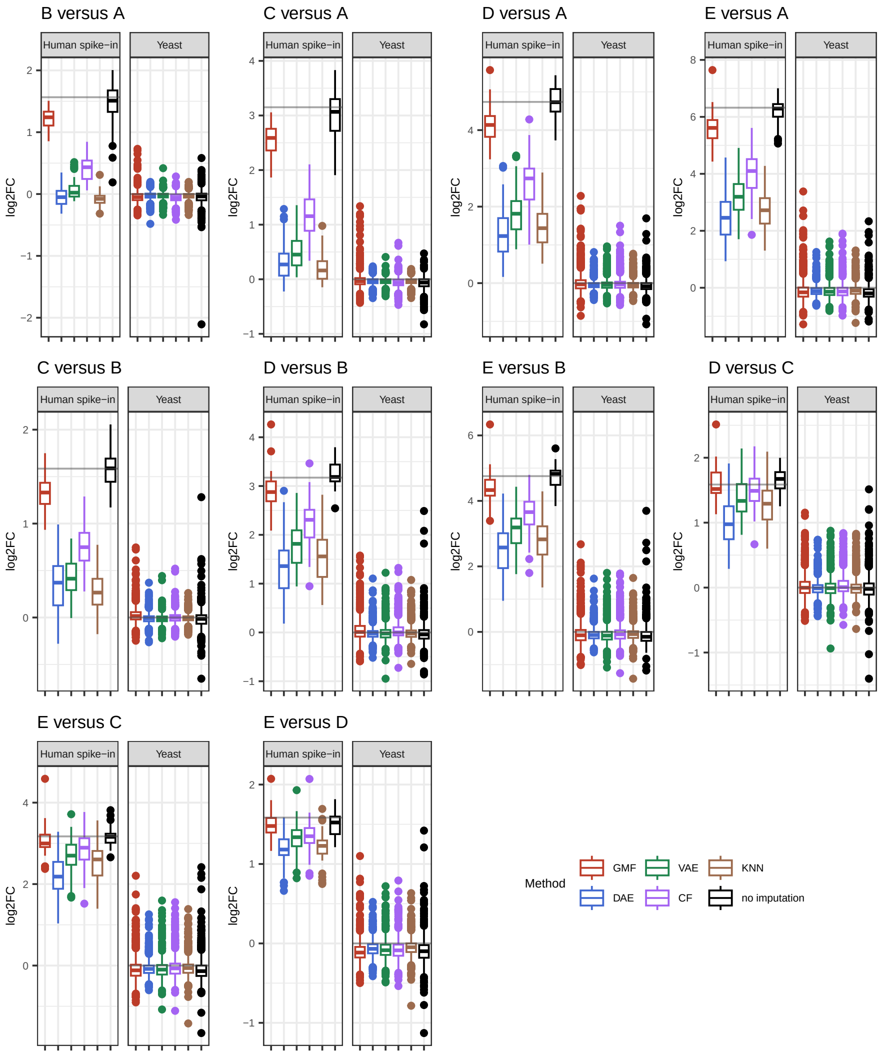

Supp. Fig. 39 Performance evaluation of differential abundance analyses using msqrob2 [6, 7] on the complete CPTAC dataset [3], when including an additional dummy variable for conditions D-E from Lab 1. The human UPS proteins are differentially spiked between the conditions and the yeast background proteins serve as a true negative control. Each plot shows the estimated log2 fold changes (FC) by msqrob2 for 44 human spike-in proteins, and 1477 reference yeast proteins. The grey line indicates the known log2 FC. The boxplots are defined by the 25, 50, and 75% percentiles. The whiskers have a maximum length of 1.5 times the interquartile range. Source data are provided as a Source Data file.

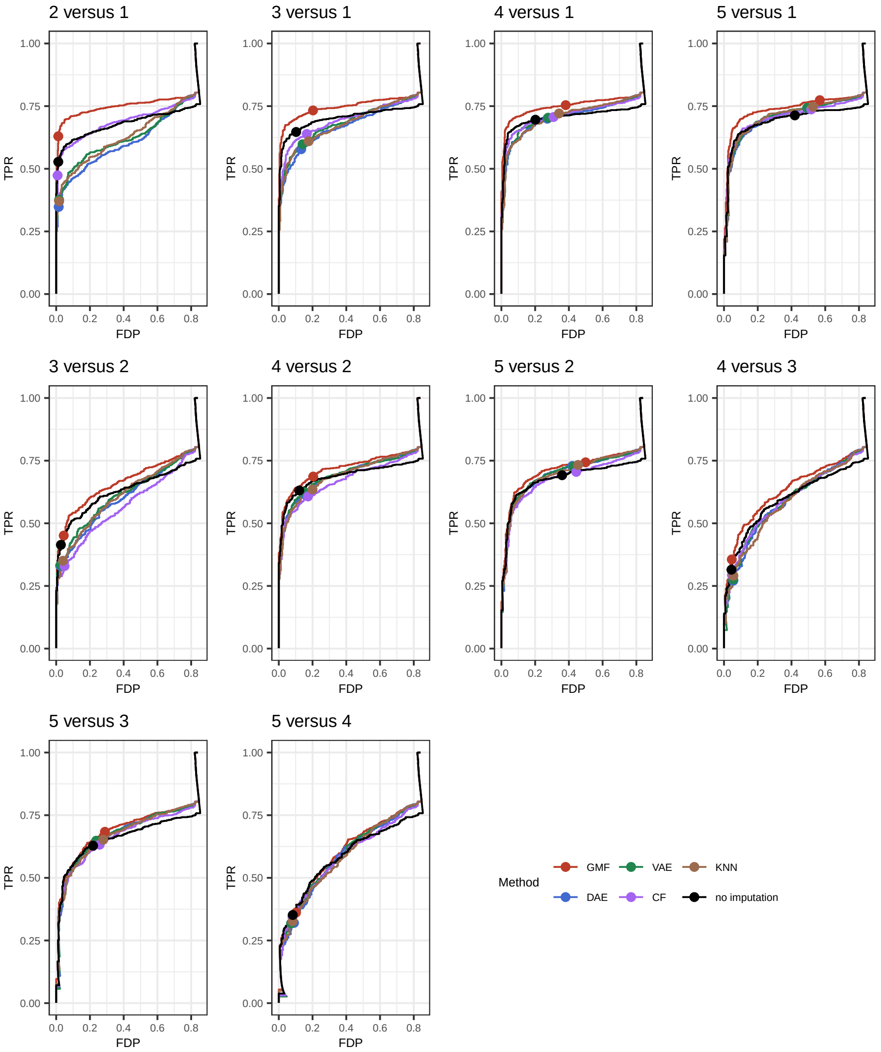

Supp. Fig. 40 Performance evaluation of differential abundance analyses using msqrob2 [6, 7] on the Shen dataset [8]. The *E. coli* proteins are mixed in different concentrations within a human background. Each curve shows the true positive rate (TPR) in function of the false discovery proportion (FDP). The dots on each curve represent working points when the false discovery rate level is set at the nominal 5% level. Results for all pairwise comparisons between the spike-in concentrations are shown. Source data are provided as a Source Data file.

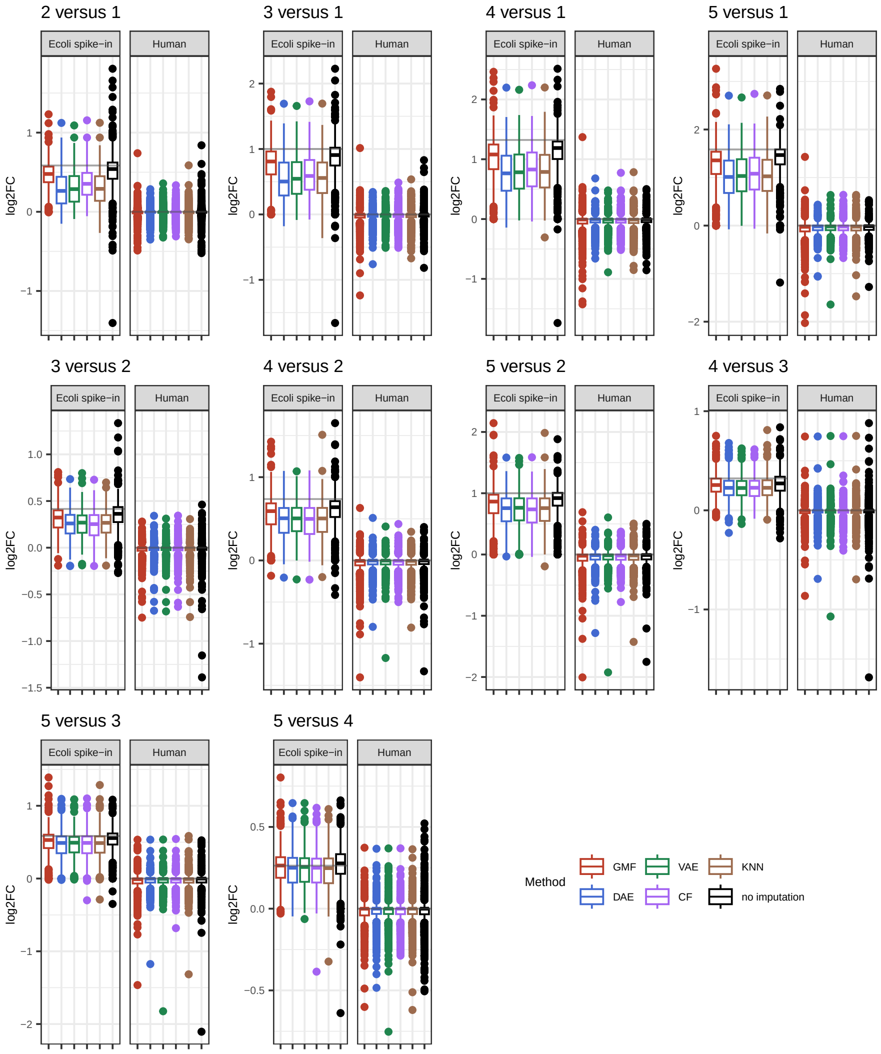

Supp. Fig. 41 Performance evaluation of differential abundance analyses using msqrob2 [6, 7] on the Shen dataset [8]. The *E. coli* proteins are mixed in different concentrations within a human background. Each plot shows the estimated log2 fold changes (FC) by msqrob2 for 756 *E. coli* spike in proteins, and 3954 reference human proteins. The boxplots are defined by the 25, 50, and 75% percentiles. The whiskers have a maximum length of 1.5 times the interquartile range. Source data are provided as a Source Data file.

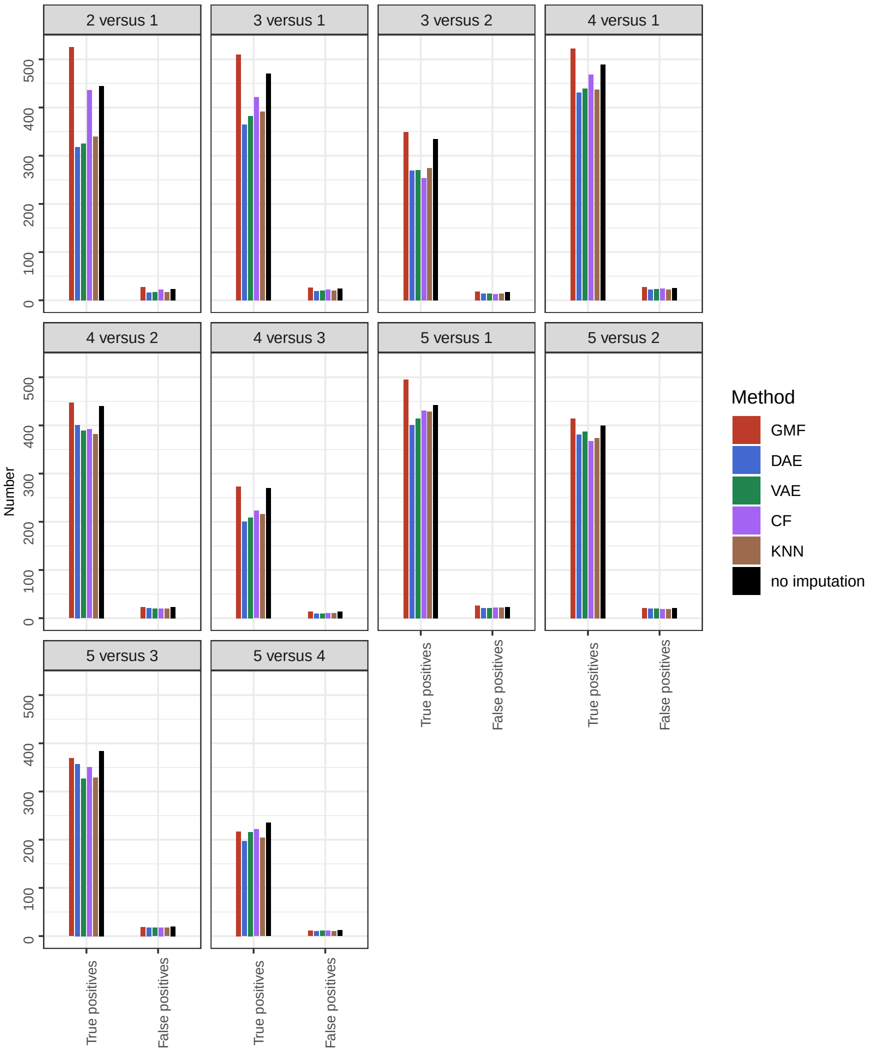

Supp. Fig. 42 Number of true positives and false positives retrieved in the Shen dataset [8] at a false discovery proportion of at most 5%. *E. coli* proteins are mixed in different concentrations and serve as true positives within a human background. Source data are provided as a Source Data file.

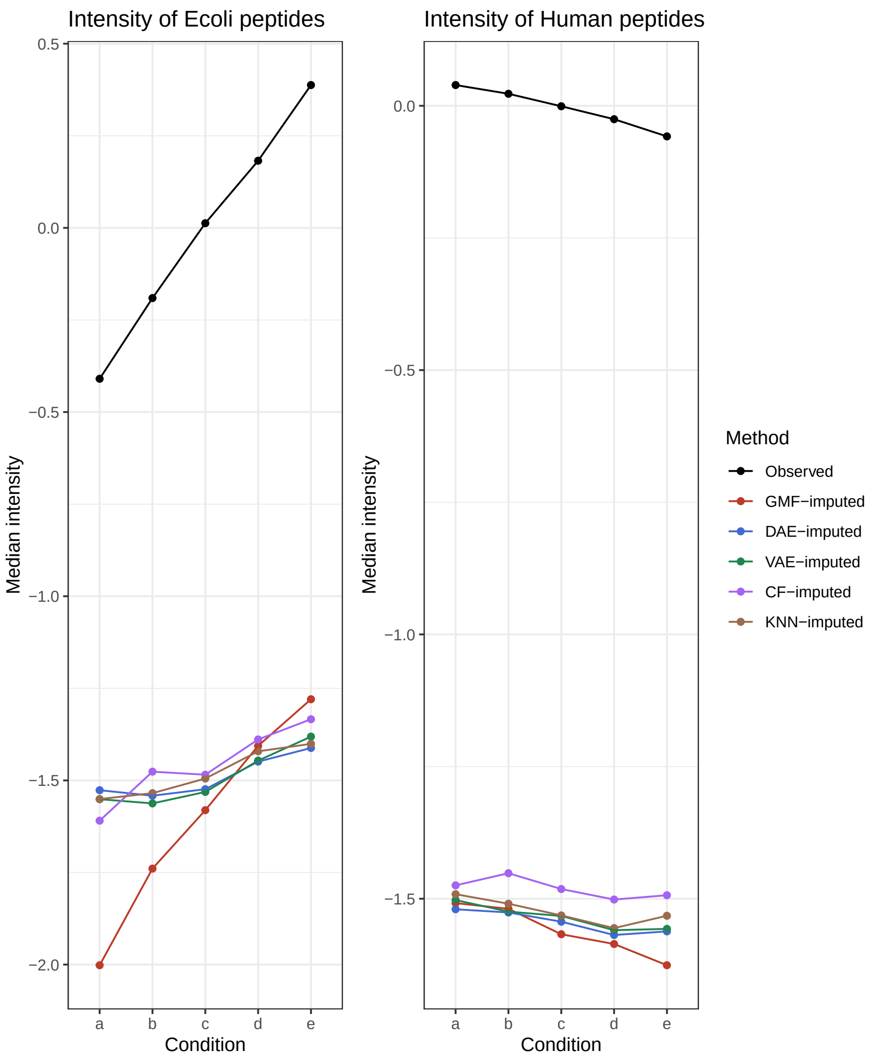

Supp. Fig. 43 Median peptide intensities of the observed values and values imputed with omicsGMF (GMF), DAE, VAE, CF and KNN-imputation in function of the spike-in condition in the Shen study [8]. The left panel shows data from *E. coli* spike-in proteins and the right panel from human background proteins (right). Source data are provided as a Source Data file.

Supplementary References

[1] Petrosius, V., Aragon-Fernandez, P., Üresin, N., Kovacs, G., Phlairaharn, T., Furtwängler, B., Op De Beeck, J., Skovbakke, S.L., Goletz, S., Thomsen, S.F., Keller, U.a.d., Natarajan, K.N., Porse, B.T., Schoof, E.M.: Exploration of cell state heterogeneity using single-cell proteomics through sensitivity-tailored data-independent acquisition. Nature Communications **14**(1), 5910 (2023) https://doi.org/10.1038/s41467-023-41602-1

[2] Leduc, A., Huffman, R.G., Cantlon, J., Khan, S., Slavov, N.: Exploring functional protein covariation across single cells using npop. Genome Biology **23**(1), 261 (2022) https://doi.org/10.1186/s13059-022-02817-5

[3] Paulovich, A.G., Billheimer, D., Ham, A.-J.L., Vega-Montoto, L., Rudnick, P.A., Tabb, D.L., Wang, P., Blackman, R.K., Bunk, D.M., Cardasis, H.L., Clauser, K.R., Kinsinger, C.R., Schilling, B., Tegeler, T.J., Variyath, A.M., Wang, M., Whiteaker, J.R., Zimmerman, L.J., Fenyo, D., Carr, S.A., Fisher, S.J., Gibson, B.W., Mesri, M., Neubert, T.A., Regnier, F.E., Rodriguez, H., Spiegelman, C., Stein, S.E., Tempst, P., Liebler, D.C.: Interlaboratory study characterizing a yeast performance standard for benchmarking lc-ms platform performance*. Molecular & Cellular Proteomics **9**(2), 242–254 (2010) https://doi.org/10.1074/mcp.M900222-MCP200

[4] Li, W., Yang, F., Wang, F., Rong, Y., Liu, L., Wu, B., Zhang, H., Yao, J.: scprotein: a versatile deep graph contrastive learning framework for single-cell proteomics embedding. Nature Methods **21**(4), 623–634 (2024) https://doi.org/10.1038/s41592-024-02214-9

[5] Lazar, C., Gatto, L., Ferro, M., Bruley, C., Burger, T.: Accounting for the multiple natures of missing values in label-free quantitative proteomics data sets to compare imputation strategies. Journal of Proteome Research **15**(4), 1116–1125 (2016) https://doi.org/10.1021/acs.jproteome.5b00981

[6] Goeminne, L.E., Gevaert, K., Clement, L.: Peptide-level robust ridge regression improves estimation, sensitivity, and specificity in data-dependent quantitative label-free shotgun proteomics*. Molecular & Cellular Proteomics **15**(2), 657–668 (2016) https://doi.org/10.1074/mcp.M115.055897

[7] Sticker, A., Goeminne, L., Martens, L., Clement, L.: Robust summarization and inference in proteome-wide label-free quantification. Molecular & Cellular Proteomics **19**(7), 1209–1219 (2020) https://doi.org/10.1074/mcp.ra119.001624

[8] Shen, X., Shen, S., Li, J., Hu, Q., Nie, L., Tu, C., Wang, X., Poulsen, D.J., Orsburn, B.C., Wang, J., Qu, J.: Ionstar enables high-precision, low-missing-data proteomics quantification in large biological cohorts. Proceedings of the National Academy of Sciences **115**(21), 4767–4776 (2018) https://doi.org/10.1073/pnas.1800541115 https://www.pnas.org/doi/pdf/10.1073/pnas.1800541115
