# Supplementary material for: omicsGMF: a multi-tool for dimensionality reduction, batch correction and imputation in bulk- and single-cell proteomics
Source: Nat Commun. 2026 May 20;17:6650. doi: 10.1038/s41467-026-73402-8 (PMC13381881; doi:10.1038/s41467-026-73402-8)
Supplement: Supplementary file 2 — Reporting Summary [file 41467_2026_73402_MOESM2_ESM.pdf]

nature portfolio

Corresponding author(s): Lieven Clement
Davide Risso

Last updated by author(s): Feb 19, 2025

Reporting Summary

Nature Portfolio wishes to improve the reproducibility of the work that we publish. This form provides structure for consistency and transparency in reporting. For further information on Nature Portfolio policies, see our Editorial Policies and the Editorial Policy Checklist.

Statistics

For all statistical analyses, confirm that the following items are present in the figure legend, table legend, main text, or Methods section.

n/a	Confirmed	
☐	☒	The exact sample size (*n*) for each experimental group/condition, given as a discrete number and unit of measurement
☒	☐	A statement on whether measurements were taken from distinct samples or whether the same sample was measured repeatedly
☒	☐	The statistical test(s) used AND whether they are one- or two-sided<br>*Only common tests should be described solely by name; describe more complex techniques in the Methods section.*
☒	☐	A description of all covariates tested
☒	☐	A description of any assumptions or corrections, such as tests of normality and adjustment for multiple comparisons
☐	☒	A full description of the statistical parameters including central tendency (e.g. means) or other basic estimates (e.g. regression coefficient) AND variation (e.g. standard deviation) or associated estimates of uncertainty (e.g. confidence intervals)
☒	☐	For null hypothesis testing, the test statistic (e.g. *F*, *t*, *r*) with confidence intervals, effect sizes, degrees of freedom and *P* value noted<br>*Give P values as exact values whenever suitable.*
☒	☐	For Bayesian analysis, information on the choice of priors and Markov chain Monte Carlo settings
☒	☐	For hierarchical and complex designs, identification of the appropriate level for tests and full reporting of outcomes
☒	☐	Estimates of effect sizes (e.g. Cohen's *d*, Pearson's *r*), indicating how they were calculated

Our web collection on statistics for biologists contains articles on many of the points above.

Software and code

Policy information about availability of computer code

Data collection	Code to reproduce the data collection and analysis is available through https://github.com/statOmics/GMFProteomicsPaper and https://doi.org/10.5281/zenodo.18651723.<br>Packages used for data collection are:<br>- scpdata package: Version 1.13.0 through Bioconductor 3.21.<br>- MaxQuant: version 1.5.2.8.
Data analysis	All code used to analyse the data is available through https://github.com/statOmics/GMFProteomicsPaper.<br>Also, the version of our developed package used in the manuscript is available in this repository. The newest update of our package is available through https://github.com/statOmics/omicsGMF.<br>Other packages used for data analysis:<br>- PIMMS package: https://github.com/RasmussenLab/pimms: downloaded from github at 10th of September 2024.<br>- SCPROTEIN package: https://github.com/TencentAILabHealthcare/scPROTEIN: downloaded from github at 10th of September 2024.<br>- imputeLCMD package: version 2.0 available through CRAN.<br>- sgdGMF package: Version 1.0 available through CRAN.<br>- impute package: Version 1.82.0 available through Bioconductor.<br>- prcomp from the stats package: Version 3.6.2 available through CRAN.<br>- msqrob2 package: Version 1.16 available through Bioconductor.<br>- scp package: Version 1.18 available through Bioconductor.<br>- scater package: Version 1.36 available through Bioconductor.

- NAguideR package: Version 0.2.1 available through github.
- nipals package: Version 1.0 available through CRAN.
- scpdata package: Version 1.18.0 available through Bioconductor.
- scp package: Version 1.20 available through Bioconductor.

For manuscripts utilizing custom algorithms or software that are central to the research but not yet described in published literature, software must be made available to editors and reviewers. We strongly encourage code deposition in a community repository (e.g. GitHub). See the Nature Portfolio guidelines for submitting code & software for further information.

Data

Policy information about availability of data

All manuscripts must include a data availability statement. This statement should provide the following information, where applicable:

- Accession codes, unique identifiers, or web links for publicly available datasets
- A description of any restrictions on data availability
- For clinical datasets or third party data, please ensure that the statement adheres to our policy

The processed Petrosius (accession number: MSV000092429 [https://doi.org/10.25345/C5DB7W12H]) and Leduc (accession number: MSV000089159 [doi:10.25345/C5W950S0W]) data are available through the scpdata package from Bioconductor, or at https://github.com/statOmics/GMFProteomicsPaper and Zenodo at https://doi.org/10.5281/zenodo.18651723. The unfiltered intensities of the CPTAC and Shen data (identifier: PXD003881 [https://dx.doi.org/10.6019/PXD003881]) are available at https://github.com/statOmics/GMFProteomicsPaper and through Zenodo at https://doi.org/10.5281/zenodo.18651723. Supplementary Information and Source data are provided with this paper.

Research involving human participants, their data, or biological material

Policy information about studies with human participants or human data. See also policy information about sex, gender (identity/presentation), and sexual orientation and race, ethnicity and racism.

Reporting on sex and gender	We used publicly available data on cell lines and no data of human participants were used.
Reporting on race, ethnicity, or other socially relevant groupings	We used publicly available data on cell lines and no data of human participants were used.
Population characteristics	We used publicly available data on cell lines and no data of human participants were used.
Recruitment	We used publicly available data on cell lines and no data of human participants were used.
Ethics oversight	We used publicly available data on cell lines and no data of human participants were used.

Note that full information on the approval of the study protocol must also be provided in the manuscript.

Field-specific reporting

Please select the one below that is the best fit for your research. If you are not sure, read the appropriate sections before making your selection.

[x] Life sciences [ ] Behavioural & social sciences [ ] Ecological, evolutionary & environmental sciences

For a reference copy of the document with all sections, see nature.com/documents/nr-reporting-summary-flat.pdf

Life sciences study design

All studies must disclose on these points even when the disclosure is negative.

Sample size	No sample-size calculation was performed. This is not relevant for our study as we did not collect data ourselves. We chose the number of datasets to reflect a wide range of different technologies (label-free versus TMT-labeled, as well as bulk versus single-cell data).
Data exclusions	Data was excluded during the data processing using common thresholds from the RNA-sequencing and proteomics literature. These threshold were set prior to the data analysis.
Replication	For the simulated results, multiple simulation scenarios were used, and are all described in the manuscript. All analyses are fully reproducible via open-source code.
Randomization	This is not relevant for our study as we did not collect data ourselves.
Blinding	This is not relevant for our study as we did not collect data ourselves.

Reporting for specific materials, systems and methods

We require information from authors about some types of materials, experimental systems and methods used in many studies. Here, indicate whether each material, system or method listed is relevant to your study. If you are not sure if a list item applies to your research, read the appropriate section before selecting a response.

Materials & experimental systems

n/a	Involved in the study
☒	☐ Antibodies
☒	☐ Eukaryotic cell lines
☒	☐ Palaeontology and archaeology
☒	☐ Animals and other organisms
☒	☐ Clinical data
☒	☐ Dual use research of concern
☒	☐ Plants

Methods

n/a	Involved in the study
☒	☐ ChIP-seq
☒	☐ Flow cytometry
☒	☐ MRI-based neuroimaging

Plants

Seed stocks	We used publicly available data on cell lines and no plant data were used.
Novel plant genotypes	We used publicly available data on cell lines and no plant data were used.
Authentication	We used publicly available data on cell lines and no plant data were used.
