# Supplementary material for: omicsGMF: a multi-tool for dimensionality reduction, batch correction and imputation in bulk- and single-cell proteomics
Source: Nat Commun. 2026 May 20;17:6650. doi: 10.1038/s41467-026-73402-8 (PMC13381881; doi:10.1038/s41467-026-73402-8)
Supplement: Supplementary file 3 — Transparent Peer Review file [file 41467_2026_73402_MOESM3_ESM.pdf]

nature portfolio

Peer Review File

omicsGMF: a multi-tool for dimensionality reduction, batch correction and imputation in bulk- and single-cell proteomics

Corresponding Author: Professor Lieven Clement

This file contains all reviewer reports in order by version, followed by all author rebuttals in order by version.

Version 0:

Reviewer comments:

Reviewer #1

(Remarks to the Author)
Segers et al present omicsGMF, a generalized matrix factorization (GMF) framework for dimensionality reduction, batch correction, and imputation in mass spectrometry-based proteomics. omicsGMF integrates multiple preprocessing steps within a single framework, contrasting with conventional workflows that rely on separate steps and tools.
The manuscript is thorough and technically detailed. omicsGMF is benchmarked across bulk and single-cell proteomics datasets, the authors demonstrate improvements in data visualization, imputation accuracy, and differential abundance analysis, with comparisons to state-of-the-art imputation methods and existing workflows. Overall the work presented is quite strong with extensive benchmarking, I only have a few comments for the authors:

The authors claim scalability for large single-cell datasets, yet quantitative runtime benchmarks (e.g., time vs. number of cells or features) are not provided. Adding runtime/performance plots for omicsGMF and comparable methods would bolster these claims.
The manuscript currently does not explicitly discuss the general assumptions omicsGMF makes about the missingness mechanism (e.g., MAR vs. MNAR). It would be helpful to clarify whether omicsGMF implicitly assumes missingness is Missing At Random (MAR) i.e., explained by covariates and latent factors.
The manuscript mentions scPROTEIN was excluded because it failed to generate sensible embeddings, citing documentation limitations. Given that scPROTEIN is a unified framework (like omicsGMF) and is specifically designed for single-cell proteomics, it would be helpful to clarify whether additional troubleshooting (e.g., contacting authors, adjusting hyperparameters, testing smaller subsets) was attempted.
While Figure 4 effectively shows how the out-of-sample deviance depends on the number of latent factors, it would be helpful to also include practical guidance for users. For example, how sensitive downstream biological results (like differential abundance) are to the choice of d.

(Remarks on code availability)
authors provide documentation to reproduce their analyses on github. The tool is also available on github.

Reviewer #2

(Remarks to the Author)
In this manuscript, the authors present omicsGMF, a method designed for both bulk and single-cell proteomics data, which integrates imputation, batch correction and principal component analysis into a unified framework to facilitate dimensionality reduction. While the integration of these steps is practically useful, the proposed model does not exhibit substantial methodological innovation. Moreover, the experimental results do not sufficiently demonstrate the method’s potential to contribute to novel biological insights. I have the following comments:

1. omicsGMF models intensities as a function of known sample-level covariates, feature-level covariates, latent factors and their loadings. However, in the experiments presented in this manuscript, only a subset incorporated sample-level covariates, and feature-level covariates were not utilized in any of the reported analyses. In the absence of both sample-level and feature-level covariates, the model essentially reduces to a form of principal component analysis. As such, the core methodological innovation of the model is not clearly articulated, and the rationale behind its expected effectiveness remains

unclear.

2. At the beginning of parameter initialization, missing values are imputed using μ_0. However, the rationale behind the definition of μ_0 is not clearly explained. It also remains unclear whether the choice of μ_0 has a significant impact on the final results.

3. In the Introduction, the authors categorize missingness into two types: missing completely at random (MCAR) and missing not at random (MNAR). They point out that these two forms of missingness require distinct imputation strategies, and that the application of methods tailored to only one type can significantly distort the distribution of protein-level intensities. While omicsGMF is claimed to address both MCAR and MNAR simultaneously, the underlying mechanism by which it achieves this is not clearly described. Further clarification is needed to understand how the model differentiates and appropriately handles these two fundamentally different types of missingness.

4. The authors propose several approaches for selecting the number of latent factors, including examining the eigenvalues in a scree plot, using information criteria such as AIC or BIC, and applying cross-validation. Do these methods yield consistent estimates of the number of latent factors? How sensitive are the final results to the choice of selection method?

5. The authors repeatedly characterize omicsGMF as a fast method. However, the manuscript lacks any quantitative analysis, benchmarking, or discussion of runtime performance.

6. In several experiments, the incorporation of known covariate information appears to result in reduced performance. For example, this is observed in the imputation task on the label-free CPTAC dataset, as shown in Supplementary Fig. 16. How can this phenomenon be explained? Does it indicate a limitation in the method's design or implementation regarding the integration of covariates?

7. In the imputation task on the TMT-labeled Leduc dataset, omicsGMF outperformed the other methods when accounting for batch effects. However, when batch effects were not considered, omicsGMF showed no clear advantage. This raises an important question: how would the performance of the other methods change if batch effects were adjusted prior to imputation, or if imputation were performed within each batch separately?

8. In the Dimensionality reduction and visualization of proteomics data section, the assessment is primarily based on qualitative observations. It is recommended to include quantitative evaluations to provide a more objective and comprehensive comparison.

9. To more convincingly establish the practical utility of the proposed method, additional and more extensive experiments are needed to evaluate whether it can yield more informative or biologically meaningful findings in downstream analyses compared to existing methods.

(Remarks on code availability)
Although the authors have provided the code, I recommend including more detailed documentation, such as example notebooks, to guide users through reproducing the experiments. This would significantly enhance the usability of the code and ensure that the results presented in the paper can be easily reproduced by the community.

Reviewer #3

(Remarks to the Author)
This manuscript describes the omicsGMF tool, which integrates batch effect correction, imputation, and dimensionality reduction for processing bulk- and single-cell proteomic datasets. The concept of addressing sample, feature, and unknown covariates within a unified regression framework is an interesting and common approach to omics data processing.
After careful review, I regret to recommend rejection of the manuscript due to several critical concerns regarding its novelty, methodological rigor, and alignment with the priorities of proteomic research. Below, I outline these concerns to provide clarity and constructive feedback for future work.
Major concerns:
1. Limited Novelty: Covariate correction is a common practice in omics data analysis, and the omicsGMF tool does not sufficiently distinguish itself from existing methodologies. While the inclusion of an "unknown covariate" and iterative imputation is noted, these features do not represent a sufficiently novel contribution to meet the journal's scope and standards. A more robust justification of the tool's unique advancements, supported by comprehensive comparisons with state-of-the-art methods, is necessary to establish its novelty.
2. Unfair Methodological Comparisons: The evaluation of omicsGMF against other methods lacks fairness and rigor. It is widely recognized that imputation alone is inadequate for batch effect correction in cell clustering, as normalization is a prerequisite step. However, the manuscript compares omicsGMF, which incorporates normalization, to a pipeline of imputation and PCA without applying normalization to the competing methods. This discrepancy invalidates the reported performance improvements. Furthermore, the three datasets used in the study have been analyzed in prior work, which demonstrated effective cell clustering and identification of differentially expressed proteins (DEPs) with biological significance. The absence of DEP analysis in this study represents a critical oversight, undermining the tool's utility.
3. Limited Relevance to Proteomic Analysis Goals: Proteomic studies primarily aim to identify biologically significant

biomolecules for diagnostic or therapeutic applications or dissecting molecular basis. The manuscript's focus on cell and sample clustering, without exploring actionable biomolecular insights, limits its relevance to the proteomics field. Incorporating a comparative analysis of DEPs identified by omicsGMF against those reported in prior studies would be essential to demonstrate the tool's practical value.
Minor concerns:
1. Lack of Statistical Significance in Comparisons: The manuscript does not provide clear indicators of statistical significance, such as the distribution of mean absolute error (MAE) for imputed values across methods. Including statistical tests (e.g., t-tests or ANOVA) to compare omicsGMF's performance with other methods would strengthen the analysis.
2. Handling of Leduc Dataset Issues: Previous work has reported low identification and quantification rates in certain batches of the Leduc dataset. The manuscript does not clarify whether these problematic batches were excluded or if consistent effects were observed across all batches. Addressing this would enhance the robustness of the analysis.
3. Need for Benchmark Datasets: To validate the applicability of omicsGMF, the authors should consider using well-characterized datasets with known protein expression ratios across species. Such datasets are widely available and would provide a stronger basis for evaluating the tool's performance.

In conclusion, while the omicsGMF tool shows potential, the lack of demonstrated novelty, methodological flaws in comparative analyses, and limited alignment with proteomic research priorities preclude its acceptance in its current form. I encourage the authors to address these concerns by clarifying the tool's unique contributions, conducting fair comparisons with normalized baselines, and incorporating analyses of biologically relevant outcomes, such as DEP identification. These revisions could significantly strengthen the manuscript for future submission.

(Remarks on code availability)

Reviewer #4

(Remarks to the Author)
The article title 'omicsGMF: a multi-tool for dimensionality reduction, batch correction and imputation applied to bulk- and single cell proteomics data' was an interesting read presenting valuable ideas. In general, I am confident the article could pass the revision process, but - maybe unsurprisingly - some clarification and/or corrections might still be needed.

More detailed comments per section, subsection or figure below.

Introduction
L58-L61: I guess both sentences will be w.r.t to the publication in [7]?
L81: 'primarily designed for label-free bulk proteomics with data-dependent acquisition': As I see it most imputation methods are applicable to most matrices. The focus is maybe less in design, but in relevance. TMT without batches has close to zero missing values and data-dependent has a higher share than data-independent. Nonetheless it is good to also consider other setups in benchmarking. So potentially rephrase a bit or go into detail about the particularities of multi-batch TMT setups w.r.t to missing values.

L90-L91: On could argue that good tools would create higher-level functions connecting different analysis steps (e.g. imputation and batch correction). Your method makes this unnecessary. In this paragraph you already discuss results which I find too early. Mentioning it in the introduction also leads to having Figure1 to be the PCAs, which is unfortunate as Figure2 rather introduces omicsGMF (and would be the first one to be shown on any social media link).
L95: I remember to have seen scVI. It lacks the interpretability of omicsGMF but I thought it does aim for similar things. You could look at the tooling around it to see if this is relevant (scvi-tools). I am not using it nor am I a developer of it. If you know it, I would rather be interested as an user if this is something you considered and discarded – or if it goes into the same direction. If irrelevant, ignore this hint.
https://docs.scvi-tools.org/en/stable/user_guide/index.html

Results
I like the SVD-like approach including factors of known covariance resulting in a linear (in parameters) and interpretable method.

Figure2: 'feature-specific effect': is this correct? As B is sample batch-effects. Assuming it is correct, a bit more explanations would help me. Is it p sample specific covariates (batch-effects) and q feature-specific covariates (not-sure what this normally is called). Then there is 'cell-by-peptide' and 'cell-specific'. I would suggest to keep the overview a bit more generic as in principle it could be anything (with normally distributed featues?)
If you have an example of feature-specific covariates from any of the three datasets included into the paper, this would be great (probably you have it somewhere, but at the time of writing this review I do not recall it)

L146: you mention iterative and later (L154) multi-step. At first this confused me, but one is w.r.t to the fitting algorithm (iterative regression) and the other more conceptual. I would argue that in L154 the reader does not yet know what 'multi-step' workflows are. I am not sure how to solve this, but reading your method I would say that it is imputation+linear batch correction with different tools and scProtein (which you are not able to use in the end). Could you maybe refer to the methods or a table here to make it clearer? I would maybe just swap the sentences (starting in L154 and L155): your method can do both and avoids higher-level workflows combining two things.

L157-L160: Is the workflow not rather a set of linear equations. Gaussian like-likelihood can be optimized by a variational autoencoder loss-function. The strong point is that is similar to PCA and thus interpretable?

L174: in [19]?

L175-L187: Is this not rather for the last part of the introduction where you outline you detailed plan (or part of the section summarizing the following sections?)

Dimensionality reduction and visualization of proteomics data

In this section the other multi-step workflows (L200-L206) are defined (by the authors). I would mainly suggest that the comparison in Supplementary Fig. 6 are better suited for comparison to omicsGMF. If lucky imputation, batch correction and PCA are three function calls (probably this is over optimistic). For me adding batch correction to the comparison says that omcisGMF is a good method as it makes it easier to handle all the aspects without knowing several tools .

L245: Here PSMs are mentioned, in Figure2 and elsewhere peptides are mentioned. This just makes we wonder what kind of data was used? Were PSMs, precursors (sequence, PTMs and charge, potentially summarizing several PSMs) or peptides (sequence plus PTMs) used?

L257: The competing workflows are defined by the authors, if I get this correctly. So I would rather say 'other workflows'. Same in L277 'existing methods': There are not really existing methods which do this in one step?

L264: This now goes to a new figure, which might warrant a new section. I would maybe explain which matrix dimension this refers to in Figure2 and state the matrix symbol. Then Figure4 is also easier to understand.

Figure4: I would mention the matrix which dimensions are optimized. Are all mentionend covariates batch-effects? And I would like to know what the values are (could be elsewhere, but I guess peptides or PSMs?)

L298-L304: A linear model captures a linear increase in concentrations. That is good, but maybe also not the most challenging experimental setup in that case. I am not sure what the discussion on Figure4, Panel C tells me for using it in practice. Is it bad? Will I see batch effects in my plots?
Imputation of missing values
So just to be sure. The error plots are w.r.t to the log2 and mean standardised intensities (to zero mean)? I normally would expect nonnegative values in Figure5.
The paragraph does not contain the results in numbers which makes it a bit hard to compare and assess. Was there a specific reason for that? I would argue that if you say a method is better, then this should be quantified a tiny bit.

L314-L322: Maybe refer to the methods? The comparison is w.r.t to the default parameters of the other methods (as stated in the methods)?

L343-L347. Could you elaborate how the ground trough in differential analysis is related to the imputation comparisons? You state that you simulated missing values, which you use as ground truth in any comparison, or do I misunderstand that? The median of all imputed peptides is used to illustrate the concentration ratio?

L350-L354: GMF-imputation seems to have some out-of-distribution predictions which can also lead to artefacts in differential analysis (Figure5, values way below -5). Is there any explanation why this has no effect on the differential analysis? Then, aren't these simulated missing values which are used for this comparison? If it is non-simulated missing values, then this comparison is still not yet showing ground-truth I would argue. You compare between 5 conditions one PSM or peptide. If in one condition some values are missing you want to see it correctly imputed? The overall distribution can be a proxy, but in the end you would need to show maybe the ratio's distribution using different imputation methods? (I would need to think more about it).

Differential analysis

Here you start talking about human proteins, whereas before the analysis was on PSM or peptides level (see question). Did you continue your analysis on the peptide level (which is probably the most sensible)? How many peptides of the 48 spike-in proteins did you observe? So what is 'original non-imputed data'?

I did not find the a method section for the details on this comparison, which would help me understand the mathematical setup here (simple t-test with multiple-testing correction on 'corrected' data?)

L379-L389: Same as before. Quantitative comparison without any numbers. It's w.r.t to AUC values?
Figure5: In panel A (B versus A) the KNN and DAE curves look off. Is it due to an artefact, an error or is there a performance reason? Is FDP the same as FDR as you mention both. (I looked it up: In theory this might be different, but here it is the same due to the empirical nature?)
Figure6: So no-imputation is best, but accounting for the ionization makes omicsGMF better? So no imputation and no batch correction leads already to the best results? How many missing values were present in the peptides associated with the 48

human proteins?

In general for results:
It would be nice to have some number in the text to not go estimate these from the figures. This also helps me at least double check if the text and the figures align (kind of a double check). Do you support Supplementary Data for all the Figures (at some point I guess you will need to anyways)?

In general the results have a lot of interesting ideas and I have to admit that I did not check all supplementary figures.

Discussion

Brief and neat. Maybe add your SGD mini-batch approach. Similar to neural networks, but with a clearer linear parameter setup. So you gain computational efficiency without compromising on interpretability.

Methods

L497: ‘can contain’ is there a default? I guess the user does not need to care about that?
Data

L658-L659. So you mention peptides for yeast, but not for humans. Is there a reason? So it is still not clear how you get from peptides to proteins (or you do not) for the differential analysis. In the end I would expect to perform the DA analysis on peptides as the visualizations are based on peptides, right?

(Remarks on code availability)
Well structured repository, although not executed version of all the files exist. The makefile should allow to re-run it fairly easy for the authors and it gives hints on the structure.

I have not seen how to set up the dependencies myself. A docker image could help or some details instructions. But I would only invest the time if the article goes further and their answers are convincing.

Version 1:

Reviewer comments:

Reviewer #1

(Remarks to the Author)
The authors have sufficiently addressed all my comments, I wish them best of luck with the remaining publication process.

(Remarks on code availability)
Both the code for reproducing results from the paper and the package omicsGMF are available on github and well documented.

Reviewer #2

(Remarks to the Author)
The authors have addressed most of my previous concerns, and the manuscript has been substantially improved. I only have a few remaining suggestions for further clarification and presentation improvement:

1. In the revised manuscript, the authors have added assumptions regarding how their method handles two types of missing data, which is appreciated. However, the current description is still rather abstract and may not be sufficiently intuitive for readers to follow. In addition, the concept of Missing At Random (MAR) is newly introduced but not explicitly explained, which further affects the clarity of this part. I suggest that the authors revise this part to more clearly and concretely explain what assumptions are made for each type of missingness and how the proposed method handles each case.

2. In Supplementary Figs. 35 and 36, the colors are difficult to distinguish. The figures should be revised to improve readability.

(Remarks on code availability)

Reviewer #3

(Remarks to the Author)
I thank the authors for their extensive point-by-point responses and for the inclusion of new quantitative benchmarks, such as the runtime analysis and Average Silhouette Width (ASW). While the technical quality of the work is evident and omicsGMF represents a robust addition to the Bioconductor ecosystem , my primary concerns regarding conceptual novelty and the lack

of transformative biological insight remain. While the integration of preprocessing steps is practically useful, the model does not exhibit the level of methodological innovation or novel biological discovery expected for this venue. I have the following comments:
1. The central claim of the manuscript is the "unified" nature of the omicsGMF framework, which simultaneously addresses batch correction, missing data, and dimensionality reduction. While this integration is practically useful for avoiding the "arbitrary choice of the sequence" in multi-step workflows, the underlying methodological novelty is limited.
(a) Covariate correction and matrix factorization are established practices in other 'omics fields, and omicsGMF largely translates these concepts to proteomics.
(b) The framework reduces to a form of expectation-maximization PCA (EM-PCA) when covariates are omitted, which the authors acknowledge.
(c) The primary innovation—using an efficient adaptive stochastic gradient descent (SGD) algorithm to unlock these models for large datasets—is a significant engineering optimization but represents an incremental statistical advance rather than a breakthrough in computational biology.

2. I appreciate the addition of Supplementary Figures 7 and 8 comparing omicsGMF to sequential workflows. On the Leduc dataset, the authors admit that ASW results are "similar between the most efficient methods" when comparing omicsGMF to workflows that perform batch correction and imputation sequentially. While omicsGMF provides a more user-friendly, single-step approach, the data suggest that a well-constructed traditional pipeline can achieve comparable biological separation.

3. A key requirement for Nature Communications is that a new tool must enable a biological insight that was previously unattainable. The authors demonstrate increased sensitivity in detecting differentially abundant (DA) proteins in spike-in benchmarks like CPTAC and Shen et al. However, these results rely on ground-truth datasets where the biology is already known. The manuscript still lacks a "discovery" component where omicsGMF identifies a novel, actionable biomolecular insight in a complex biological system that existing state-of-the-art tools could not resolve.

4. The authors clarify that their joint modeling approach reduces what would be Missing Not At Random (MNAR) in a univariate context to Missing At Random (MAR) conditional on latent factors. While this is a sensible statistical treatment, it does not fundamentally resolve the biological challenge of proteins missing due to low abundance—a "limit of detection" issue that remains a major hurdle in the field.

The authors have significantly improved the technical depth of the manuscript. However, omicsGMF remains a technical refinement of existing matrix factorization techniques. Given the lack of a primary biological discovery and the incremental nature of the "unified" framework, I maintain my initial assessment that the work does not meet the impact threshold for Nature Communications.

(Remarks on code availability)

Reviewer #4

(Remarks to the Author)
I checked the answers to my previous remarks and re-read most of the article. I am overall satisfied and thanks the authors for the in-detail revision, making it easy to follow-up half a year later.

Figure1: ‘Issues with conventional multi-step workflows..’ The figures are showing imputation plus PCA. I would say this is an imputation-only workflow. It was confusing to me and I only remembered later that you want to address both imputation and batch-correction in one go.

OmicsGMF needs batch-metadata to work (to perform batch correction). It is clear to me that you need these information, but maybe you want to highlight the required inputs, especially as feature-specific variables are not used in the study. I see that the PCA will be an output no-matter what, but this is not the focus of the study in my opinion.

(Remarks on code availability)
the paper analysis is in one repo and the core is on bioconductor. bioconductor has high standards, so i hope it will be good code.

Open Access This Peer Review File is licensed under a Creative Commons Attribution 4.0 International License, which permits use, sharing, adaptation, distribution and reproduction in any medium or format, as long as you give appropriate credit to the original author(s) and the source, provide a link to the Creative Commons license, and indicate if changes were made.
In cases where reviewers are anonymous, credit should be given to 'Anonymous Referee' and the source.
The images or other third party material in this Peer Review File are included in the article's Creative Commons license, unless indicated otherwise in a credit line to the material. If material is not included in the article's Creative Commons license and your intended use is not permitted by statutory regulation or exceeds the permitted use, you will need to obtain permission directly from the copyright holder.
To view a copy of this license, visit https://creativecommons.org/licenses/by/4.0/

Point-by-point responses to the reviewers' comments

Reviewer #1

"Segers et al present omicsGMF, a generalized matrix factorization (GMF) framework for dimensionality reduction, batch correction, and imputation in mass spectrometry-based proteomics. omicsGMF integrates multiple preprocessing steps within a single framework, contrasting with conventional workflows that rely on separate steps and tools. The manuscript is thorough and technically detailed. omicsGMF is benchmarked across bulk and single-cell proteomics datasets, the authors demonstrate improvements in data visualization, imputation accuracy, and differential abundance analysis, with comparisons to state-of-the-art imputation methods and existing workflows. Overall the work presented is quite strong with extensive benchmarking, I only have a few comments for the authors":

Reviewer 1 - Comment 1

"The authors claim scalability for large single-cell datasets, yet quantitative runtime benchmarks (e.g., time vs. number of cells or features) are not provided. Adding runtime/performance plots for omicsGMF and comparable methods would bolster these claims."

Our response: We thank the Reviewer for their thoughtful comments, which we addressed by including an additional supplementary figure in our revised manuscript with the following accompanying text in the paragraph 7 of section "Dimensionality reduction and visualization of proteomics data":

Note further that omicsGMF reduces to a kind of expectation-maximization (EM) PCA when no sample or feature-level covariates are included. However, conventional EM-PCA struggles to converge, probably due to the excessive number of missing values (Supplementary Fig. 13). Furthermore, EM-PCA is very slow, and does not scale well

to larger datasets. Neither does NIPALS, which performs PCA by ignoring missing values. In contrast, omicsGMF solves these problems by optimizing its parameters through an efficient adaptive stochastic gradient descent algorithm. This makes omicsGMF fast and scalable to large datasets, especially with regards to PCA-like algorithms EM-PCA and NIPALS, and to imputation methods such as DAE, VAE and CF (Supplementary Fig. 14).

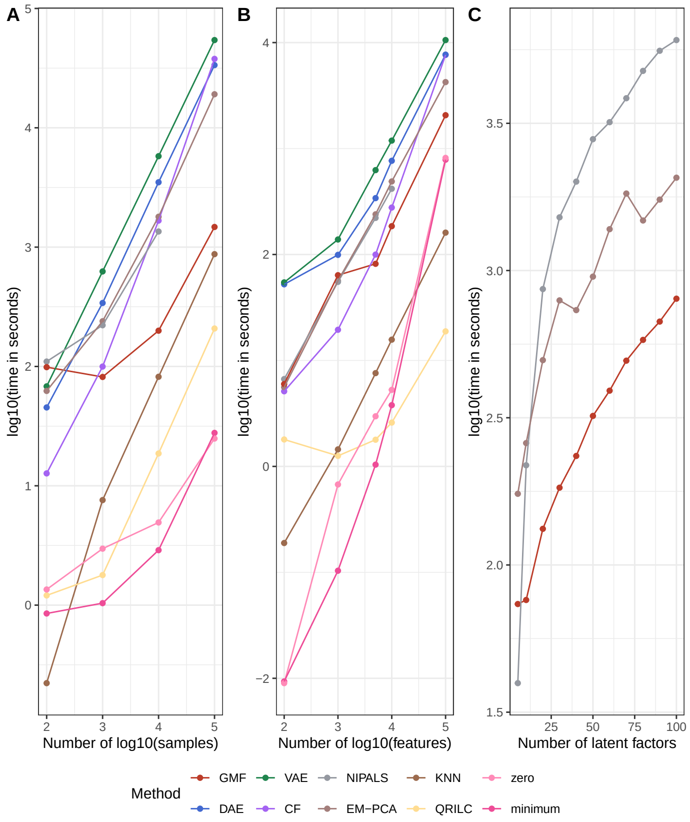

Supp. Fig. 14 Benchmark of computational time (in log10 of the number of seconds) in function of the number of samples (Panel A), the number of features (Panel B) and the number of latent factors for PCA-like algorithms (Panel C). Thousand samples are used in Panel B and C, five thousand features are used in Panel A and C, and ten latent factors are used in Panel A and B. omicsGMF is much faster compared to PCA-like algorithms that can deal with missing values (NIPALS and EM-PCA), and is faster than PIMMS imputation (DAE, VAE and CF) on large datasets. Methods like KNN, QRILC, zero and minimum imputation are faster than omicsGMF, but do not account for the correlation structure in the data. NIPALS could not return results in Panel A for 100000 samples.

Reviewer 1 - Comment 2

"The manuscript currently does not explicitly discuss the general assumptions omicsGMF makes about the missingness mechanism (e.g., MAR vs. MNAR). It would be

helpful to clarify whether omicsGMF implicitly assumes missingness is Missing At Random (MAR) i.e., explained by covariates and latent factors."

Our response: We thank the reviewer for their suggestion. We have now explicitly included the imputation assumptions of omicsGMF in the Results and Method section.

The second paragraph of the Results now reads as follows:

The advantages are threefold. First, the imputed values account for known sample- and feature-level covariates, as well as the latent structure, which implies imputation under a Missingness At Random (MAR) assumption conditional on both known covariates and information in other features through latent factors. ~~resulting~~ This results in accurate imputed intensities even when dealing with significant technical effects.

We adjusted the first paragraph of section "Imputation of missing values" in the revised manuscript:

The final mean estimates obtained from omicsGMF can also be used for the imputation of missing values while accounting for latent factors, as well as for known sample- and feature-level covariates. This imputation strategy assumes missingness at random that can be explained by known covariates and latent structure. Although it does not explicitly assume missingness due to low abundance, which is particularly present when considering data from a single feature (e.g. a precursor or peptide) in isolation, omicsGMF leverages the known covariates and the correlation structure across multiple precursors, peptides and proteins. This joint modelling of features allows to partially explain missingness by low abundance in one specific feature using the information

in known covariates and in other features through latent factors, effectively reducing what would be considered MNAR in a univariate context to MAR, conditional on the observed covariates and latent factors. In cases where neither covariates or latent factors contain relevant information, the imputation strategy reduces to a kind of MCAR strategy. To evaluate imputation performance, we simulate missingness both completely at random (MCAR) and missing not at random (MNAR) due to low abundance (see Methods). The mean absolute error (MAE) between the imputed and original values is then assessed across ten different random seeds, considering scenarios where 25%, 50%, and 75% of the masked values are attributed to low abundance. The MAEs for all simulations are available in Supplementary Data.

The subsection 'Optimization of the parameters' in the Methods is adapted as follows:

At convergence, the estimated means, $\hat{\boldsymbol{\mu}}_t$, can be used for imputation purposes, which assumes missing at random conditional on the known covariates and latent structure.

Reviewer 1 - Comment 3

"The manuscript mentions scPROTEIN was excluded because it failed to generate sensible embeddings, citing documentation limitations. Given that scPROTEIN is a unified framework (like omicsGMF) and is specifically designed for single-cell proteomics, it would be helpful to clarify whether additional troubleshooting (e.g., contacting authors, adjusting hyperparameters, testing smaller subsets) was attempted."

Our response: For the first version of the manuscript we did not provide additional troubleshooting as the same error was already reported in a github issue on April

12 2024: https://github.com/TencentAILabHealthcare/scPROTEIN/issues/5, which remained unanswered by the authors. Moreover, the github repository did not undergo changes since publication. We have now confirmed this issue on their github repository, and contacted the authors by email, to which we received no response.

Reviewer 1 - Comment 4

"While Figure 4 effectively shows how the out-of-sample deviance depends on the number of latent factors, it would be helpful to also include practical guidance for users. For example, how sensitive downstream biological results (like differential abundance) are to the choice of d."

Our response: We thank the reviewer for their suggestion. We have now included performance curves for the CPTAC case study where we assess differential abundance for a grid of different numbers of latent variables d. This clearly shows that arbitrarily choosing the latent variables, as in conventional methods, results in suboptimal performance compared to the performance obtained using our implemented cross-validation. We illustrate this in our revised manuscript with two additional supplementary figures, to which we refer in paragraph 4 of section "Differential analysis":

Furthermore, Supplementary Figs. 35 and 36 assess the quality of the downstream differential abundance analysis across a range of different numbers of latent factors. Interestingly, it clearly shows that superior performance is obtained with an omicsGMF model that uses the number of latent factors suggested by cross-validation. This confirms that carefully selecting the number of latent factors is a valuable feature of omicsGMF, which can be important for subsequent downstream analyses.

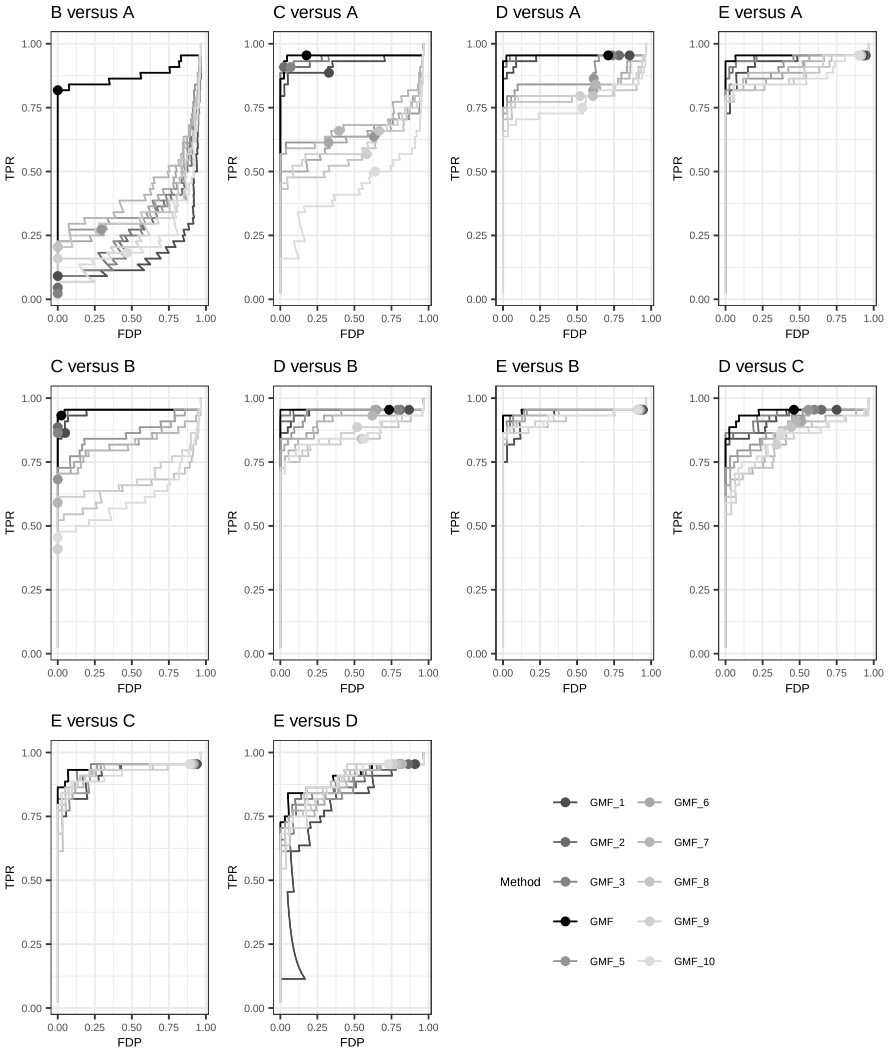

Supp. Fig. 35: Performance evaluation of differential abundance analyses using msqrob2 [25, 26] on the CPTAC dataset [16], for different numbers of latent factors used in omicsGMF. omicsGMF's cross-validation suggested four latent factors. Data from Lab 1, which suffers from ionization issues, are excluded. Human UPS proteins are differentially spiked between conditions while yeast background proteins serve as true negative control. Each curve shows true positive rate (TPR) in function of false discovery proportion (FDP). Dots on each curve represent working points for FDR level at nominal 5% level. Results for all pairwise comparisons between spike-in concentrations are shown.

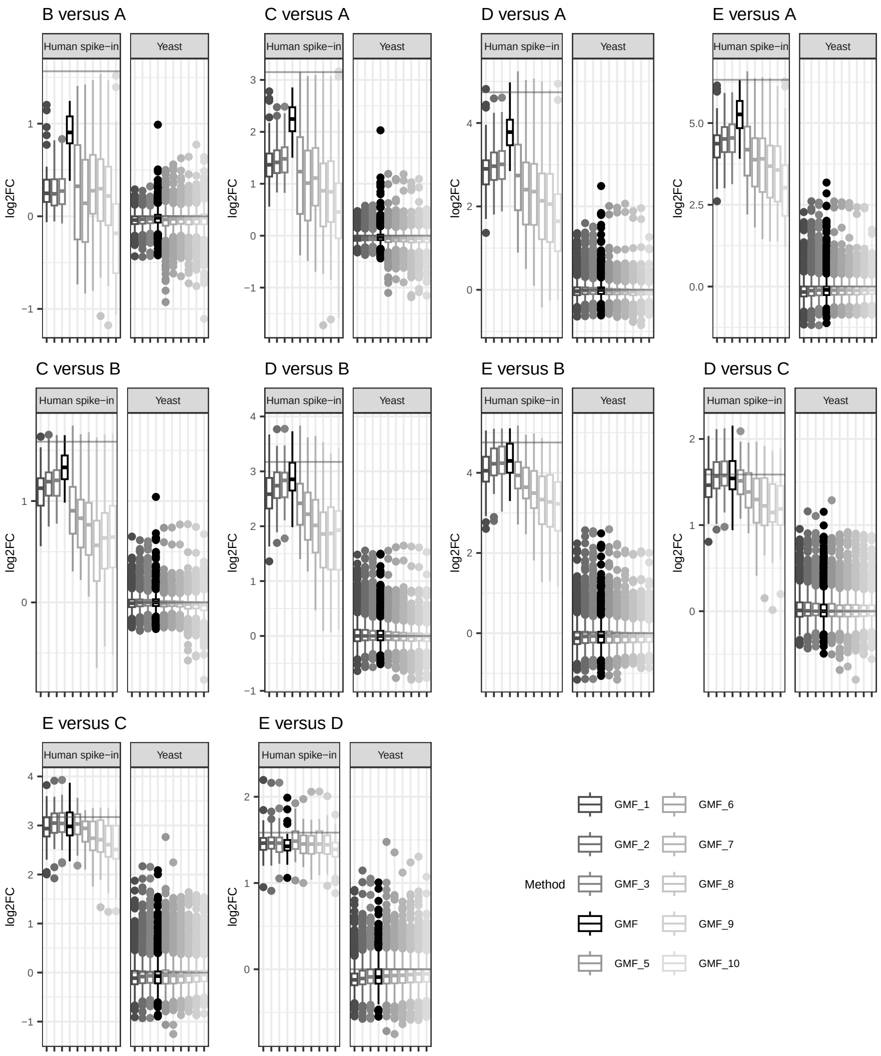

Supp. Fig. 36: Performance evaluation of differential abundance analyses using msqrob2 [25, 26] on the CPTAC dataset [16], for different numbers of latent factors used in omicsGMF. omicsGMF's cross-validation suggested four latent factors. Data from Lab 1, which suffers from ionization issues, are excluded. Human UPS proteins are differentially spiked between conditions while yeast background proteins serve as true negative control. Each plot shows estimated log2 fold changes (FC) by msqrob2 for both human spike-in proteins, and for reference yeast proteins. Grey line indicates known log2 FC.

Reviewer #2

"In this manuscript, the authors present omicsGMF, a method designed for both bulk and single-cell proteomics data, which integrates imputation, batch correction and principal component analysis into a unified framework to facilitate dimensionality reduction. While the integration of these steps is practically useful, the proposed model does not exhibit substantial methodological innovation. Moreover, the experimental results do not sufficiently demonstrate the method's potential to contribute to novel biological insights. I have the following comments":

Reviewer 2 - Comment 1

"omicsGMF models intensities as a function of known sample-level covariates, feature-level covariates, latent factors and their loadings. However, in the experiments presented in this manuscript, only a subset incorporated sample-level covariates, and feature-level covariates were not utilized in any of the reported analyses. In the absence of both sample-level and feature-level covariates, the model essentially reduces to a form of principal component analysis. As such, the core methodological innovation of the model is not clearly articulated, and the rationale behind its expected effectiveness remains unclear."

Our response:

(a) We agree with the reviewer that, when omitting sample and feature-level covariates, the model essentially reduces to a form of principal component analysis, more particularly to expectation-maximization PCA (EM-PCA). However, in the current version of the manuscript we already argue that conventional PCA does not work in the presence of missing data and that the NIPALS method, which simply discards the missing data, leads to poor performance and excessive run times. EM-PCA is very slow and does not scale to the ever-increasing dataset sizes in bulk and single-cell proteomics studies. Moreover, on both the Leduc and

CPTAC datasets, conventional implementations of EM-PCA do not converge. Hence, our innovative approach with stochastic gradient descent and minibatch subsampling effectively unlocks the rationale of EM-PCA for today's large (single-cell) proteomics datasets, allowing much faster convergence. To make this more explicit in the manuscript, we have now included the visualization results of an implementation of EM-PCA on all three analyzed datasets, as well as a benchmark on runtime, which clearly shows the advantages of our approach, even when no covariates are included.

(b) Importantly, omicsGMF allows for the inclusion of sample and feature-level covariates, which is another key innovation that avoids complex and ad hoc workflows for imputation, batch correction, and dimensionality reduction. Indeed, with existing approaches it is unclear which steps are required for optimal data processing, and in which order these have to be performed. This confuses users, and will likely lead to suboptimal processing. In contrast, omicsGMF provides a much more user-friendly, single-step approach, and moreover enables data-driven guidance on the inclusion of relevant factors through cross-validation. We now include new results with workflows that incorporate batch correction prior to imputation with the state-of-the-art tools, which clearly demonstrate the benefit of our unified omicsGMF framework. Furthermore, we now showcase on the CPTAC dataset that differential abundance analyses become inferior when arbitrarily choosing the number of latent factors without cross-validation, as is the case with conventional PCA-like algorithms.

(c) We now have additional results on using protein as a feature-level covariate. However, cross-validation provides clear evidence against including protein as a covariate. These cross-validation results are now included in Supplementary materials. We also would like to emphasize that currently, the proteomics community has not yet established a set of interesting feature-level covariates, probably as

these cannot be easily integrated in current data analysis tools. omicsGMF allows proteomic researchers to explore such feature-level covariates of interest. However, the goal of our manuscript is not to propose such covariates ourselves, but rather give researchers the appropriate tools to do so.

We have adapted the manuscript as follows:

- Points a. is addressed in paragraph 7 of section "Dimensionality reduction and visualization of proteomics data", and Supplementary Figures 13 and 14:

  Note further that omicsGMF reduces to a kind of expectation-maximization (EM) PCA when no sample or feature-level covariates are included. However, conventional EM-PCA struggles to converge, probably due to the excessive number of missing values (Supplementary Fig. 13). Furthermore, EM-PCA is very slow, and does not scale well to larger datasets. Neither does NIPALS, which performs PCA by ignoring missing values. In contrast, omicsGMF solves these problems by optimizing its parameters through an efficient adaptive stochastic gradient descent algorithm. This makes omicsGMF fast and scalable to large datasets, especially with regards to PCA-like algorithms EM-PCA and NIPALS, and to imputation methods such as DAE, VAE and CF (Supplementary Fig. 14).

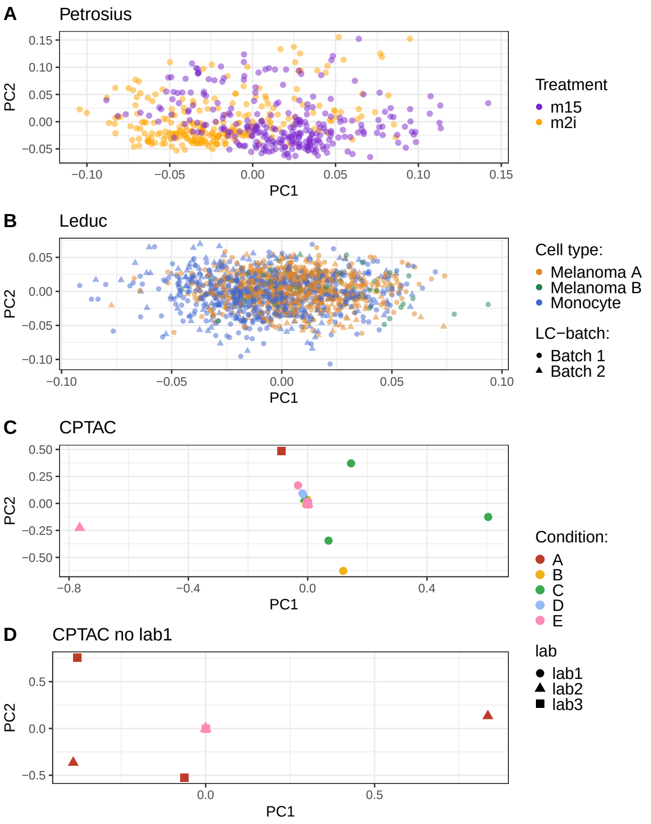

Supp. Fig. 13: Panels A, B, C and D show data from the label-free, single-cell Petrosius study [4], the labeled single cell Leduc dataset [3], the data from the label-free bulk CPTAC spike-in study [16], and the CPTAC spike-in study (data from Lab 1 excluded due to ionization issues). All visualizations are obtained with expectation-maximization PCA (EM-PCA). Note, however, that EM-PCA did not converge for the Leduc and CPTAC data.

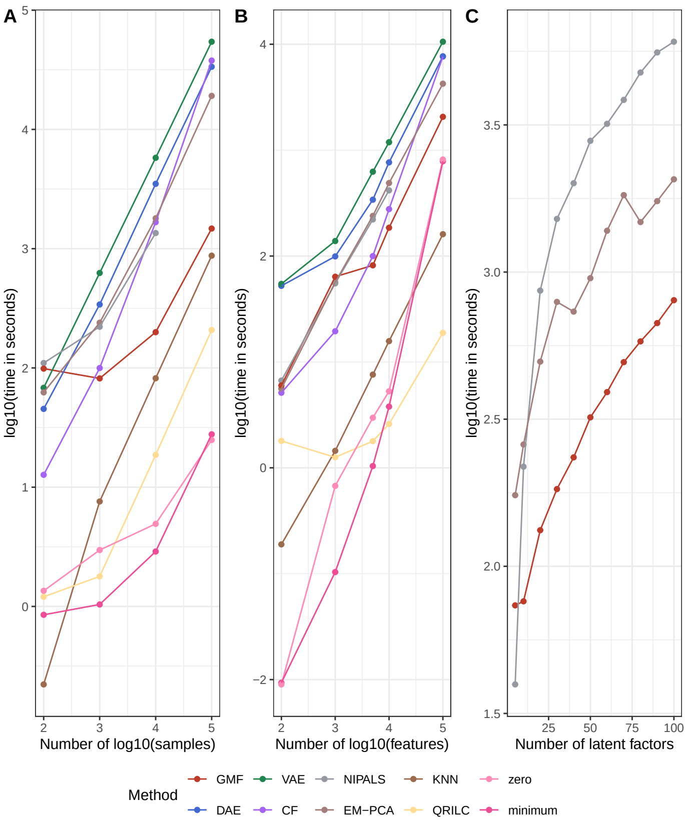

Supp. Fig. 14: Benchmark of computational time (in log10 of the number of seconds) in function of the number of samples (Panel A), the number of features (Panel B) and the number of latent factors for PCA-like algorithms (Panel C). Thousand samples are used in Panel B and C, five thousand features are used in Panel A and C, and ten latent factors are used in Panel A and B. omicsGMF is much faster compared to PCA-like algorithms that can deal with missing values (NIPALS and EM-PCA), and is faster than PIMMS imputation (DAE, VAE and CF) on large datasets. Methods like KNN, QRILC, zero and minimum imputation are faster than omicsGMF, but do not account for the correlation structure in the data. NIPALS could not return results in Panel A for 100000 samples.

- Point b. Is addressed in paragraph 3 of section "Imputation of missing values" and paragraph 4 of section "Differential analysis" and in Supplementary Figures 21, 22, 35 and 36:

  On the TMT-labeled Leduc dataset (Supplementary Fig. 19 and 20), omicsGMF with batch correction demonstrates better performance compared to all other imputation methods. This finding suggests that when complex technical artifacts are present, effective imputation of missing values requires explicit adjustment for these effects, and this for both MCAR and MNAR data. Note that, even though CF, DAE, and VAE were not designed for TMT-labeled data, they nonetheless outperform conventional imputation methods KNN, QRILC, zero, and minimum imputation. Interestingly, omicsGMF with batch correction remains the top performing workflow even when comparing it to workflows that adopt batch correction prior to imputation with state-of-the-art tools (Supplementary Figs. 21 and 22). This clearly shows the advantage of omicsGMF's strategy to simultaneously perform imputation of missing values with batch correction.

  ...

  Furthermore, Supplementary Figs. 35 and 36 assess the quality of the downstream differential abundance analysis across a range of different numbers of latent factors. Interestingly, it clearly shows that superior performance is obtained with an omicsGMF model that uses the number of latent factors suggested by cross-validation. This confirms that carefully selecting the number of latent factors is a valuable feature of omicsGMF, which can be important for subsequent downstream analyses.

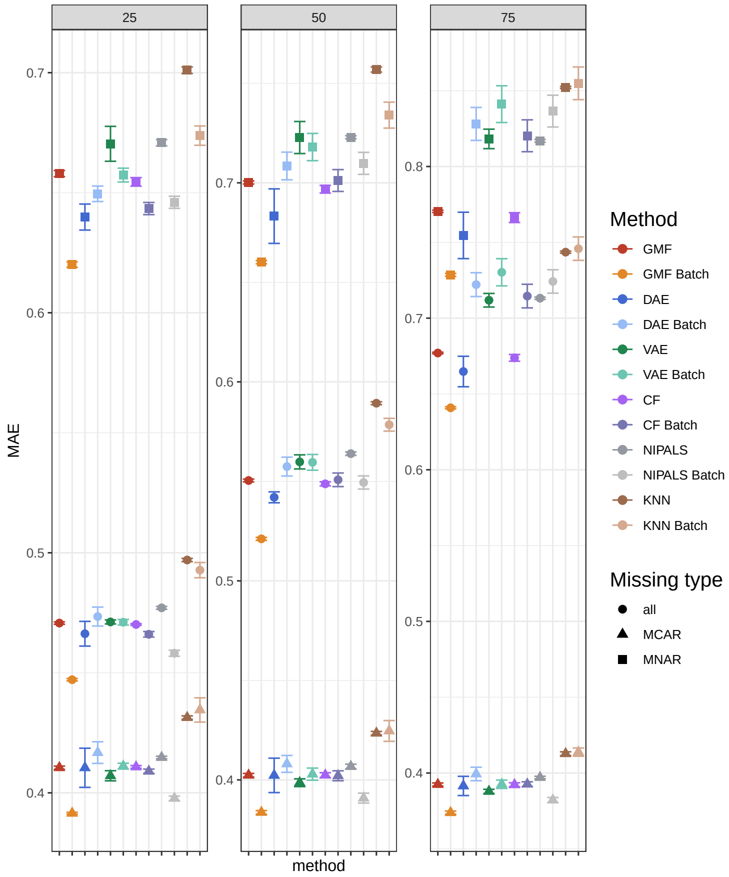

Supp. Fig. 21: Same figure as Supp. Fig 19, but with additional workflows in which batch effects were removed prior to imputation by linear regression including 142 dummy variables indicating TMT-run, except for omicsGMF that performs batch correction internally. These additional workflows are named with 'Batch'.

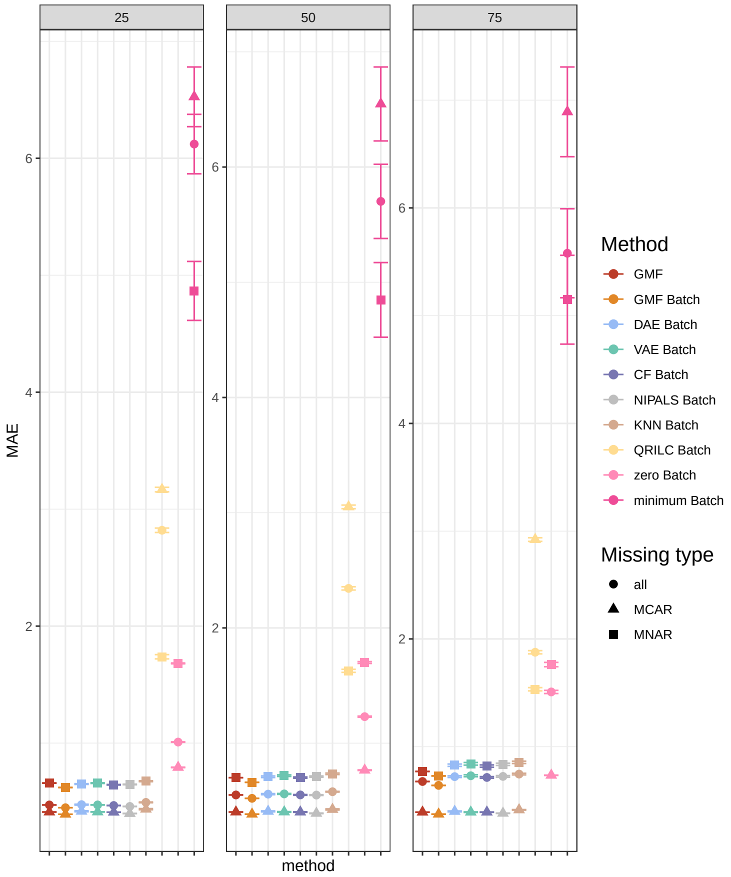

Supp. Fig. 22: Similar figure as Supp. Fig 21, but with QRILC, zero and minimum imputation.

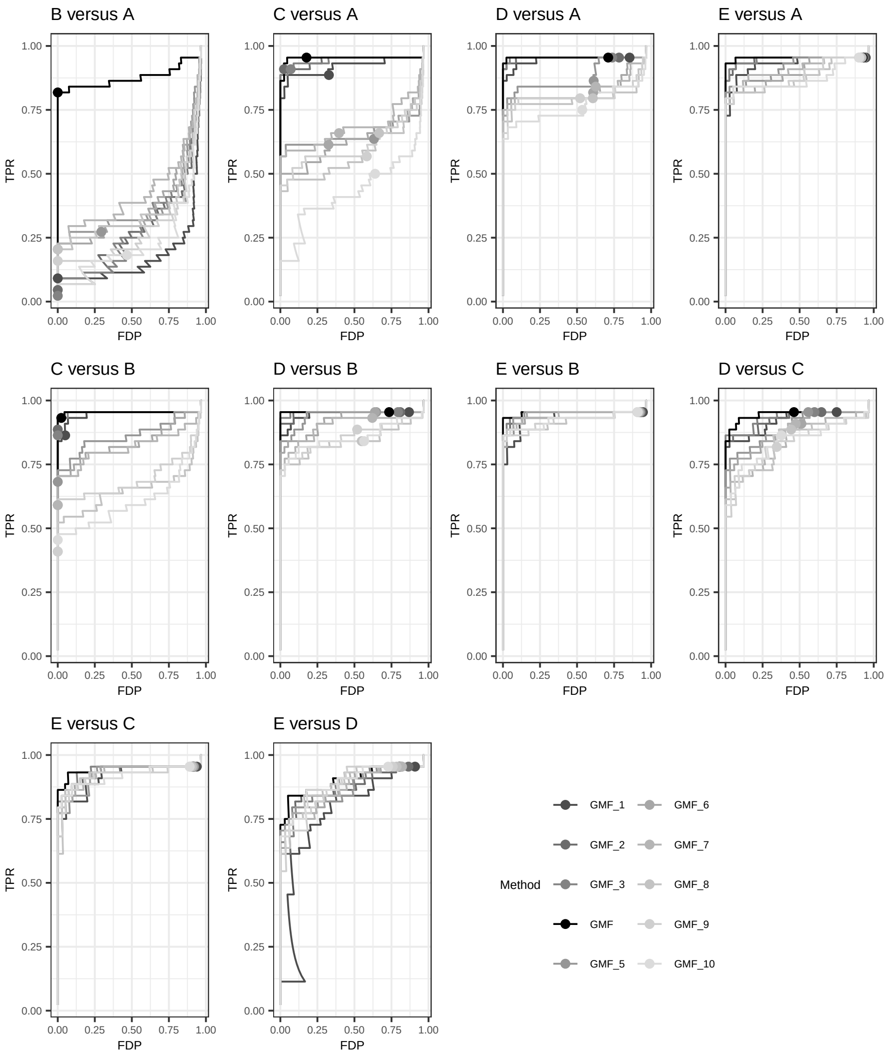

Supp. Fig. 35: Performance evaluation of differential abundance analyses using msqrob2 [25, 26] on the CPTAC dataset [16], for different numbers of latent factors used in omicsGMF. omicsGMF's cross-validation suggested four latent factors. Data from Lab 1, which suffers from ionization issues, are excluded. Human UPS proteins are differentially spiked between conditions while yeast background proteins serve as true negative control. Each curve shows true positive rate (TPR) in function of false discovery proportion (FDP). Dots on each curve represent working points for FDR level at nominal 5% level. Results for all pairwise comparisons between spike-in concentrations are shown.

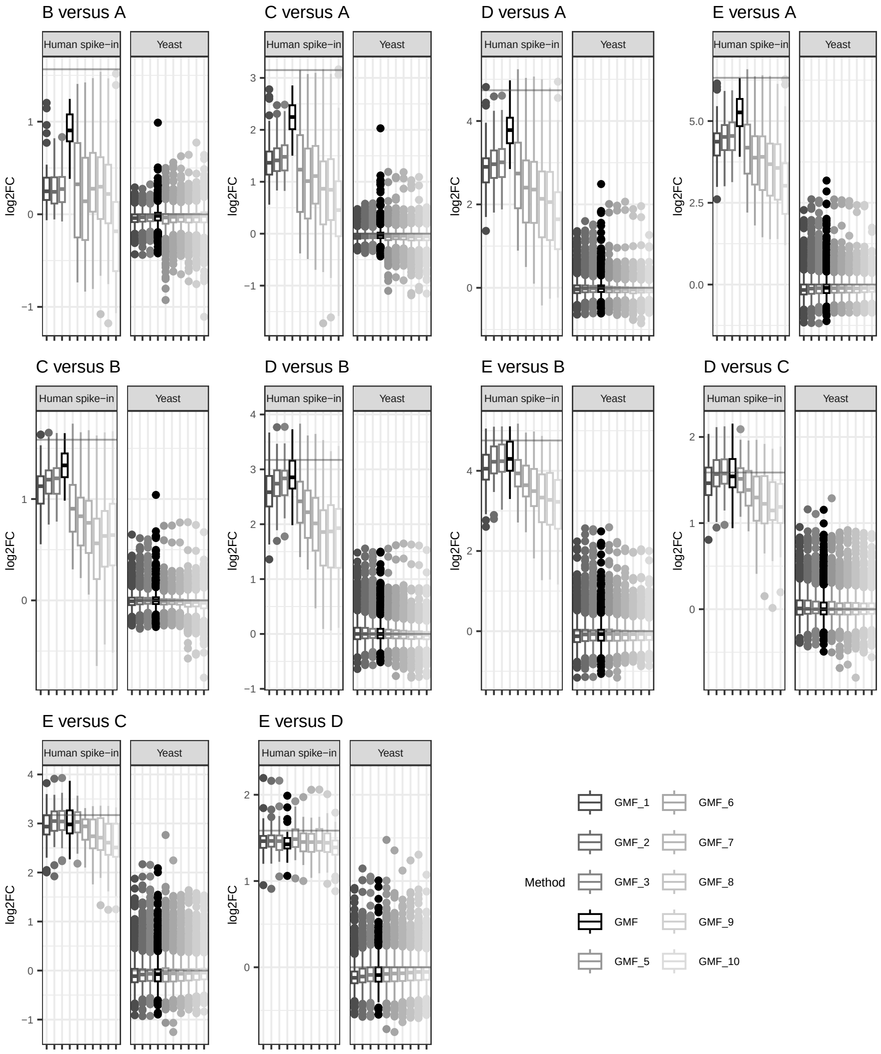

Supp. Fig. 36: Performance evaluation of differential abundance analyses using msqrob2 [25, 26] on the CPTAC dataset [16], for different numbers of latent factors used in omicsGMF. omicsGMF's cross-validation suggested four latent factors. Data from Lab 1, which suffers from ionization issues, are excluded. Human UPS proteins are differentially spiked between conditions while yeast background proteins serve as true negative control. Each plot shows estimated log2 fold changes (FC) by msqrob2 for both human spike-in proteins, and for reference yeast proteins. Grey line indicates known log2 FC.

- *Points c. is addressed in paragraph 5 of the novel section "Model selection by cross-validation", and Supplementary Figure 16:*

  omicsGMF can also account for feature-level covariates. Here, we considered the inclusion of dummy variables for the protein ID to which precursors or peptides belong. However, cross-validation provides clear evidence against including these covariates in all three datasets (Supplementary Fig. 16). Nevertheless, omicsGMF allows researchers to assess their own feature-level covariates of interest, which they can evaluate through cross-validation.

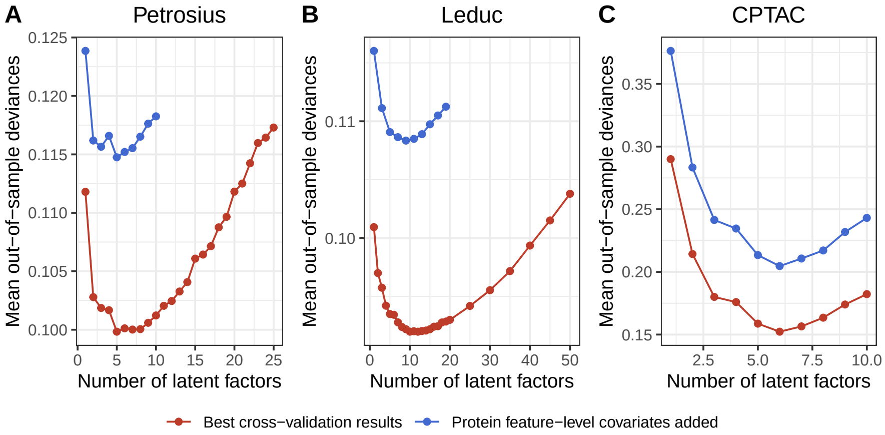

Supp. Fig. 16: Cross-validation with omicsGMF allows for comprehensive selection of the number of latent factors d in U for dimensionality reduction in combination with selection of known sample- or feature-level covariates. Each panel shows the mean of the out-of-sample deviances over three cross-validation folds in function of the number of latent factors d included in the model. In each fold, 30% of the values are masked for out-of-sample prediction. Panel A shows the cross-validation results for the Petrosius dataset [4], Panel B shows the cross-validation results for the Leduc dataset [3], and Panel C shows the cross-validation results for the CPTAC data [16], considering all three labs. Results are shown for the best cross-validation obtained for omicsGMF in Figure **??**, i.e., without known sample-level covariates for Petrosius, with correction for the known batch-effect associated to multiplexing cells in the same run for Leduc (142 dummy variables) and without correcting for lab- and spike-in effects for CPTAC. Then, feature-level covariates, i.e., dummy variables for the protein to which peptides belong are added to these models. Cross-validation provides clear evidence against including these covariates in all three datasets.

Reviewer 2 - Comment 2

"At the beginning of parameter initialization, missing values are imputed using μ_0. However, the rationale behind the definition of μ_0 is not clearly explained. It also remains unclear whether the choice of μ_0 has a significant impact on the final results."

Our response: We agree with the reviewer that the choice of initialization can have an impact on the final results. However, this is the case for most of the evaluated methods. Also, currently the feature mean of the observed values are used as initialization, which seems a sensible and yet simple approach that already gives good performance.

Reviewer 2 - Comment 3

"In the Introduction, the authors categorize missingness into two types: missing completely at random (MCAR) and missing not at random (MNAR). They point out that these two forms of missingness require distinct imputation strategies, and that the application of methods tailored to only one type can significantly distort the distribution of protein-level intensities. While omicsGMF is claimed to address both MCAR and MNAR simultaneously, the underlying mechanism by which it achieves this is not clearly described. Further clarification is needed to understand how the model differentiates and appropriately handles these two fundamentally different types of missingness."

Our response: In our revision we now clarify how omicsGMF addresses missing data. In proteomics, missingness due to low abundance is typically categorized as Missing Not At Random (MNAR), particularly when considering data from a single feature (e.g., a precursor or peptide) in isolation. However, omicsGMF models the data jointly across all features, leveraging correlations among multiple precursors, peptides, and proteins. This joint modeling approach allows omicsGMF to partially explain missingness by low abundance in one specific feature using the information in known covariates

and in other features through latent factors, effectively reducing what would be considered MNAR in a univariate context to Missing At Random (MAR), conditional on the observed covariates and latent factors. In cases where neither covariates nor latent factors contain relevant information, the imputation strategy reduces to a kind of Missing Completely At Random (MCAR) strategy. The manuscript is expanded as follows:

- Paragraph 2 in the "Results" section:

  The advantages are threefold. First, the imputed values account for known sample- and feature-level covariates, as well as the latent structure, which implies imputation under a Missingness At Random (MAR) assumption conditional on both known covariates and information in other features through latent factors. ~~resulting~~ This results in accurate imputed intensities even when dealing with significant technical effects.

- We adjusted the first paragraph of section "Imputation of missing values" in the revised manuscript:

  The final mean estimates obtained from omicsGMF can also be used for the imputation of missing values while accounting for latent factors, as well as for known sample- and feature-level covariates. This imputation strategy assumes missingness at random that can be explained by known covariates and latent structure. Although it does not explicitly assume missingness due to low abundance, which is particularly present when considering data from a single feature (e.g. a precursor or peptide) in isolation, omicsGMF leverages the known covariates and the correlation structure across multiple precursors, peptides and proteins. This joint modelling of features allows to partially explain missingness by low abundance in one specific feature using the information in known covariates and in other features through latent factors, effectively reducing what would be considered MNAR in a univariate context to MAR, conditional on the observed covariates

and latent factors. In cases where neither covariates or latent factors contain relevant information, the imputation strategy reduces to a kind of MCAR strategy.

To evaluate imputation performance, we simulate missingness both completely at random (MCAR) and missing not at random (MNAR) due to low abundance (see Methods). The mean absolute error (MAE) between the imputed and original values is then assessed across ten different random seeds, considering scenarios where 25%, 50%, and 75% of the masked values are attributed to low abundance. The MAEs for all simulations are available in Supplementary Data.

- The subsection 'Optimization of the parameters' in the Methods is adapted as follows:

  At convergence, the estimated means, $\hat{\boldsymbol{\mu}}_t$, can be used for imputation purposes, which assumes missing at random conditional on the known covariates and latent structure.

Reviewer 2 - Comment 4

"The authors propose several approaches for selecting the number of latent factors, including examining the eigenvalues in a scree plot, using information criteria such as AIC or BIC, and applying cross-validation. Do these methods yield consistent estimates of the number of latent factors? How sensitive are the final results to the choice of selection method?"

Our response: We have analyzed the results of AIC and BIC to determine the optimal number of latent factors. Although these metrics are useful in many other applications, such as single-cell RNA sequencing data, here, a problem seems to occur: due to the extensive number of missing values in proteomics data, the deviances are no longer calibrated with the degrees of freedom that are used by the algorithm. Therefore, using more latent factors increases the spent degrees of freedom much more than it decreases the sum of deviances of the very few observed data points, which results in a very high penalization for an increase in the number of latent factors.

Therefore, both AIC and BIC tend to suggest a very low number of latent factors (see Rebuttal Figure 1 and 2 below). Further, as visually inspecting the scree plot is not a quantitative optimization measure, which might confuse the reader if we would extensively discuss this measure.

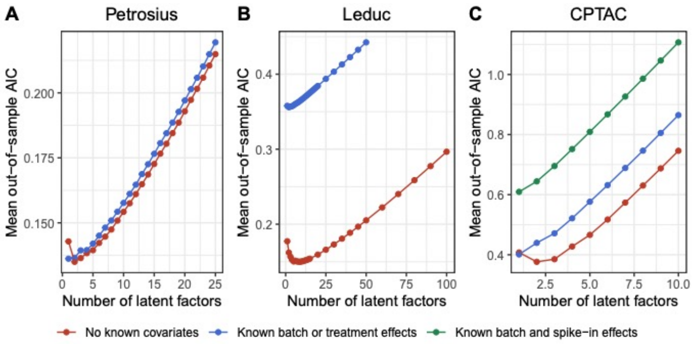

Rebuttal Figure 1: Cross-validation with omicsGMF for selection of the number of latent factors d in U for dimensionality reduction. Each panel shows the AIC in function of the number of latent factors included in the model. Panel A shows the cross-validation results for the Petrosius dataset [4] with and without accounting for the treatment effect (one dummy variable). Panel B shows the cross-validation results for the Leduc dataset [3], with and without correcting for the known batch-effect associated to multiplexing cells in the same run (142 dummy variables). Panel C shows the cross-validation results for the CPTAC data [16], considering all three labs. Results are shown for omicsGMF without known covariates, accounting for the lab effects (two dummy variables), and accounting for both the lab (two dummy variables) and spike-in concentration effects (four dummy variables).

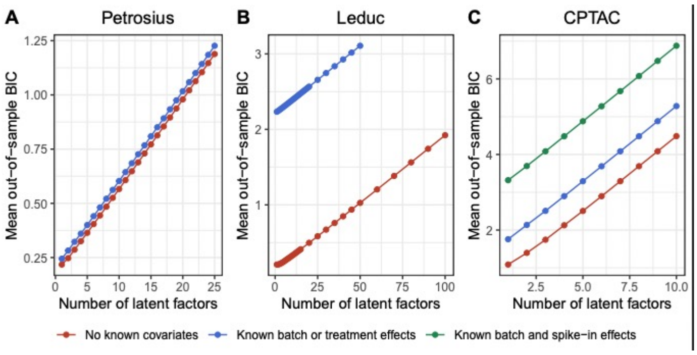

Rebuttal Figure 2: Cross-validation with omicsGMF for selection of the number of latent factors d in U for dimensionality reduction. Each panel shows the BIC in function of the number of latent factors included in the model. Panel A shows the cross-validation results for the Petrosius dataset [4] with and without accounting for the treatment effect (one dummy variable). Panel B shows the cross-validation results for the Leduc dataset [3], with and without correcting for the known batch-effect associated to multiplexing cells in the same run (142 dummy variables). Panel C shows the cross-validation results for the CPTAC data [16], considering all three labs. Results are shown for omicsGMF without known covariates, accounting for the lab effects (two dummy variables), and accounting for both the lab (two dummy variables) and spike-in concentration effects (four dummy variables).

Hence, we have opted to no longer mention AIC, BIC and use of the scree plot to select the number of latent factors in the Results section. In the Methods section, we now refer to it as an option that can be used in the software implementation, while explicitly recommending against it for proteomics data analysis as these seem to be suboptimal due to the high number of missing values.

The manuscript is adapted as follows:

- Paragraph 3 of the "Results" section:

  A critical issue in conventional algorithms and pipelines is their lack of a principled way to estimate the optimal number of latent factors, d, in the model. ~~Remarkably, the omicsGMF framework implements several model selection methods relying on scree plot investigation, eigenvalue thresholding, minimization of information-based criteria, e.g., AIC and BIC, and~~ The omicsGMF framework implements a model selection method that relies on the minimization of the cross-validation error to evaluate the out-of-sample error on subsets of holdout values masked and estimated as if they were missing observations. This results in comprehensive and easy-to-use model selection, allowing for optimal downstream analyses.

- Paragraph 1 of the novel section "Model selection by cross-validation":

  The other ~~competing~~ workflows also do not infer or provide guidelines on the number of latent factors for imputation nor for downstream applications, such as clustering, UMAP and t-SNE visualizations, or for correction for unknown batch effects and confounders, amongst others. Interestingly, omicsGMF allows selection of the optimal number of latent factors and/or the inclusion for known covariates by using cross-validation~~, information criteria, such as AIC and~~

~~BIC, or scree plots, all of~~ which can guide the user in different downstream applications.

- Subsection "Cross-validation to determine the number of latent covariates" in the "Methods" section:

  To use omicsGMF, the number of latent variables included in the model, d, has to be selected. omicsGMF has the option to select these using the eigenvalues in a scree-plot, information criteria, such as AIC or BIC, or cross-validation. However, as AIC and BIC are not well calibrated when dealing with missing values, and visually inspecting the scree-plot is prone to interpretation, we here ~~In this manuscript, we~~ consider 3-fold cross-validation of the out-of-sample deviances to select d. During cross-validation, a subset of the observed data is masked as missing. Then, for various numbers of latent factors, omicsGMF imputes these values after convergence and calculates the residual sum of squared errors between the imputed and original values for the masked entries. The number of latent variables that minimizes the sum of squared errors is then considered the optimal number of latent variables and is used for the final fit without masking any observed values. The cross-validation results on the different datasets are available in Fig. 4.

Reviewer 2 - Comment 5

"The authors repeatedly characterize omicsGMF as a fast method. However, the manuscript lacks any quantitative analysis, benchmarking, or discussion of runtime performance."

Our response: We have now included a quantitative runtime benchmark in Supplementary information, showcasing our superior scalability in function of the number of samples, features and number of latent factors with respect to competing methods:

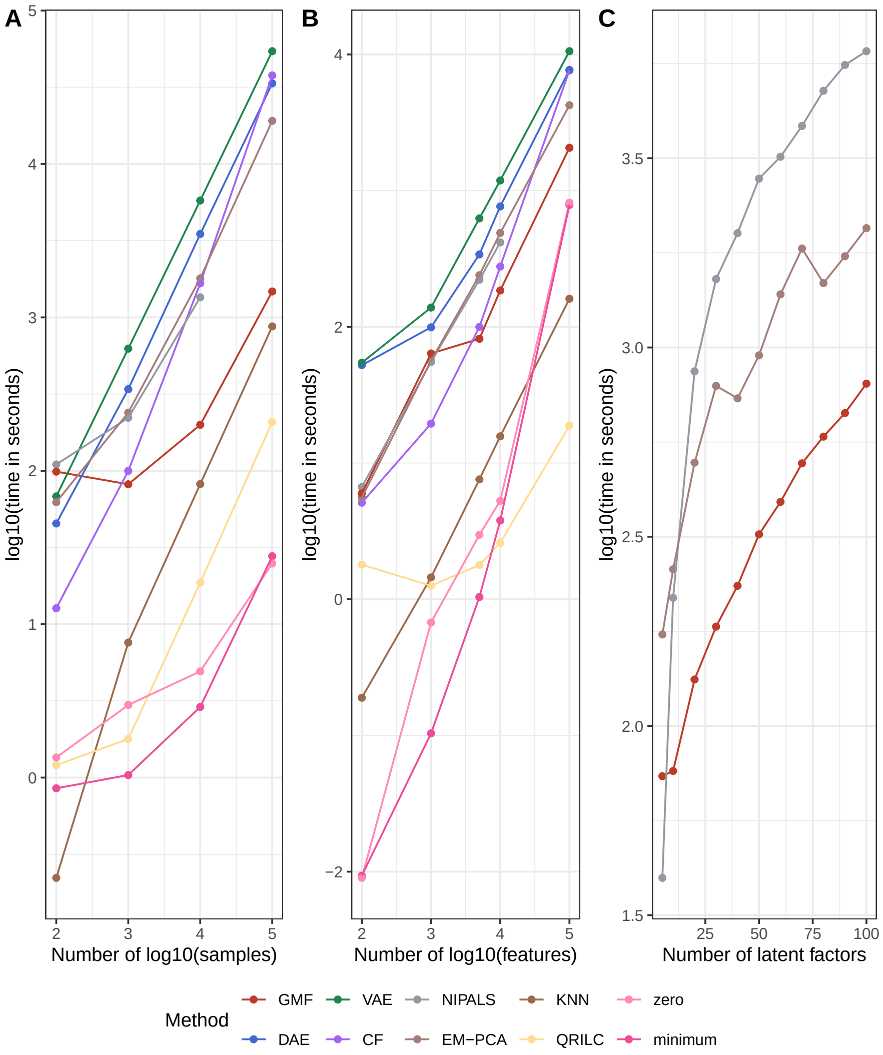

Supp. Fig. 14 Benchmark of computational time (in log10 of the number of seconds) in function of the number of samples (Panel A), the number of features (Panel B) and the number of latent factors for PCA-like algorithms (Panel C). Thousand samples are used in Panel B and C, five thousand features are used in Panel A and C, and ten latent factors are used in Panel A and B. omicsGMF is much faster compared to PCA-like algorithms that can deal with missing values (NIPALS and EM-PCA), and is faster than PIMMS imputation (DAE, VAE and CF) on large datasets. Methods like KNN, QRILC, zero and minimum imputation are faster than omicsGMF, but do not account for the correlation structure in the data. NIPALS could not return results in Panel A for 100000 samples.

The following text has been included in the Results section:

Note further that omicsGMF reduces to a kind of expectation-maximization (EM) PCA when no sample or feature-level covariates are included. However, conventional EM-PCA struggles to converge, probably due to the excessive number of missing values (Supplementary Fig. 13). Furthermore, EM-PCA is very slow, and does not scale well to larger datasets. Neither does NIPALS, which performs PCA by ignoring missing values. In contrast, omicsGMF solves these problems by optimizing its parameters through an efficient adaptive stochastic gradient descent algorithm. This makes omicsGMF fast and scalable to large datasets, especially with regards to PCA-like algorithms EM-PCA and NIPALS, and to imputation methods such as DAE, VAE and CF (Supplementary Fig. 14).

Reviewer 2 - Comment 6

"In several experiments, the incorporation of known covariate information appears to result in reduced performance. For example, this is observed in the imputation task on the label-free CPTAC dataset, as shown in Supplementary Fig. 16. How can this phenomenon be explained? Does it indicate a limitation in the method's design or implementation regarding the integration of covariates?"

Our response: This is not a limitation of our method, but rather a characteristic of the specific datasets. Indeed the spike-in treatment effect in the label-free CPTAC dataset is modelled using four dummy variables: one for spike-in condition B, C, D and E (condition A is the reference condition). However, this effect can be captured with a single continuous covariate that contains information on the spike-in treatment. Moreover, the known batch effect for lab (lab1, lab2, lab3), did not include the changes in MS operation in lab1 to mitigate issues with poor ionisation. Both sources of variability could be efficiently captured by omicsGMF with fewer latent covariates than the number of known covariates. Hence, using the known covariates is suboptimal, which is acknowledged by omicsGMF's cross validation. However, for the Leduc

study involving many cells and many batches, each containing a few number of cells, the use of the batch effect is key and could not be efficiently captured using latent variables in omicsGMF. This was again highlighted in the cross validation.

Reviewer 2 - Comment 7

"In the imputation task on the TMT-labeled Leduc dataset, omicsGMF outperformed the other methods when accounting for batch effects. However, when batch effects were not considered, omicsGMF showed no clear advantage. This raises an important question: how would the performance of the other methods change if batch effects were adjusted prior to imputation, or if imputation were performed within each batch separately?"

Our response: We thank the reviewer for this suggestion. We have expanded our analysis on the Leduc dataset with additional workflows that perform batch correction prior to imputation. These results clearly highlight the benefit of our unified omicsGMF framework that can perform all steps simultaneously. The second suggestion to impute within batch is indeed compelling, but, unfortunately, the Leduc data has only up to 14 cells available within the same batch (TMT run). Hence, the huge number of missing values, combined with the limited number of cells per batch render within-batch imputation unfeasible.

We have adapted the manuscript as follows to include these analyses:

- Paragraph 3 of section "Dimensionality reduction and visualization of proteomics data":

  omicsGMF can also directly account for known covariates, such as batch effects, which we illustrate for the TMT-labeled Leduc dataset (Fig. 3, Panel B; Supplementary Fig. 5). Indeed, Leduc et al. multiplexed up to 14 cells in each TMT run. Upon correcting for the run effect, omicsGMF uncovers additional biological variability corresponding to a previously reported subpopulation of melanoma cells [3]. Imputation of missing values followed by PCA cannot directly control

for the technical run-to-run variability, resulting in a dimensionality reduction where the first two latent variables are mainly driven by this strong batch effect. Hence, conventional PCA-based workflows with imputation methods require an additional step for batch removal prior or post imputation. NIPALS, however, which conceptually does not require imputation of missing values, also cannot account for known batch effects, and therefore suffers from the same caveats. When using a more complicated multi-step workflow that sequentially performs imputation, batch correction, and PCA, similar visualizations as with omicsGMF can be obtained for CF, DAE, VAE, NIPALS and KNN, while QRILC, zero, and minimum imputation clearly display imputation artifacts in their low dimensional visualizations (Supplementary Fig. 6). Similar results are also obtained when performing batch correction prior to imputation for DAE, VAE, CF, NIPALS and KNN (Supplementary Fig. 7). However, small differences remain present, and even large differences are obtained in the visualization of QRILC, zero and minimum imputation. This showcases the benefit of using a single algorithm to tackle imputation and batch effect removal simultaneously, avoiding an arbitrary choice of the sequence in which each of these tasks are performed in current state-of-the-art multi-step workflows.

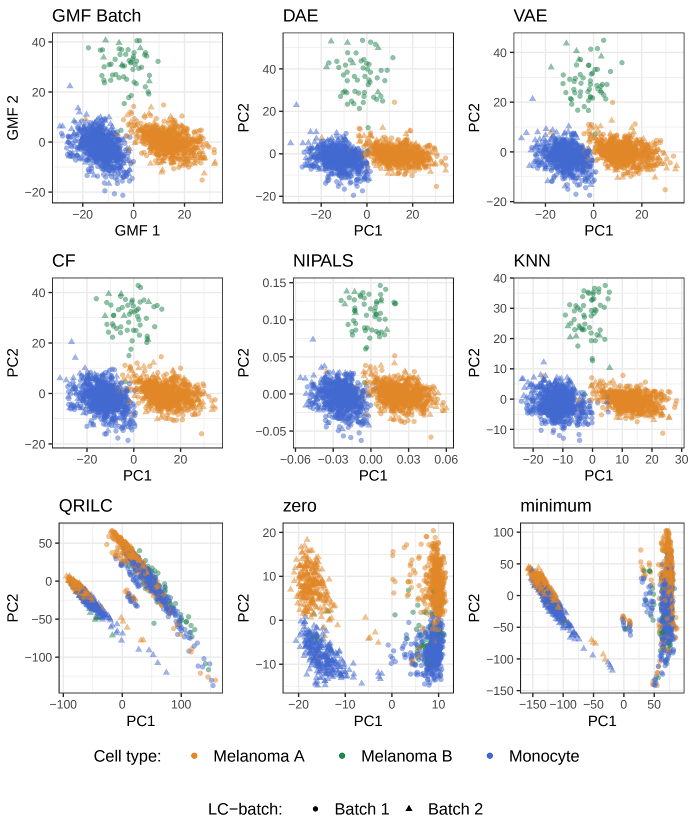

Supp. Fig. 7: Low-dimensional visualization of the Leduc data [3] after batch-correction, colored by cell type. omicsGMF directly accounts for known batch effects. The other visualizations are obtained by sequentially performing batch-correction using linear regression, imputation of missing values using DAE, VAE, CF, NIPALS, KNN, QRILC, and minimum prior to PCA.

- Paragraph 3 of section "Imputation of missing values":

  On the TMT-labeled Leduc dataset (Supplementary Fig. 19 and 20), omicsGMF with batch correction demonstrates better performance compared to all other imputation methods. This finding suggests that when complex technical artifacts are present, effective imputation of missing values requires explicit adjustment for these effects, and this for both MCAR and MNAR data. Note that, even though CF, DAE, and VAE were not designed for TMT-labeled data, they nonetheless outperform conventional imputation methods KNN, QRILC, zero, and minimum imputation. Interestingly, omicsGMF with batch correction remains the top performing workflow even when comparing it to workflows that adopt batch correction prior to imputation with state-of-the-art tools (Supplementary Figs. 21 and 22). This clearly shows the advantage of omicsGMF's strategy to simultaneously perform imputation of missing values with batch correction.

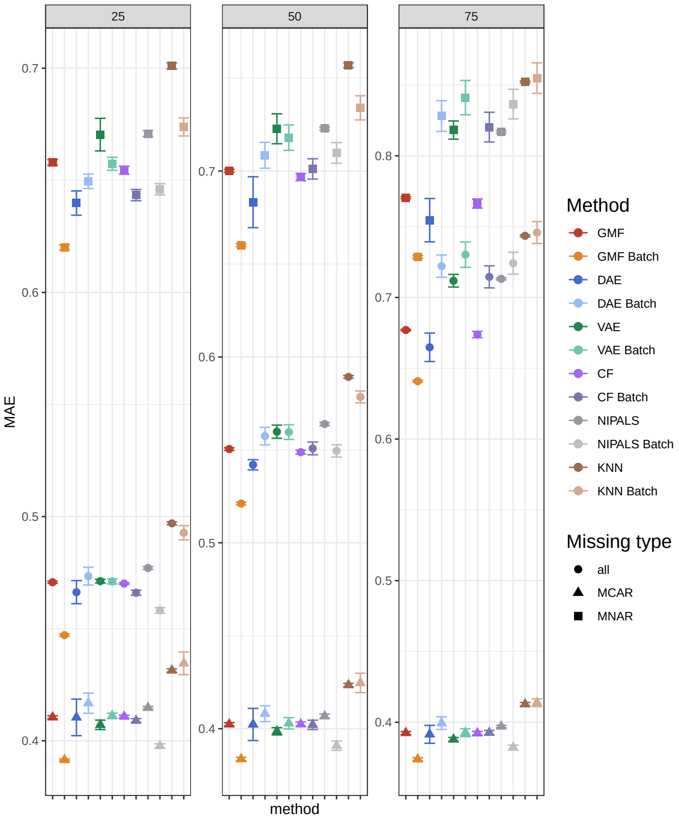

Supp. Fig. 21: Same figure as Supp. Fig 19, but with additional workflows in which batch effects were removed prior to imputation by linear regression including 142 dummy variables indicating TMT-run, except for omicsGMF that performs batch correction internally. These additional workflows are named with 'Batch'.

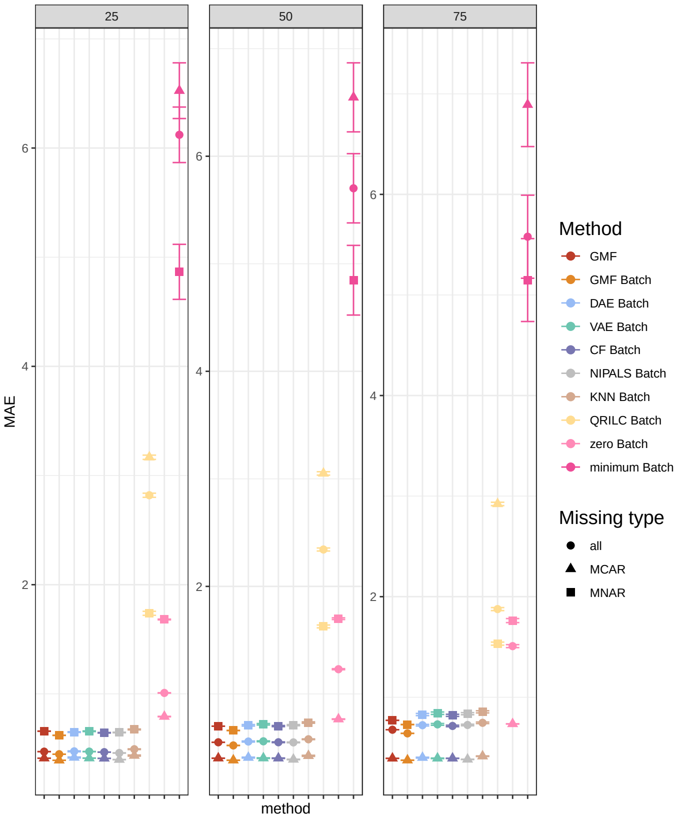

Supp. Fig. 22: Similar figure as Supp. Fig 21, but with QRILC, zero and minimum imputation.

Reviewer 2 - Comment 8

"In the Dimensionality reduction and visualization of proteomics data section, the assessment is primarily based on qualitative observations. It is recommended to include quantitative evaluations to provide a more objective and comprehensive comparison."

Our response: We have expanded our visual evaluation with a quantitative evaluation of the dimension reduction using the average silhouette width (ASW). ASW measures the trade-off between the cell similarity within a group against the cell similarity between groups. A general use-case of omicsGMF's dimension reduction is to highlight biologically relevant patterns while correcting for technical effects. So a good method should maximize the ASW when considering a biological grouping such as the known cell type while minimizing the ASW when considering a technical grouping such as the MS acquisition run. Using this measure on the Petrosius data again confirms that omicsGMF provides a better separation of both cell types. On the Leduc data, results are similar between the most efficient methods, which was already stated based on visually inspecting the plots. Note, however, that other methods require complex workflows to obtain the principal components, and that we have shown that their imputation performance on the Leduc data is inferior compared to omicsGMF.

We have adapted paragraph 4 of section "Dimensionality reduction and visualization of proteomics data" in the revised manuscript as follows:

Supplementary Fig. 8 quantitatively confirms these visual observations on both the Petrosius and Leduc dataset using the average silhouette width (ASW). On the Petrosius dataset, the ASW is higher for omicsGMF than for other workflows, which suggests that cells of the same cell type cluster better together. For the Leduc data, the ASW of omicsGMF is in line with workflows that sequentially perform batch correction and imputation, or vice versa, with DAE, VAE, CF, NIPALS and KNN imputation. These imputation methods perform better than QRILC, zero or minimum imputation workflows.

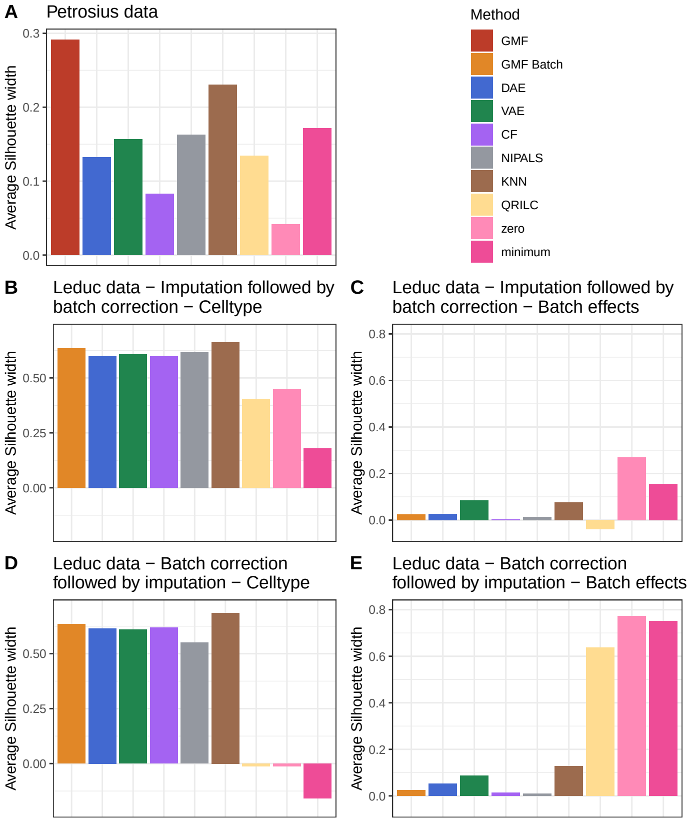

Supp. Fig. 8: Average silhouette width (ASW) on the first two principal components for A) The Petrosius dataset [4] and B-E) the Leduc dataset [3]. Panels A, B and D show ASW for different cell types, with higher ASW indicating better separation between different cell types, therefore resulting in better clustering. Panel C and E show mean ASW for batch evaluated in each cell type separately. Here, lower ASW indicates less separation of different batches within each cell type, thus indicating better clustering, as cells from the same batch should not cluster together after batch correction. omicsGMF (GMF) and omicsGMF while correcting for known batch effects (GMF Batch) directly infer the first two principal components, while the other methods perform imputation followed by PCA (Panel A), imputation followed by batch correction and PCA (Panel B and C) and batch correction followed by imputation and PCA (Panel D and E).

Reviewer 2 - Comment 9

"To more convincingly establish the practical utility of the proposed method, additional and more extensive experiments are needed to evaluate whether it can yield

more informative or biologically meaningful findings in downstream analyses compared to existing methods."

Our response: We would like to highlight that, in both the CPTAC and E. coli case studies, where ground truth on differential abundance is available, we demonstrated a clear advantage of applying omicsGMF prior to the prioritization of the differentially abundant spike-in proteins, compared to existing imputation methods. We have further extended this discussion by also including the number of true positives and false positives returned at the 5% false discovery proportion (FDP). These clearly show that omicsGMF results in more informative findings, as much more true positives are returned in most comparisons.

We have adapted the manuscript as follows:

- Paragraph 2 of section "Differential analysis":

  We first focus on the analysis using data from Labs 2 and 3. Fig. 6, panel A shows that only the workflows using non-imputed data and omicsGMF-imputed data provide reliable inference between the two lowest spike-in conditions, A and B. Other imputation methods generate numerous false positives in their top-ranked results, or even return false positives before any UPS protein is called differentially abundant (DAE, KNN). This outcome aligns with previous observations in Fig. 5, where most methods impute similar values for missing UPS peptides across these conditions. For comparisons between B vs. C and A vs. C, CF's performance improves considerably, but still falls short of omicsGMF, which is also confirmed by the number of retrieved true positives at the 5% false discovery proportion (Supplementary Fig. 32). Meanwhile, other state-of-the-art imputation methods continue to struggle with reliable DA inference. As the comparisons shift to higher spike-in concentrations, the performance of all methods improves. However, omicsGMF remains the top performer in the majority of the comparisons (Supplementary Fig. 33).

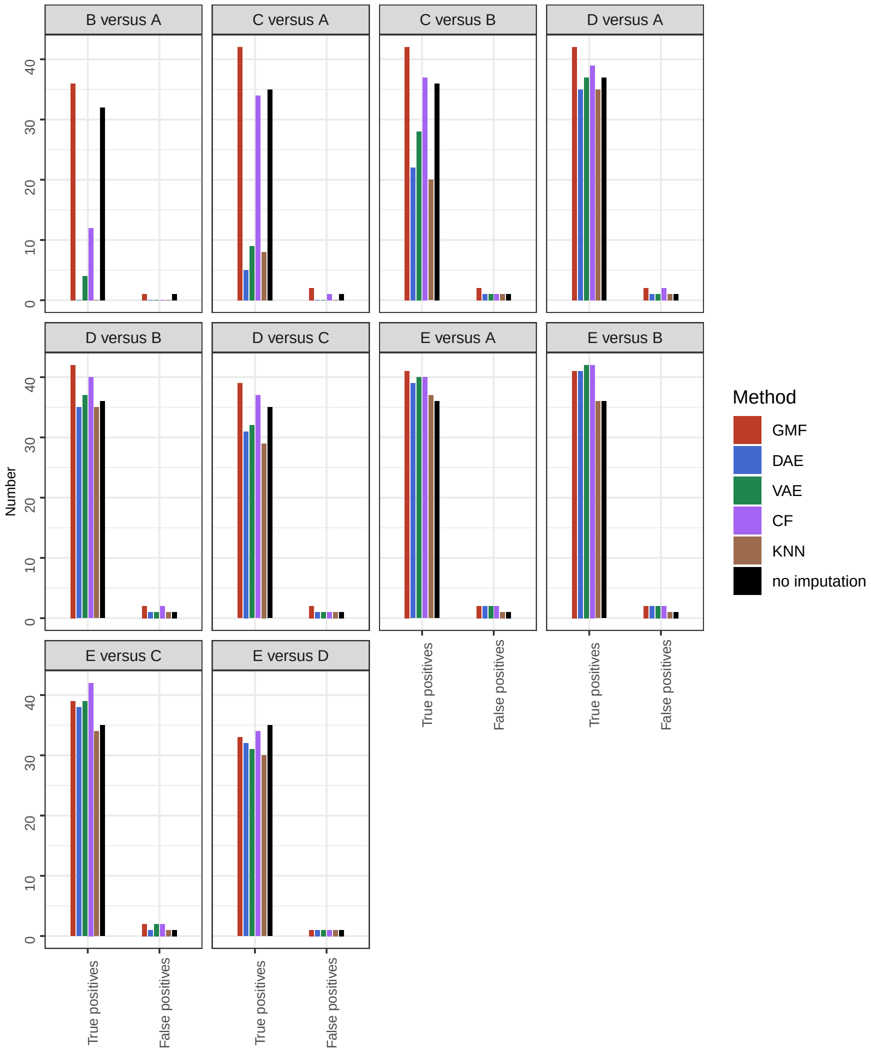

Supp. Fig. 32: Number of true positives and false positives retrieved in the CPTAC dataset [16] at a false discovery proportion of at most 5%. Data from Lab 1 suffering from ionization issues are excluded. Human UPS proteins are differentially spiked between conditions and yeast background proteins serve as true negative control.

- Paragraph 7 of section "Differential analysis":

  We repeated this benchmark using another dataset, in which an E. coli proteome was mixed at varying concentrations within a constant human background [27]. Consistent with our previous findings, the performance of DA following omicsGMF imputation surpasses that of other imputation strategies, particularly at low spike-in concentrations (Supplementary Fig. 41, 42), resulting in an increase of the number of true positives at the 5% false discovery proportion (Supplementary Fig. 43). Furthermore, the imputed values generated by omicsGMF again exhibit a closer alignment with the trends across conditions for quantified ~~yeast~~ E. coli and human ~~PSMs~~ peptides than those imputed with the existing methods (Supplementary Fig. 44). These results reaffirm that omicsGMF imputation leads to more powerful downstream DA analyses than existing imputation strategies.

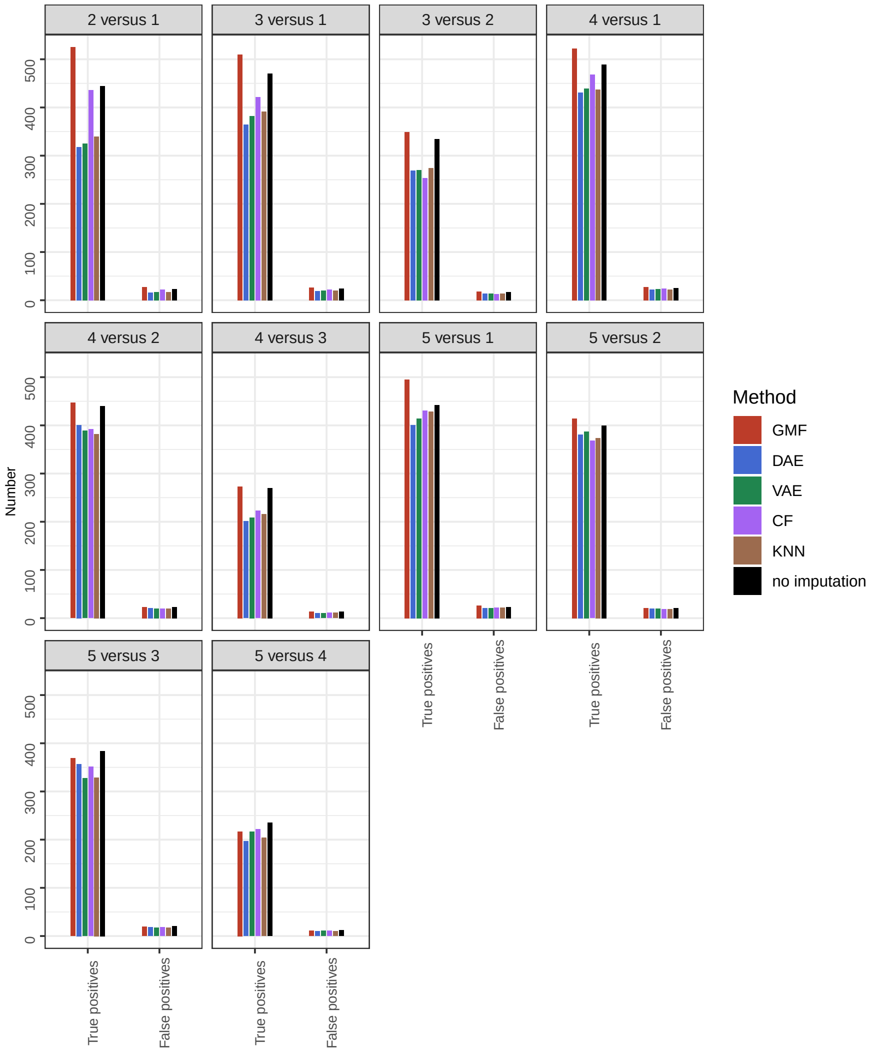

Supp. Fig. 43: Number of true positives and false positives retrieved in the Shen dataset [27] at a false discovery proportion of at most 5%. E. coli proteins are mixed in different concentrations and serve as true positives within a human background.

Reviewer #3

“This manuscript describes the omicsGMF tool, which integrates batch effect correction, imputation, and dimensionality reduction for processing bulk- and single-cell proteomic datasets. The concept of addressing sample, feature, and unknown covariates within a unified regression framework is an interesting and common approach to omics data processing. After careful review, I regret to recommend rejection of the manuscript due to several critical concerns regarding its novelty, methodological rigor, and alignment with the priorities of proteomic research. Below, I outline these concerns to provide clarity and constructive feedback for future work.

Major concerns:”

Reviewer 3 - Comment 1

“Limited Novelty: Covariate correction is a common practice in omics data analysis, and the omicsGMF tool does not sufficiently distinguish itself from existing methodologies. While the inclusion of an ”unknown covariate” and iterative imputation is noted, these features do not represent a sufficiently novel contribution to meet the journal’s scope and standards. A more robust justification of the tool’s unique advancements, supported by comprehensive comparisons with state-of-the-art methods, is necessary to establish its novelty.”

Our response: We do not agree with this comment: there are currently no other methods like omicsGMF for proteomics applications, as it can simultaneously address batch correction, missing data, and dimensionality reduction. Moreover, addressing the problem of missing data in large scale (single-cell) proteomics data sets is extremely timely and relevant as indicated by competing methods, which we included in our benchmark and which were all recently published in high impact journals (PIMMS [Webel et al. (2024); Nature Communications] and scPROTEIN [Li et al. (2024), Nature Methods]). More importantly, we provide multiple benchmarks for different

applications, which are according to the state-of-the-art, i.e. (a) qualitatively using visualisation of the data in low dimension, (b) evaluating imputation quality on multiple simulation studies using mean absolute errors and distribution plots of the imputed data, and (c) sensitivity - false discovery proportion plots for assessing the performance of differential abundance analysis upon imputation. Moreover, in the revised version of the manuscript we have included further quantitative evaluation of the methods based on average silhouette width.

We have adapted to manuscript to include the ASW as follows:

Supplementary Fig. 8 quantitatively confirms these visual observations on both the Petrosius and Leduc dataset using the average silhouette width (ASW). On the Petrosius dataset, the ASW is higher for omicsGMF than for other workflows, which suggests that cells of the same cell type cluster better together. For the Leduc data, the ASW of omicsGMF is in line with workflows that sequentially perform batch correction and imputation, or vice versa, with DAE, VAE, CF, NIPALS and KNN imputation. These imputation methods perform better than QRILC, zero or minimum imputation workflows.

Reviewer 3 - Comment 2

"Unfair Methodological Comparisons: The evaluation of omicsGMF against other methods lacks fairness and rigor. It is widely recognized that imputation alone is inadequate for batch effect correction in cell clustering, as normalization is a prerequisite step. However, the manuscript compares omicsGMF, which incorporates normalization, to a pipeline of imputation and PCA without applying normalization to the competing methods. This discrepancy invalidates the reported performance improvements. Furthermore, the three datasets used in the study have been analyzed in prior work, which demonstrated effective cell clustering and identification of differentially expressed proteins (DEPs) with biological significance. The absence of DEP analysis in this study represents a critical oversight, undermining the tool's utility."

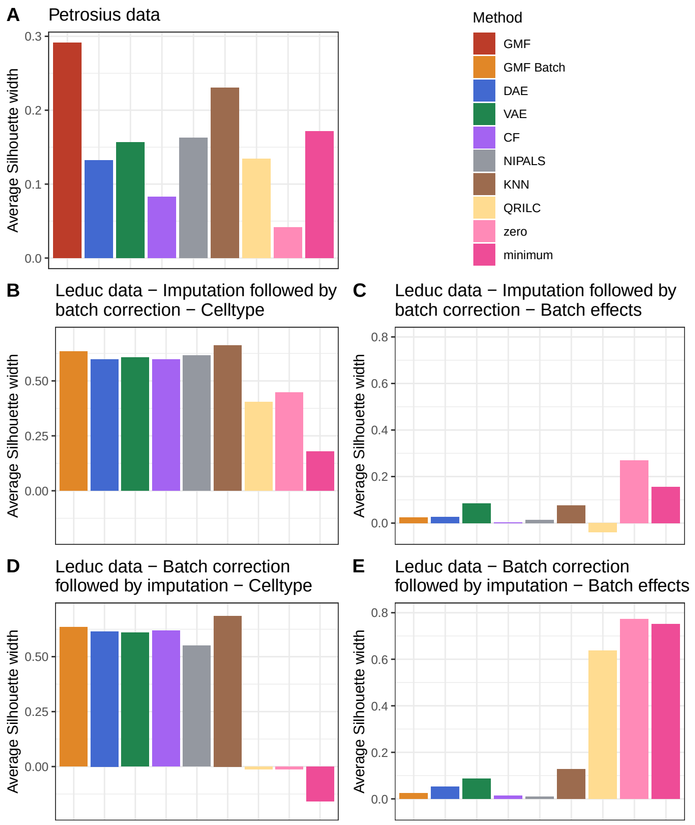

Supp. Fig. 8: Average silhouette width (ASW) on the first two principal components for A) The Petrosius dataset [4] and B-E) the Leduc dataset [3]. Panels A, B and D show ASW for different cell types, with higher ASW indicating better separation between different cell types, therefore resulting in better clustering. Panel C and E show mean ASW for batch evaluated in each cell type separately. Here, lower ASW indicates less separation of different batches within each cell type, thus indicating better clustering, as cells from the same batch should not cluster together after batch correction. omicsGMF (GMF) and omicsGMF while correcting for known batch effects (GMF Batch) directly infer the first two principal components, while the other methods perform imputation followed by PCA (Panel A), imputation followed by batch correction and PCA (Panel B and C) and batch correction followed by imputation and PCA (Panel D and E).

Our response: We disagree with this comment: In our manuscript, the data were preprocessed and normalized prior to running the imputation tools and we already showed the results for PCA upon imputation and batch correction in Supplementary

Figure 6 of our original submission. Moreover, for imputation prior to batch correction and dimensionality reduction, we already show that the imputation performance is suboptimal (see results for the Leduc data in the section where we evaluate the performance of the imputation - Supplementary Figure 14 of the previous submission). We now also obtained new results for workflows that perform batch correction prior to imputation (Leduc dataset). These clearly demonstrate that our unified approach in omicsGMF outperforms ad hoc pipelines employing state-of-the-art imputation methods, whether applied before or after batch correction. We also would like to stress that we already clearly showed the advantage of our omicsGMF tool over existing imputation methods when prioritising differentially abundant proteins in the first version of our manuscript. It should also be noted that, Unlike the manuscripts of the state-of-the-art methods (Li et al. , 2024 and Webel et al., 2024), we use two well known spike-in studies with known ground truth on "DEPs", which is the gold standard in the field.

Moreover, in the revised version of our manuscript, we included novel results on workflows that perform batch correction prior to imputation:

- Paragraph 3 of section "Dimensionality reduction and visualization of proteomics data":

  omicsGMF can also directly account for known covariates, such as batch effects, which we illustrate for the TMT-labeled Leduc dataset (Fig. 3, Panel B; Supplementary Fig. 5). Indeed, Leduc et al. multiplexed up to 14 cells in each TMT run. Upon correcting for the run effect, omicsGMF uncovers additional biological variability corresponding to a previously reported subpopulation of melanoma cells [3]. Imputation of missing values followed by PCA cannot directly control for the technical run-to-run variability, resulting in a dimensionality reduction where the first two latent variables are mainly driven by this strong batch effect. Hence, conventional PCA-based workflows with imputation methods require an

additional step for batch removal prior or post imputation. NIPALS, however, which conceptually does not require imputation of missing values, also cannot account for known batch effects, and therefore suffers from the same caveats. When using a more complicated multi-step workflow that sequentially performs imputation, batch correction, and PCA, similar visualizations as with omicsGMF can be obtained for CF, DAE, VAE, NIPALS and KNN, while QRILC, zero, and minimum imputation clearly display imputation artifacts in their low dimensional visualizations (Supplementary Fig. 6). Similar results are also obtained when performing batch correction prior to imputation for DAE, VAE, CF, NIPALS and KNN (Supplementary Fig. 7). However, small differences remain present, and even large differences are obtained in the visualization of QRILC, zero and minimum imputation. This showcases the benefit of using a single algorithm to tackle imputation and batch effect removal simultaneously, avoiding an arbitrary choice of the sequence in which each of these tasks are performed in current state-of-the-art multi-step workflows.

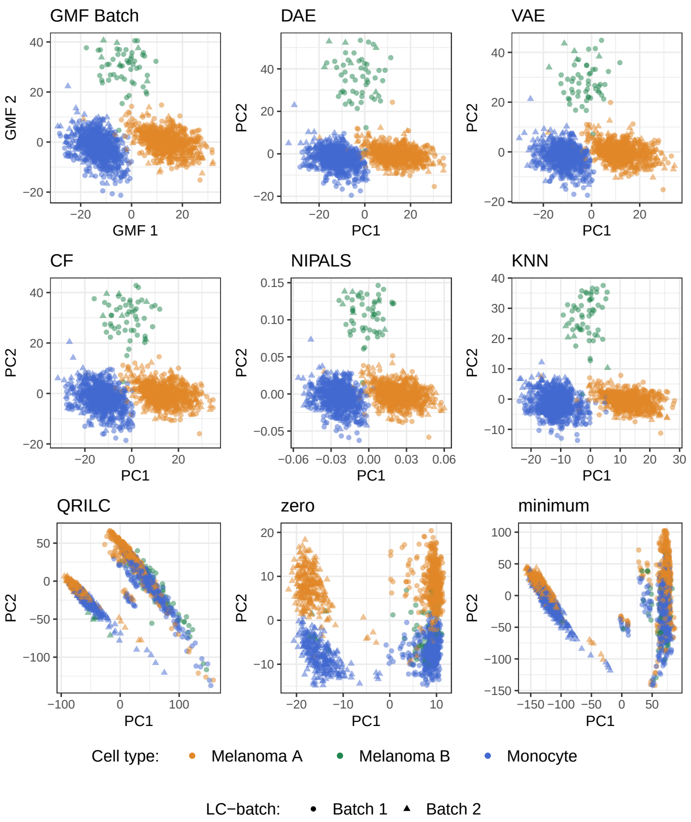

Supp. Fig. 7: Low-dimensional visualization of the Leduc data [3] after batch-correction, colored by cell type. omicsGMF directly accounts for known batch effects. The other visualizations are obtained by sequentially performing batch-correction using linear regression, imputation of missing values using DAE, VAE, CF, NIPALS, KNN, QRILC, and minimum prior to PCA.

- Paragraph 3 of section "Imputation of missing values":

  On the TMT-labeled Leduc dataset (Supplementary Fig. 19 and 20), omicsGMF with batch correction demonstrates better performance compared to all other imputation methods. This finding suggests that when complex technical artifacts are present, effective imputation of missing values requires explicit adjustment for these effects, and this for both MCAR and MNAR data. Note that, even though CF, DAE, and VAE were not designed for TMT-labeled data, they nonetheless outperform conventional imputation methods KNN, QRILC, zero, and minimum imputation. Interestingly, omicsGMF with batch correction remains the top performing workflow even when comparing it to workflows that adopt batch correction prior to imputation with state-of-the-art tools (Supplementary Figs. 21 and 22). This clearly shows the advantage of omicsGMF's strategy to simultaneously perform imputation of missing values with batch correction.

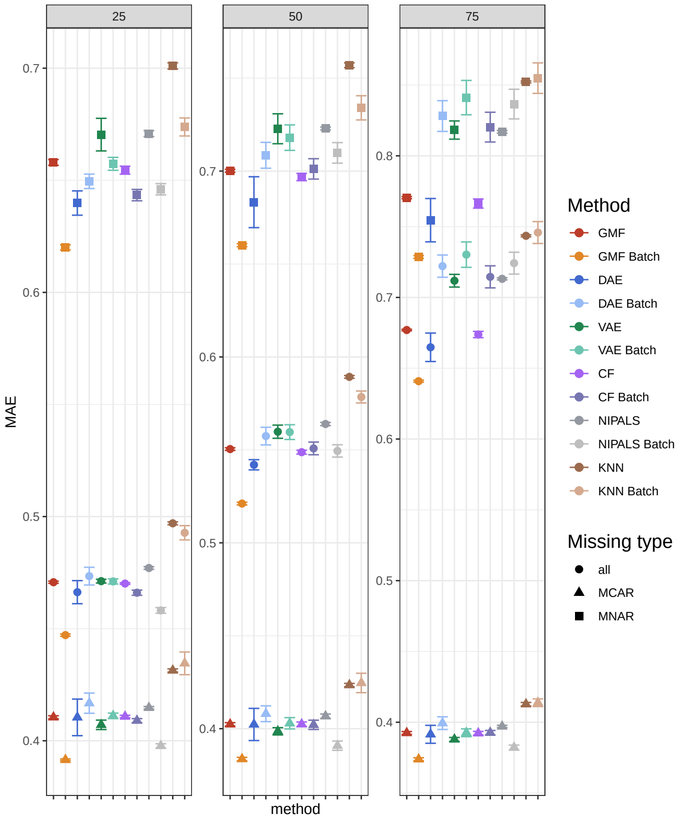

Supp. Fig. 21: Same figure as Supp. Fig 19, but with additional workflows in which batch effects were removed prior to imputation by linear regression including 142 dummy variables indicating TMT-run, except for omicsGMF that performs batch correction internally. These additional workflows are named with 'Batch'.

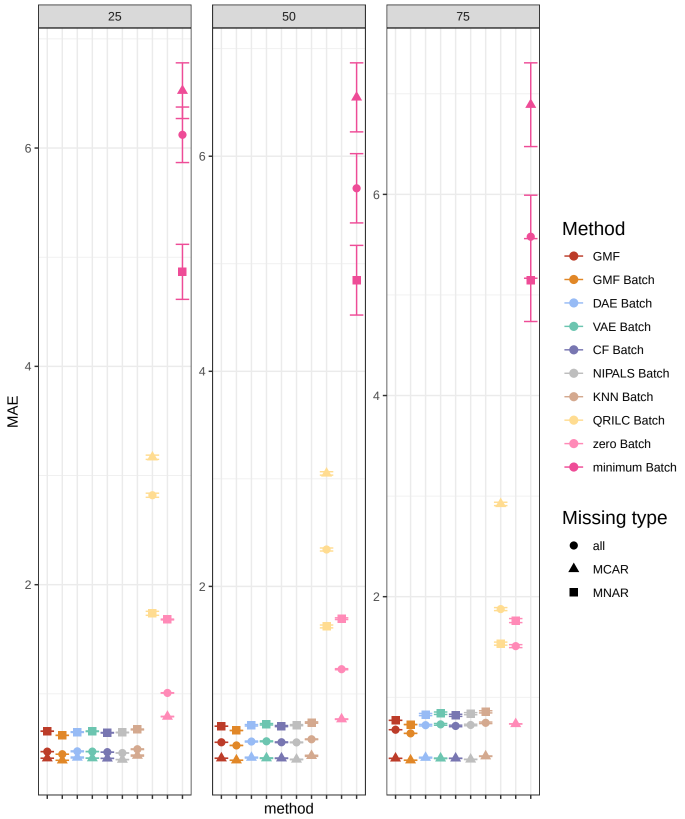

Supp. Fig. 22: Similar figure as Supp. Fig 21, but with QRILC, zero and minimum imputation.

Reviewer 3 - Comment 3

"Limited Relevance to Proteomic Analysis Goals: Proteomic studies primarily aim to identify biologically significant biomolecules for diagnostic or therapeutic applications or dissecting molecular basis. The manuscript's focus on cell and sample clustering, without exploring actionable biomolecular insights, limits its relevance to the proteomics field. Incorporating a comparative analysis of DEPs identified by omicsGMF against those reported in prior studies would be essential to demonstrate the tool's practical value."

Our response: We do not agree with this comment: In the manuscript, we never claim that omicsGMF is developed for diagnostic or therapeutic applications. More importantly, the reviewer seems to have overlooked the entire section on differential analysis in the results where we clearly show the advantage of our omicsGMF tool over existing imputation methods when prioritising "DEPs". Particularly, we use two well-known spike-in studies with known ground truth on "DEPs", which is the gold standard in the field.

Reviewer 3 - Comment 4

"Minor comments

Lack of Statistical Significance in Comparisons: The manuscript does not provide clear indicators of statistical significance, such as the distribution of mean absolute error (MAE) for imputed values across methods. Including statistical tests (e.g., t-tests or ANOVA) to compare omicsGMF's performance with other methods would strengthen the analysis."

Our response: We do not agree with this comment: We use multiple simulations and real spike-in studies to illustrate the performance gain of our method over existing methods using (a) mean absolute error plots to evaluate the imputation performance, and (b) sensitivity - false discovery proportion plots, which are, in fact, the gold

standard in the field. We feel that formal statistical tests to compare the performance are irrelevant in this context. Indeed, with such formal statistical tests we could easily obtain results that are highly statistically significant by simply increasing the number of simulations. Hence, the diagnostic plots as presented in the manuscript are much more representative of actual performance.

Reviewer 3 - Comment 5

"Handling of Leduc Dataset Issues: Previous work has reported low identification and quantification rates in certain batches of the Leduc dataset. The manuscript does not clarify whether these problematic batches were excluded or if consistent effects were observed across all batches. Addressing this would enhance the robustness of the analysis."

Our response: We did not exclude problematic batches. As described in the methods section, we excluded cells based on quality metrics reproducing what has been performed in the original study. We confirm that identification rates are inconsistent between batches (Supplementary Fig. 1B) and that there are strong quantitative batch effects (Fig.S5). We showed that batch effects are correctly removed, irrespective of the number of missing values. Moreover, for the Petrosius dataset (Supplementary Fig. 3), we convincingly showed that the latent factors for omicsGMF suffer less from missing values than the state-of-the-art workflows.

Reviewer 3 - Comment 6

"Need for Benchmark Datasets: To validate the applicability of omicsGMF, the authors should consider using well-characterized datasets with known protein expression ratios across species. Such datasets are widely available and would provide a stronger basis for evaluating the tool's performance."

Our response: The Reviewer seems to have overlooked that the entire section on differential analysis builds on two well-characterized spike-in studies: the CPTAC

study where 48 human UPS proteins were spiked at different concentration in a yeast background, and the study of Shen et al. where E. coli was mixed at different concentrations in a human background.

Reviewer #4

"The article title 'omicsGMF: a multi-tool for dimensionality reduction, batch correction and imputation applied to bulk- and single cell proteomics data' was an interesting read presenting valuable ideas. In general, I am confident the article could pass the revision process, but - maybe unsurprisingly - some clarification and/or corrections might still be needed.

More detailed comments per section, subsection or figure below."

Our response: We thank the Reviewer for their positive evaluation of our manuscript and constructive comments and suggestions, which we carefully addressed in our revised manuscript.

Reviewer 4 - Comment 1

"L58-L61: I guess both sentences will be w.r.t to the publication in [7]?"

Our Response: Indeed, we have adapted the citation as requested.

Nevertheless, these approaches suffer from numerical instability and high computational complexity, making them unsuitable for large-scale SCP datasets ~~[7]~~. Moreover, they are unable to effectively correct for known batch effects, further restricting their practical applicability in proteomics [7].

Reviewer 4 - Comment 2

"L81: 'primarily designed for label-free bulk proteomics with data-dependent acquisition': As I see it most imputation methods are applicable to most matrices. The focus is maybe less in design, but in relevance. TMT without batches has close to zero missing values and data-dependent has a higher share than data-independent. Nonetheless it is good to also consider other setups in benchmarking. So potentially rephrase a bit or go into detail about the particularities of multi-batch TMT setups w.r.t to missing values."

Our response:

We agree with the Reviewer that the sentence focused too much on the design. The approach by Webel et al. has been designed on LFQ data. Although this approach could be amenable for labeled experiments, this was not demonstrated by the authors. Gianetti et al. built a model with assumptions that only hold for LFQ data and do not extend to labelled experiments, namely the model does not account for peptide identifications that are more consistent within a TMT run compared to across runs. The paragraph now reads as follows:

Moreover, the prevalence of MCAR and MNAR is largely influenced by the data acquisition strategy, as well as by the specific characteristics of precursors, peptides and proteins, making the imputation process particularly challenging. To address this issue, more sophisticated methods have been developed, leveraging machine learning approaches [13] or explicitly estimating the proportions of MCAR and MNAR values to inform the selection of an appropriate imputation strategy [14]. However, these methods are primarily ~~designed~~ demonstrated for label-free bulk proteomics with data-dependent acquisition strategies, whereas labeled approaches and data-independent acquisition techniques are much more commonly employed in LSP and SCP to enhance proteome coverage.

Reviewer 4 - Comment 3

"L90-L91: One could argue that good tools would create higher-level functions connecting different analysis steps (e.g. imputation and batch correction). Your method makes this unnecessary. In this paragraph you already discuss results which I find too early. Mentioning it in the introduction also leads to having Figure1 to be the PCAs, which is unfortunate as Figure2 rather introduces omicsGMF (and would be the first one to be shown on any social media link)."

Our response: We thank the reviewer for this suggestion. However, we would like to state that Figure 1 does not show novel results, but rather shows the problems

of state-of-the-art methods in literature. Therefore, we think this figure is useful for readers to quickly understand the problems related to these tools, which we try to solve with omicsGMF.

Reviewer 4 - Comment 4

"L95: I remember to have seen scVI. It lacks the interpretability of omicsGMF but I thought it does aim for similar things. You could look at the tooling around it to see if this is relevant (scvi-tools). I am not using it nor am I a developer of it. If you know it, I would rather be interested as an user if this is something you considered and discarded – or if it goes into the same direction. If irrelevant, ignore this hint. https://docs.scvi-tools.org/en/stable/user_guide/index.html"

Our response: We thank the reviewer for this suggestion. scVI is developed for single-cell RNA-sequencing data, and only allows for negative binomial, zero-inflated negative binomial, and Poisson distributions. The other modules in scvi-tools also do not tackle the needs of mass-spectrometry based proteomics data, i.e., using a Gaussian likelihood to model the log-transformed intensities. totalVI for example can include CITE-Seq data, but these protein data are also counts in nature. Furthermore, the VAE module of PIMMS should produce similar results as an adapted scVI module for Gaussian data, as scVI also uses a variational autoencoder. Taken together, we decided to not include scvi-tools in the analysis, as the use of count distributions is not sensible for intensity data, and similar results should be obtained by the VAE module of PIMMS when developing an adapted version of scVI to include the Gaussian distribution.

Reviewer 4 - Comment 5

"Results

I like the SVD-like approach including factors of known covariance resulting in a linear (in parameters) and interpretable method.

Figure2: ‘feature-specific effect’: is this correct? As B is sample batch-effects. Assuming it is correct, a bit more explanations would help me. Is it p sample specific covariates (batch-effects) and q feature-specific covariates (not-sure what this normally is called). Then there is ‘cell-by-peptide’ and ‘cell-specific’. I would suggest to keep the overview a bit more generic as in principle it could be anything (with normally distributed featues?) If you have an example of feature-specific covariates from any of the three datasets included into the paper, this would be great (probably you have it somewhere, but at the time of writing this review I do not recall it)”

Our response: We thank the reviewer for this suggestion. We have now explicitly used the following terminology: sample-level covariates, feature-specific parameters, sample-level parameters and feature-level covariates in Figure 2 to avoid confusion. Figure 2 now looks as follows:

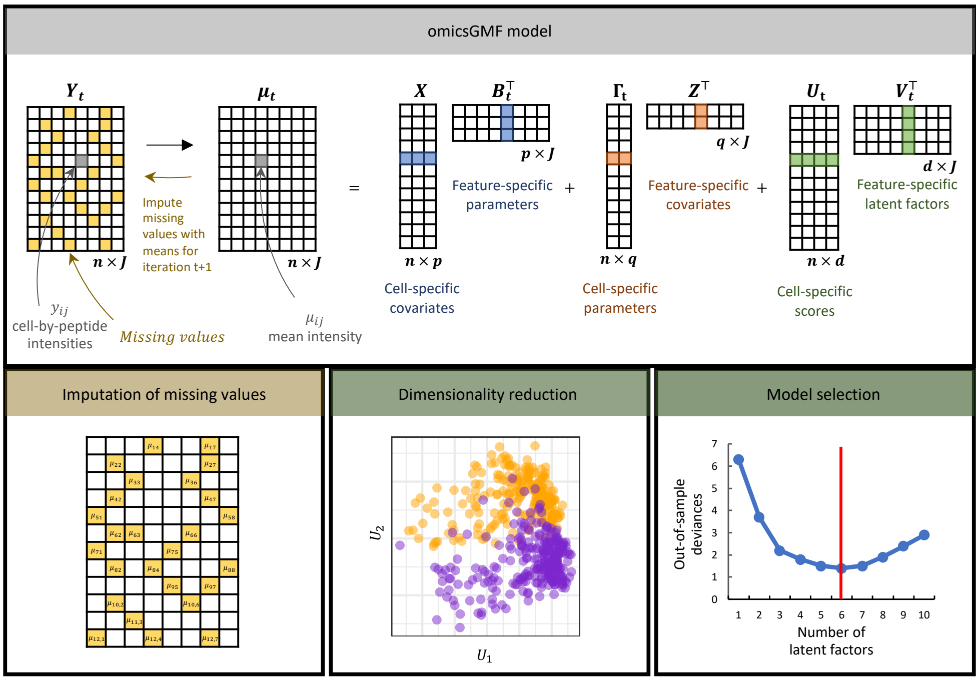

Fig. 2: Schematic overview of the omicsGMF model for the Gaussian Model family. $\mathbf{Y}$ is modeled in function of known sample-level covariates $\mathbf{X}$, feature-level covariates $\mathbf{Z}$, latent factors $\mathbf{U}$ and their loadings $\mathbf{V}$. omicsGMF iteratively estimates the parameters $\boldsymbol{B}$, $\boldsymbol{\Gamma}$, $\mathbf{U}$ and $\mathbf{V}$. omicsGMF addresses missing values by re-imputing them in each iteration with their current mean $\boldsymbol{\mu}_t$. The latent factors have a similar interpretation as principal components upon correcting for known covariates, and thus allow for dimensionality reduction and visualization. omicsGMF can also provide the imputed values upon convergence, which are useful for downstream applications. Furthermore, omicsGMF allows for model selection that can guide the user for choosing the number of latent factors and known covariates to be included in the model. More details can be found in the Methods section.

Reviewer 4 - Comment 6

"L146: you mention iterative and later (L154) multi-step. At first this confused me, but one is w.r.t to the fitting algorithm (iterative regression) and the other more conceptual. I would argue that in L154 the reader does not yet know what 'multi-step' workflows are. I am not sure how to solve this, but reading your method I would say that it is imputation+linear batch correction with different tools and scProtein (which you are not able to use in the end). Could you maybe refer to the methods or a table here to make it clearer? I would maybe just swap the sentences (starting in L154 and L155): your method can do both and avoids higher-level workflows combining two things."

Our response: We have adapted paragraph 2 of the "Results" section as follows:

omicsGMF estimates all the unobserved parameters $\mathbf{U}$*,* $\boldsymbol{B}$*,* $\boldsymbol{\Gamma}$ *and* $\mathbf{V}$*. Since both* $\mathbf{U}$ *and* $\mathbf{V}$ *are unknown, a closed-form solution is not available. Therefore, omicsGMF uses iterative regression of* $\mathbf{Y}$ *on* $[\mathbf{X}, \hat{\boldsymbol{\Gamma}}, \hat{\mathbf{U}}]$ *followed by regression of* $\mathbf{Y}$ *on* $[\hat{\boldsymbol{B}}, \mathbf{Z}, \hat{\mathbf{V}}]$ *to estimate all the parameters. Here, the hat notation indicates the current estimate of the corresponding parameters. Due to the extensive number of missing values, these linear regressions are not possible without imputing such unobserved data. Therefore, omicsGMF iteratively imputes the missing values with the estimated mean of the prior iteration. The advantages are threefold. First, the imputed values account for known sample- and feature-level covariates, as well as the latent structure, which implies imputation under a Missingness At Random (MAR) assumption conditional on both known covariates and information in other features through latent factors. ~~resulting~~ This results in accurate imputed intensities even when dealing with significant technical effects. Second, it performs imputation, correction for technical effects and the estimation of latent factors simultaneously, therefore avoiding complicated multi-step workflows that sequentially perform these individual tasks. ~~Second, it avoids complicated multi-step workflows and performs imputation and correction for technical~~*

~~*effects simultaneously.*~~ *Therefore, the user does not suffer from different results that rely on the order in which batch correction and imputation are conducted [17, 24]. Third, it is an all-in-one workflow that uses a set of linear equations* ~~*maximizes a Gaussian likelihood*~~*, rendering its parameter estimates, latent variables, and imputed values easily interpretable. Additionally, the latent covariates* **U** *used for dimensionality reduction and visualization have a similar interpretation to conventional PCA.*

Reviewer 4 - Comment 7

"L157-L160: Is the workflow not rather a set of linear equations. Gaussian like-likelihood can be optimized by a variational autoencoder loss-function. The strong point is that is similar to PCA and thus interpretable?"

Our response: We thank the reviewer for their suggestion, and agree to adapt the text in the revised manuscript to emphasize its use of linear equations.

- Paragraph 7 of the introduction:

  As a result, omicsGMF effectively addresses the three primary challenges of dimensionality reduction in the LSP and SCP contexts, while preserving the interpretability of conventional PCA by using a set of linear equations ~~*maximizing a Gaussian likelihood function*~~.

- Paragraph 2 of the "Results" section

  Third, it is an all-in-one workflow that uses a set of linear equations ~~*maximizes a Gaussian likelihood*~~*, rendering its parameter estimates, latent variables, and imputed values easily interpretable. Additionally, the latent covariates* **U** *used for dimensionality reduction and visualization have a similar interpretation to conventional PCA.*

Reviewer 4 - Comment 8

"L174: in [19]?"

Our response: We have updated this sentence accordingly.

Reviewer 4 - Comment 9

"L175-L187: Is this not rather for the last part of the introduction where you outline you detailed plan (or part of the section summarizing the following sections?)"

Our response: We know that this information is also given in the last part of the Introduction. However, we feel that it is useful for the reader to partially repeat this here to give them an overview of the structure of the result section.

Reviewer 4 - Comment 10

"In this section the other multi-step workflows (L200-L206) are defined (by the authors). I would mainly suggest that the comparison in Supplementary Fig. 6 are better suited for comparison to omicsGMF. If lucky imputation, batch correction and PCA are three function calls (probably this is over optimistic). For me adding batch correction to the comparison says that omcisGMF is a good method as it makes it easier to handle all the aspects without knowing several tools."

Our response: We thank the reviewer for this suggestion. However, we think that retaining the original Figure, in which no batch correction is done for the other methods, delivers the message more clearly:

- It clearly illustrates the importance of batch correction in the analysis of such datasets.
- Next, we introduce two additional workflows based on existing methods: 1) batch correction prior to imputation and PCA; and 2) imputation prior to batch correction and PCA.
- As 1) and 2) give different results, it is not clear which one should be included in the main paper.
- omicsGMF does not suffer from this choice as it tackles these problems simultaneously.

Therefore, including the original plot without batch correction in the main paper allows for an easy discussion of the benefits of omicsGMF, while we still explicitly state that similar results can be obtained by using more complex workflows that perform each of these tasks sequentially.

We have also added the workflows with batch correction prior to imputation, and have changed paragraph 3 of section "Dimensionality reduction and visualization of proteomics data" and Supplementary Information as follows:

omicsGMF can also directly account for known covariates, such as batch effects, which we illustrate for the TMT-labeled Leduc dataset (Fig. 3, Panel B; Supplementary Fig. 5). Indeed, Leduc et al. multiplexed up to 14 cells in each TMT run. Upon correcting for the run effect, omicsGMF uncovers additional biological variability corresponding to a previously reported subpopulation of melanoma cells [3]. Imputation of missing values followed by PCA cannot directly control for the technical run-to-run variability, resulting in a dimensionality reduction where the first two latent variables are mainly driven by this strong batch effect. Hence, conventional PCA-based workflows with imputation methods require an additional step for batch removal prior or post imputation. NIPALS, however, which conceptually does not require imputation of missing values, also cannot account for known batch effects, and therefore suffers from the same caveats. When using a more complicated multi-step workflow that sequentially performs imputation, batch correction, and PCA, similar visualizations as with omicsGMF can be obtained for CF, DAE, VAE, NIPALS and KNN, while QRILC, zero, and minimum imputation clearly display imputation artifacts in their low dimensional visualizations (Supplementary Fig. 6). Similar results are also obtained when performing batch correction prior to imputation for DAE, VAE, CF, NIPALS and KNN (Supplementary Fig. 7). However, small differences remain present, and even large differences are obtained in the visualization of QRILC, zero, and minimum imputation. This showcases the benefit of using a single algorithm to tackle imputation and batch

effect removal simultaneously, avoiding an arbitrary choice of the sequence in which each of these tasks are performed in current state-of-the-art multi-step workflows.

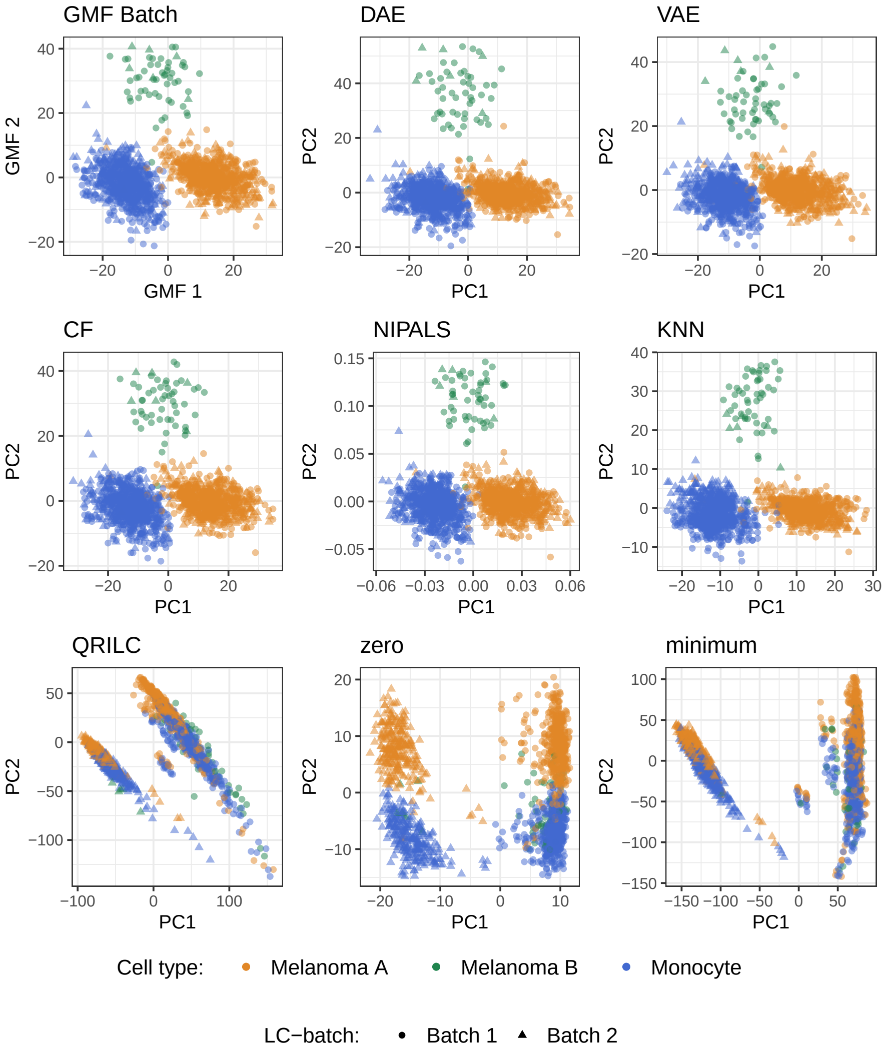

Supp. Fig. 7: Low-dimensional visualization of the Leduc data [3] after batch-correction, colored by cell type. omicsGMF directly accounts for known batch effects. The other visualizations are obtained by sequentially performing batch-correction using linear regression, imputation of missing values using DAE, VAE, CF, NIPALS, KNN, QRILC, and minimum prior to PCA.

Reviewer 4 - Comment 11

"L245: Here PSMs are mentioned, in Figure2 and elsewhere peptides are mentioned. This just makes we wonder what kind of data was used? Were PSMs, precursors (sequence, PTMs and charge, potentially summarizing several PSMs) or peptides (sequence plus PTMs) used?"

Our response: We thank the reviewer for pointing out this mistake. Precursor-level data were used for both the Petrosius and Leduc datasets, while peptide-level data were used for the CPTAC and E. coli datasets. We have now corrected the manuscript as follows:

- Paragraph 4 of the Introduction:

  Moreover, the prevalence of MCAR and MNAR is largely influenced by the data acquisition strategy, as well as by the specific characteristics of precursors, peptides and proteins, making the imputation process particularly challenging.

- Paragraph 1 from section "Dimensionality reduction and visualization of proteomics data":

  This is done for the label-free single-cell Petrosius dataset [4] consisting of 525 cells and 4435 ~~peptides~~precursors with 58.5% missing values, the TMT-labeled single-cell Leduc dataset [3] consisting of 1508 cells and 6280 ~~peptides~~precursors with 61.8% missing values, and the bulk label-free CPTAC study [16] where 48 human UPS proteins were spiked in at five different concentrations in a yeast proteome background.

- Paragraph 5 of section "Dimensionality reduction and visualization of proteomics data":

  However, omicsGMF is still the only method that can distinguish between the most challenging conditions A and condition B. Indeed, many missing values for the differentially abundant UPS ~~PSMs~~ peptides occur in these conditions due to their low spike-in concentrations.

- The caption of Figure 4:

  Cross-validation with omicsGMF allows for comprehensive selection of the number of latent factors d in **U** *for dimensionality reduction. Each panel shows the mean of the out-of-sample deviances of masked values (precursors for Petrosius and Leduc, and peptides for CPTAC) over three cross-validation folds in function of the number of latent factors d included in the model. In each fold, 30% of the values are masked for out-of-sample prediction. Panel A shows the cross-validation results for the Petrosius dataset [4] with and without accounting for the treatment effect (one dummy variable). Panel B shows the cross-validation results for the Leduc dataset [3], with and without correcting for the known batch-effect associated to multiplexing cells in the same run (142 dummy variables). Panel C shows the cross-validation results for the CPTAC data [16], considering all three labs. Results are shown for omicsGMF without known covariates, accounting for the lab effects (two dummy variables), and accounting for both the lab (two dummy variables) and spike-in concentration effects (four dummy variables).*

- Paragraph 3 of section "Differential analysis:

  Interestingly, the bias in fold change estimates for spike-in UPS proteins disappears when only the observed ~~PSM~~ peptide intensities are used in the msqrob2 analysis.

- Paragraph 7 of section "Differential analysis":

  Interestingly, the bias in fold change estimates for spike-in UPS proteins disappears when only the observed ~~PSM~~ peptide intensities are used in the msqrob2 analysis.

- The "Data" subsection in the "Methods":

  This manuscript builds its results from two single cell and two bulk proteomics datasets that were acquired with either TMT-labeling or label-free quantification.

First, the Petrosius dataset [4] originates from a label-free SCP experiment on mouse embryonic stem cells incubated in two distinct conditions i.e., a serum-free 2i condition (m2i) containing cytokine LIF with inhibitors for the MEK and GSK3 pathways, and a serum condition (m15) containing cytokine LIF, only. Data were acquired using an orbitrap Eclipse Tribrid mass spectrometer operated in data independent acquisition mode. The MS data were analyzed by the authors using Spectronaut v17 [32]. We started from the Skyline table containing measured intensities and feature annotations at the precursor level, i.e. a feature is defined by the combination of the peptide sequence, its modification and its ion charge. We performed quality control by removing cells with fewer than 750 detected ~~peptides~~precursors or a median log2-intensity lower than log2(7). Features with more than 90% missing values were also removed. The detected intensities were log2-transformed for variance-stabilization, and the intensities of each cell were centered with the median log2-intensity of the corresponding cell. The resulting dataset contains 525 cells with 4435 detected ~~peptides~~precursors, of which 58.5% of the values are missing.

Second, the Leduc dataset [3] was acquired using pSCoPE technology, which relies on TMT labeling and a prioritized data dependent acquisition strategy carried out by a Thermo Scientific Q-Exactive mass spectrometer. The MS data were quantified and identified using MaxQuant version 1.6.17 [33]. We used the precursor table generated by the authors. In this dataset, precursors are defined by the peptide sequence and the ion charge. We performed quality control by filtering cells with fewer than 750 detected peptides or a median coefficient of variation greater than 0.5, calculated using the medianCVperCell function of the scp [15] package. ~~Peptides~~Precursors with more than 90% missing values were removed, resulting in a dataset with 6280 ~~peptides~~precursors for 1508 cells and a total of 61.8% missingness. The intensities were log2-transformed for variance-stabilization, and the

log2-intensities of each cell are centered using the median log2-intensity of the corresponding cell.

Third, data from an interlaboratory bulk label-free proteomics spike-in study of the CPTAC consortium was used [16]. Forty-eight human proteins were spiked in a background of yeast proteins at 5 different concentrations ($A = 0.25 fmol/\mu l$, $B = 0.76 fmol/\mu l$, $C = 2.2 fmol/\mu l$, $D = 6.7 fmol/\mu l$ and $E = 20 fmol/\mu l$) and three samples of each concentration were sent to three different labs resulting in data from forty-five different MS-runs. Hence, the ground truth on differential abundance is known, which is useful to assess the imputation of missing values for differential (UPS) and non-differential proteins (Yeast), and how the different imputation methods affects the downstream differential abundance analyses. The data were searched and quantified according to [25]. We started from the peptide table generated by MaxQuant, so every feature is defined by the peptide sequence. ~~Again, p~~Peptides with more than 90% missing values were removed, resulting in a total of 10105 peptides remaining from 1477 yeast proteins and 520 peptides from 44 human proteins and 42.5% missing values.

Finally, we used Shen dataset [27], another bulk proteomics label-free benchmarking effort consisting of E. coli proteins mixed in five different weight ratio's in a background of the human reference proteome (in wt/wt percentage: a = 3%, b = 4.5%, c = 6%, d = 7.5 % and e = 9%). After normalization and filtering peptides with more than 90% missing values, the dataset consists of 28944 peptides from 756 E. coli proteins and 3954 human proteins with 18.8% of the values missing. The data originates from twenty MS-runs acquiring four samples from each spike-in condition.

Reviewer 4 - Comment 12

"L257: The competing workflows are defined by the authors, if I get this correctly. So I would rather say 'other workflows'. Same in L277 'existing methods': There are not really existing methods which do this in one step?"

Our response: We thank the reviewer for their suggestion. We agree that existing methods cannot do this in one step, and change the manuscript correspondingly:

- Paragraph 1 of section "Model selection by cross-validation":

  The other ~~competing~~ workflows also do not infer or provide guidelines on the number of latent factors for imputation nor for downstream applications, such as clustering, UMAP and t-SNE visualizations, or for correction for unknown batch effects and confounders, amongst others.

- Paragraph 2 of section "Model selection by cross-validation":

  Nevertheless, the application clearly shows the strengths and relevance of omicsGMF in practice: omicsGMF captures this variability better than the other workflows ~~existing methods~~ in an unsupervised analysis and can effectively remove it from the analysis for applications that aim to cluster cells upon removal of the treatment or batch effect.

Reviewer 4 - Comment 13

"L264: This now goes to a new figure, which might warrant a new section. I would maybe explain which matrix dimension this refers to in Figure2 and state the matrix symbol. Then Figure4 is also easier to understand."

Our response: We thank the reviewer for this suggestion. We now include a new section header called "Model selection by cross-validation". Further, we have changed the manuscript in paragraph 2 of this section to include a reference to the matrices defined in Figure 2 as follows:

Fig. 4 shows results for omicsGMF-based selection of both the number of relevant latent variables d in **U** *and the relevant known covariates* **X** *and* **Z** *with cross-validation.*

Reviewer 4 - Comment 14

"Figure4: I would mention the matrix which dimensions are optimized. Are all mentionend covariates batch-effects? And I would like to know what the values are (could be elsewhere, but I guess peptides or PSMs?)"

Our response: We thank the reviewer for their suggestion. We addressed their comment as follows: 1) We changed the caption to include the matrix dimensions. 2) We would like to note that the caption in the previous version of the manuscript already mentioned what the covariates indicate, i.e., treatment effect for Petrosius, batch effect for Leduc and lab effects (and spike-in concentration) for CPTAC. 3) We now also include information on the feature-level to which the out-of-sample deviances refer. Further, we refer to Comment 11 for the changes related to the data types. The new caption in the revised version of the manuscript now reads as follows:

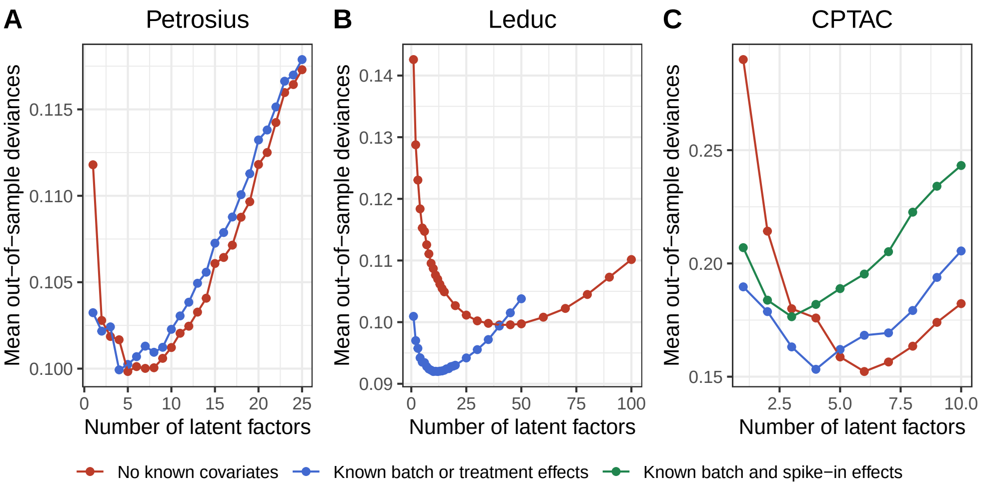

Fig. 4: Cross-validation with omicsGMF allows for comprehensive selection of the number of latent factors d in U for dimensionality reduction. Each panel shows the mean of the out-of-sample deviances of masked values (precursors for Petrosius and Leduc, and peptides for CPTAC) over three cross-validation folds in function of the number of latent factors d included in the model. In each fold, 30% of the values are masked for out-of-sample prediction. Panel A shows the cross-validation results for the Petrosius dataset [4] with and without accounting for the treatment effect (one dummy variable). Panel B shows the cross-validation results for the Leduc dataset [3], with and without correcting for the known batch-effect associated to multiplexing cells in the same run (142 dummy variables). Panel C shows the cross-validation results for the CPTAC data [16], considering all three labs. Results are shown for omicsGMF without known covariates, accounting for the lab effects (two dummy variables), and accounting for both the lab (two dummy variables) and spike-in concentration effects (four dummy variables).

Reviewer 4 - Comment 15

"L298-L304: A linear model captures a linear increase in concentrations. That is good, but maybe also not the most challenging experimental setup in that case. I am not sure what the discussion on Figure4, Panel C tells me for using it in practice. Is it bad? Will I see batch effects in my plots?"

Our response: Although this might not be the most challenging experimental setup, we want to stress that this linear increase is not present for all features, but only for a limited number of spike-in features, which omicsGMF captures on its own. Furthermore, it is interesting that one, by looking at the cross-validation plots, can observe that 4 dummy variables can be captured in a model with a single continuous variable. This reduces the degrees of freedom used by the model, and showed superior performance for out-of-sample imputation error. Therefore, the cross-validation can help users to determine which known covariates to include in the model, as well as the number of latent factors. Note that this model selection is indeed optimized with imputation in mind, and not the visualization. Therefore, the optimal model for imputation might not remove batch effects from the visualization. We have made this explicit in paragraph 4 of section "Model selection by cross-validation":

The results for the CPTAC study are particularly insightful (Fig. 4, panel C). Indeed, cross-validation for an omicsGMF analysis conducted without incorporation of known covariates identifies six latent variables. However, the introduction of the "Lab" variable in the omicsGMF model, produces a cross-validation score that is on par, albeit with only four latent variables: a reduction which matches with the inclusion of two categorical dummy variables representing Lab 2 and Lab 3, as Lab 1 serves as the reference group. Upon incorporating an additional categorical variable for spike-in condition, the optimal number of latent variables is further reduced to three. Interestingly, omicsGMF that correct for the lab variable seems to capture the variability of the spike-in condition with a single additional latent variable, although incorporating

the spike-in condition explicitly in the omicsGMF model included four additional categorical dummy variables. This suggests that omicsGMF detects the linear increase in UPS protein levels across conditions using a single, continuous, latent variable, which aligns with the actual experimental design. However, the cross-validation performance that includes the spike-in concentration is clearly inferior compared to omicsGMF models that exclude known covariates or account only for a batch effect associated with lab, probably because the more generous model with a factor for spike-in concentration suffers from overfitting. Note that the cross-validation optimizes the out-of-sample imputation deviances, which is desirable when using omicsGMF for imputation. However, this might not be the desired model for visualization, for example when one wants to visualize the clustering in the CPTAC study upon removal of the lab effect. Hence, the user should explicitly specify known effects when they want to remove these sources of variability from the latent factors prior to visualization and/or downstream clustering tasks.

Reviewer 4 - Comment 16

"Imputation of missing values

So just to be sure. The error plots are w.r.t to the log2 and mean standardised intensities (to zero mean)? I normally would expect nonnegative values in Figure5."

Our response: All data are indeed log2-transformed and normalized with median centering, hence observed and imputed values that are negative are not unexpected, which is confirmed in Figure 5. Note, however, that Figure 5 is not an error plot, but compares the distributions of observed intensities versus the distribution of imputed intensities for real missing values of spike-in peptides in the different spike-in conditions. Hence, no masking of observed values is done and the CPTAC data was used as is. The error plots for simulated missing values are shown in Supplementary Figures

17-29, and these show the mean absolute error, which cannot be negative by construction. To make this more clear, we have made small changes to the text that refers to Figure 5, and also changed the y-axis from 'Intensity' to 'log2-normalized intensities':

Interestingly, the CPTAC study, with known ground truth for different spike-in protein concentrations ~~differentially abundant proteins~~, also allows the evaluation of the imputations for missing values in the original dataset, i.e., without masking values. Two things should be observed related to these spike-in concentrations: missing values for peptides of spike-in proteins are expected (a) to be imputed generally lower than observed values within a specific spike-in concentration as missing values are likely present due to poor ionisation efficiency and/or low abundance of the protein, and (b) follow the trend in the spike-in concentrations of the observed peptides of spike-in proteins. Fig. 5 presents the distribution of observed values, and, imputed log2-normalized peptide intensities for spike-in proteins, stratified by spike-in condition.~~, alongside those by different imputation methods.~~ This analysis excludes data from Lab 1, which is affected by ionization issues. Notably, omicsGMF successfully captures the concentration gradient in the missing values for UPS proteins, whereas other methods fail to recover this trend, particularly in the low spike-in conditions A, B, and C. Additionally, omicsGMF systematically imputes missing values lower than the observed ones, suggesting that missingness is driven by low abundance combined with lower ionization efficiency for specific peptide species. In contrast, other methods tend to shift imputed values toward higher intensities, sometimes exceeding those observed in conditions A, B, and even C, indicating difficulties in accurately imputing MNAR data. For non-differentially abundant yeast proteins, the imputed value distributions remain similar across conditions but are consistently lower than their observed counterparts (Supplementary Fig. 30).

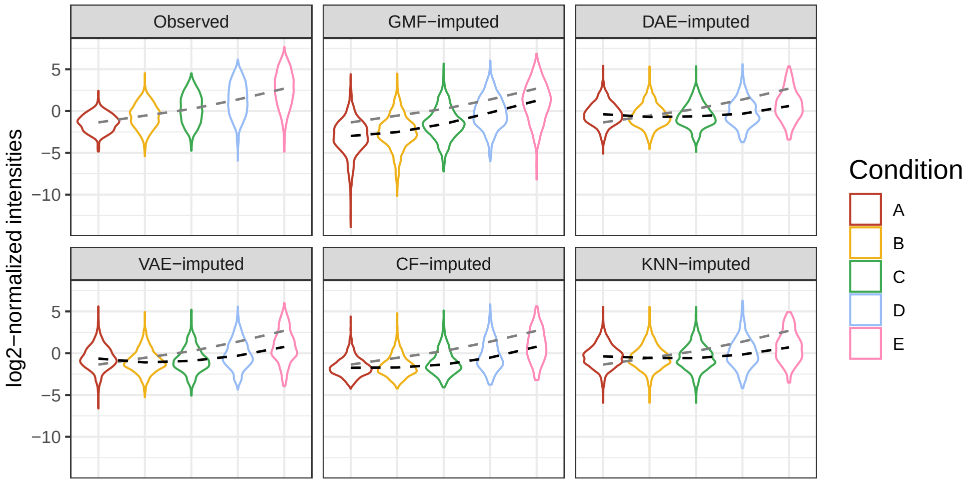

Fig. 5: omicsGMF imputation accounts for missingness due to low abundance. Distributions of log2-normalized peptide intensities from human spike-in proteins are shown in function of the spike-in condition for the original CPTAC data without masking values and excluding Lab 1 that suffered from ionization issues. The first panel shows the distribution of observed values, and the other panels show the distributions of imputed intensities of the missing values by omicsGMF, DAE, VAE, CF and KNN imputation respectively. The grey dashed line represents the median of the observed values, and the black dashed line the median of the imputed values.

Reviewer 4 - Comment 17

"The paragraph does not contain the results in numbers which makes it a bit hard to compare and assess. Was there a specific reason for that? I would argue that if you say a method is better, then this should be quantified a tiny bit."

Our response: We agree with the reviewer that referring to the quantitative values of MAEs could increase clarity. However, there are 11 Supplementary Figures that discuss the mean absolute errors, each figure including at least 7 methods and 3 different error quantities across the different missing data natures (MNAR, MCAR, all). Therefore, including all quantities in the main manuscript would make it very dense for the reader. Instead, we now provide Supplementary Data that includes all the quantitative results.

In paragraph 1 of section "Imputation of missing values" we now refer to the Supplementary Data as follows:

To evaluate imputation performance, we simulate missingness both completely at random (MCAR) and missing not at random (MNAR) due to low abundance (see Methods). The mean absolute error (MAE) between the imputed and original values is then assessed across ten different random seeds, considering scenarios where 25%, 50%, and 75% of the masked values are attributed to low abundance. The MAEs for all simulations are available in Supplementary Data.

Reviewer 4 - Comment 18

"L314-L322: Maybe refer to the methods? The comparison is w.r.t to the default parameters of the other methods (as stated in the methods)?"

Our response We thank the reviewer for this suggestion. We have adapted paragraph 2 of section "Imputation of missing values" as follows:

On the label-free Petrosius dataset (Supplementary Fig. 17 and 18), omicsGMF demonstrates superior imputation performance compared to PIMMS neural network-based approaches DAE, VAE and CF, as well as to traditional methods such as NIPALS, KNN, QRILC, and zero or minimum imputation with their default settings (see Methods).

Reviewer 4 - Comment 19

"L343-L347. Could you elaborate how the ground trough in differential analysis is related to the imputation comparisons? You state that you simulated missing values, which you use as ground truth in any comparison, or do I misunderstand that? The median of all imputed peptides is used to illustrate the concentration ratio?"

Our response: We feel that the Reviewer is misunderstanding the results shown in Supplementary Figures 17-29, and the differences with the results shown in Figure 5. The Supplementary Figures show the mean absolute errors results across all datasets with simulated missing values. The results in Figure 5 are entirely based on real CPTAC data as is. It shows the observed log2-normalized intensities of peptides from spike-in proteins in the CPTAC study across various spike-in concentrations in the first panel, and the imputed values for the peptide intensities of spike-in proteins that really went missing in the study; each panel showing the imputed values using a different method. The ground truth knowledge of the CPTAC study, i.e, the spike-in concentrations of human UPS peptides, allows us to compare the imputed values across these different spike-in concentrations. As missing values are expected to occur due to low abundance, specifically for the low spike-in concentrations, the imputed intensity distributions are expected to be lower than the observed ones. Furthermore, the distribution of the imputed values for the spike-in peptides is expected to be centered around lower values for the lower spike-in concentrations. This pattern is indeed observed for omicsGMF and is lacking for the competing methods. We have

now included this more explicitly in the manuscript, as discussed in Reviewer 4 - Comment 16.

Reviewer 4 - Comment 20

"L350-L354: GMF-imputation seems to have some out-of-distribution predictions which can also lead to artefacts in differential analysis (Figure5, values way below -5). Is there any explanation why this has no effect on the differential analysis? Then, aren't these simulated missing values which are used for this comparison? If it is non-simulated missing values, then this comparison is still not yet showing ground-truth I would argue. You compare between 5 conditions one PSM or peptide. If in one condition some values are missing you want to see it correctly imputed? The overall distribution can be a proxy, but in the end you would need to show maybe the ratio's distribution using different imputation methods? (I would need to think more about it)."

Our response: For the out-of-distribution imputations: As missing values can be due to low abundance it is not unexpected that missing values for the spike-in peptides are imputed lower than the observed values. Note, however, that extremely small values are undesired. This is observed for one specific imputed value, which is imputed with a value near -13. The other values, however, nicely follow a distribution that is shifted to lower levels than the observed intensities. Moreover, our simulation studies show lower mean average error on simulated missing values. Interestingly, our method allows for ridge penalties in the estimation process, which should make the estimates more robust to outliers. However, this would require additional tuning and hyperparameter optimization, which we did not pursue in our manuscript.

Further, our previous responses to the section 'Imputation of missing values' have clarified the differences between the simulation results with mean average errors in Supplementary figures, and the distribution plots of the imputed missing values that were observed in the real dataset for spike-in peptides and are shown in Figure 5.

Reviewer 4 - Comment 21

"Here you start talking about human proteins, whereas before the analysis was on PSM or peptides level (see question). Did you continue your analysis on the peptide level (which is probably the most sensible)? How many peptides of the 48 spike-in proteins did you observe? So what is 'original non-imputed data'?"

Our Response: In this section we assess differential abundance at the protein-level by using peptide level models that directly models the filtered log2 normalized peptide intensities of all peptides that belong to the same protein. The models include an effect for the spike-in condition, lab effect, random sample effects and random peptide specific effects. Hence, the treatment effect has the interpretation of the $\log_2$ fold-change at the protein level while correcting for peptide specific effects. A total of 520 UPS peptides were observed, with varying missing proportions per spike-in condition (see Supplementary Figure 10 in the manuscript - included below).

We have adapted the first paragraph of the section "Differential analysis" as follows:

In a differential analysis using MS-based proteomics data, researchers are typically interested in prioritizing differentially abundant proteins from the quantified precursor or peptide-level intensities. Here, we will evaluate differential abundance analysis upon imputation using the CPTAC experiment, for which the ground truth is known on differential abundance (DA). Indeed, a mix of 48 digested human UPS proteins are spiked at different concentrations in a constant background of 1477 digested non-DA yeast proteins. All workflows start from the observed log2-normalized peptide-level data quantified with MaxQuant. The msqrob2 [25, 26] tool is used for differential analysis. msqrob2 can directly prioritize DA proteins by jointly modeling the peptide-level data protein by protein (see Methods). Particularly, a linear mixed model is used with fixed effects for spike-in condition and lab, and random effects for peptide species and sample. Multiple data analysis workflows are considered: a workflow using the data as is

without imputing missing data, and workflows upon imputation with omicsGMF or the state-of-the-art imputation tools. ~~*The CPTAC experiment provides a ground truth for differential abundance (DA) analysis, where 48 human UPS DA proteins are spiked at different concentrations alongside 1,477 non-DA yeast proteins. DA was inferred using msqrob2 [25, 26], incorporating a main effect for treatment and a block effect for lab, both on the original non-imputed data and on data processed with different imputation methods.*~~

Further, we have adapted paragraph 4 of the "Data" subsection in the "Methods" as follows to include the number of peptides for human proteins:

Third, data from an interlaboratory bulk label-free proteomics spike-in study of the CPTAC consortium was used [16]. Forty-eight human proteins were spiked in a background of yeast proteins at 5 different concentrations (A $= 0.25 fmol/\mu l$*, B* $= 0.76 fmol/\mu l$*, C* $= 2.2 fmol/\mu l$*, D* $= 6.7 fmol/\mu l$ *and E* $= 20 fmol/\mu l$*) and three samples of each concentration were sent to three different labs resulting in data from forty-five different MS-runs. Hence, the ground truth on differential abundance is known, which is useful to assess the imputation of missing values for differential (UPS) and non-differential proteins (Yeast), and how the different imputation methods affects the downstream differential abundance analyses. The data were searched and quantified according to [25]. We started from the peptide table generated by MaxQuant, so every feature is defined by the peptide sequence.* ~~*Again, p*~~*Peptides with more than 90% missing values were removed, resulting in a total of 10105 peptides remaining from 1477 yeast proteins and 520 peptides from 44 human proteins and 42.5% missing values.*

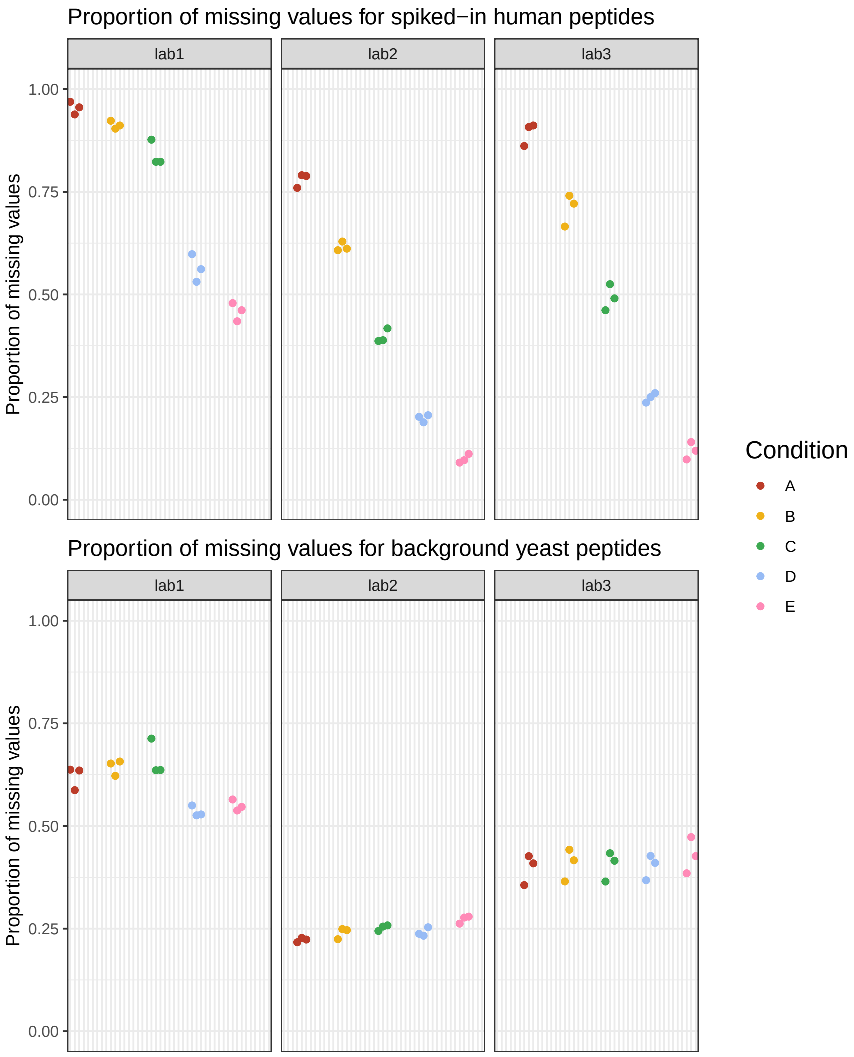

Supp. Fig. 10: Proportion of missing values for each sample of the complete CPTAC dataset [16], for spike-in human peptides (top) and the background yeast peptides (bottom) respectively. The proportion of missing values is represented in function of the spike-in concentration, and is stratified per lab.

Reviewer 4 - Comment 22

"I did not find the a method section for the details on this comparison, which would help me understand the mathematical setup here (simple t-test with multiple-testing correction on 'corrected' data?)"

Our response: We thank the reviewer for their suggestion. We have now included additional details on the msqrob2 analysis in the Methods section and refer to that in the first paragraph of section Differential Analysis in the results (see our answer to Reviewer 4 - Comment 21):

Differential abundance analysis

The differential abundance analysis is typically done at the protein level for which we use the msqrob2 tool [25, 26]. msqrob2 has peptide-level workflows that can directly start from all log2-transformed and normalized peptide level intensities while conducting inference at the protein level. Hence, it does not require a separate aggregation step of peptide-level data in protein-level abundance values. Specifically, a peptide-based msqrob2 workflow models all peptides that belong to the same protein with a single linear mixed model that contains dummy variables to estimate the effect of the spike-in condition (treatment of interest) while correcting for peptide specific effects, lab effects, and the correlation of peptide intensities from the same protein in the same sample. Specifically, the spike-in condition and the lab effect were fitted with a fixed effect and the peptide and sample effects with random effects [25]. Hence, the parameters for the spike-in condition immediately estimate protein-level log2 fold-changes with respect to the reference spike-in condition while correcting for lab effects, peptide effects and addressing the hierarchical correlation in the data. Upon parameter estimation, statistical inference was done on contrasts of the parameters for the spike-in effect to prioritize differentially abundant proteins across all pairwise comparisons.

Reviewer 4 - Comment 23

"L379-L389: Same as before. Quantitative comparison without any numbers. It's w.r.t to AUC values? Figure5: In panel A (B versus A) the KNN and DAE curves look off. Is it due to an artefact, an error or is there a performance reason? Is FDP the same as FDR as you mention both. (I looked it up: In theory this might be different, but here it is the same due to the empirical nature?)"

Our response:

- FDR vs FDP: The FDR and FDP are indeed two distinct concepts. The FDP is the false discovery proportion: number of false positives divided by the number of positive features (true positives + false positives) that is returned and this can only be calculated in practice when the ground truth is known, e.g. in a spike-in study or in simulation studies. The FDR or the false discovery rate is the expected FDP, which we can estimate in real studies if the null distribution of the test statistic is known, e.g. with the Benjamini-Hochberg method. In simulation studies we can get an idea on the empirical FDR by constructing many simulated datasets and averaging the FDP over the different simulations. For a spike-in study, only one repeat of a ground truth study is available so we can only calculate the empirical FDP of a method and cannot assess its FDR empirically. However, an empirical FDP that is way larger than the nominal FDR threshold is indicative for methods that do not provide a good FDR control.
- TPR-FDP curves vs ROC curves. In the past, benchmarking papers often included ROC curves where the true positive rate (TPR) or sensitivity (number of true positives returned / (number of true positives + number of false negatives) is plotted against the false positive rate (FPR) or 1- specificity (Number of false positives / (number of false positives + true negatives)). For these plots the area under the curve is often used as a global measure for the performance. Indeed, it gives an idea on the average sensitivity over all possible FPR's. However, this

is not very useful in practice as researchers are typically not interested in the sensitivity of lists with significant features that contain a lot of false positives. Indeed, in a high throughput context one typically tries to control the FDR, or the expected fraction of false positives in the toplists that are returned. Hence, benchmarking papers have shifted from ROC curves to TPR-FDR curves if simulation studies are used or TPR-FDP curves for spike-in studies as these better reflect the properties of interest in practice. Moreover, for practical applications we are again mainly interested in the beginning of the plot, i.e. at low FDRs or FDPs as researchers typically do not want to accumulate too many false positives in the results that they return. Hence, an AUC value to summarize the performance again would focus on a large part of the curve that is not of interest for practitioners. Moreover, AUC values can also not be used in this context because the empirical FDP (FDR) is not increasing monotonically when the size of the returned toplist is increased. Indeed, if the significance threshold is lowered and if a false positive is added to the list, the FDP will increase. However, the empirical FDP can then decrease again when the next number of features in the list are true positives. Even though we feel that the TPR-FDP curves tell the full story, we now also provide a table with quantitative results: i.e. the number of false positives and true positives and the empirical FDP at an FDR cutoff of 1%, 5% and 10%.

- The curves of DAE and KNN are indeed off. This, because these methods immediately start to accumulate false positives in their top lists, even before a UPS protein is returned as differentially abundant. Hence, the FDP starts at 1 while the TPR is 0. The FDP then decreases as a number of UPS proteins is picked up.

We now explicitly state that DAE and KNN return false positives before any UPS proteins in paragraph 2 of "Differential analysis":

We first focus on the analysis using data from Labs 2 and 3. Fig. 6, panel A shows that only the workflows using non-imputed data and omicsGMF-imputed data provide reliable inference between the two lowest spike-in conditions, A and B. Other imputation methods generate numerous false positives in their top-ranked results, or even return false positives before any UPS protein is called differentially abundant (DAE, KNN). This outcome aligns with previous observations in Fig. 5, where most methods impute similar values for missing UPS peptides across these conditions. For comparisons between B vs. C and A vs. C, CF's performance improves considerably, but still falls short of omicsGMF, which is also confirmed by the number of retrieved true positives at the 5% false discovery proportion (Supplementary Fig. 32). Meanwhile, other state-of-the-art imputation methods continue to struggle with reliable DA inference. As the comparisons shift to higher spike-in concentrations, the performance of all methods improves. However, omicsGMF remains the top performer in the majority of the comparisons (Supplementary Fig. 33).

Further, we now include a reference to the Methods section for TPR and FDP in Figure 6, accompanied by a novel subsection in the Methods section called "Performance metrics":

Performance metrics

Multiple performance metrics were used throughout this manuscript. Here we discuss how each of these are computed.

Average silhouette width: *The silhouette is a measure of how similar an observation is to its cluster compared to these of other clusters, i.e., the silhouette s_i for a cell i that is assigned to cluster C_k is computed with the cluster package, and is defined by:*

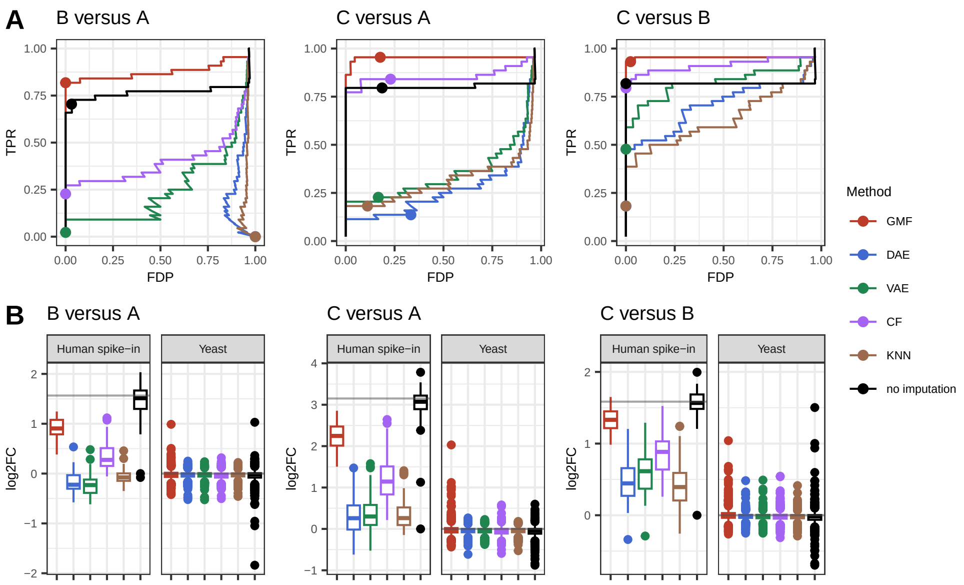

Fig. 6: omicsGMF imputation leads to better downstream differential abundance analysis. Performance evaluation of differential abundance analyses using msqrob2 [25, 26] on the CPTAC dataset [16]. Results for the comparisons between the lowest spike-in concentrations B versus A, C versus A and C versus B are shown. Data from Lab 1 are excluded due to ionization issues. Human UPS proteins are differentially spiked between the conditions, with yeast background proteins as true negative control. Panel A shows the true positive rate (TPR, see Methods) in function of the false discovery proportion (FDP, see Methods). The dots on each curve represent working points when the FDR level is set at the nominal 5% level. Panel B shows the estimated log2 fold changes (FC) by msqrob2 for both human spike-in proteins, and for reference yeast proteins. The grey line indicates the known log2 FC.

$$s_i = \frac{b_i - a_i}{max(b_i, a_i)}, \tag{1}$$

with b_i the mean distance between cell i and all other cells in the nearest other cluster C_l with $l \neq k$, and a_i the mean distance between cell i and all other cells in the same cluster k. A value near 1 therefore represent great separation between the different clusters, while a value near 0 represents complete mixing of the clusters.

Mean absolute error: *The mean absolute error is used to evaluate imputation errors. Observed values are masked to NA, followed by imputation by different algorithms. Then, the mean average error is defined by:*

$$MAE = \frac{\sum_{(i,j)\in M} |y_{ij} - \hat{y}_{ij}|}{n_M}, \tag{2}$$

with y_{ij} the observed value of sample i for feature j, $\hat{y}_{ij}$ the imputed value of sample i for feature j, M the set of all masked values and n_M the number of masked values.

True positive rate: *The true positive rate of a set of proteins returned by the methods with a p-value lower then a certain threshold is defined by:*

$$TPR = \frac{true\ positives}{true\ positives + false\ negatives}, \tag{3}$$

False discovery proportion: *The false discovery proportion of a set of proteins returned by the methods with a p-value lower then a certain threshold is defined by:*

$$FDP = \frac{false\ positives}{true\ positives + false\ positives}, \tag{4}$$

Reviewer 4 - Comment 24

"Figure6: So no-imputation is best, but accounting for the ionization makes omicsGMF better? So no imputation and no batch correction leads already to the best results? How many missing values were present in the peptides associated with the 48 human proteins?"

Our response: We do not agree that no-imputation is necessarily best. It depends on the preference of the user. Indeed, using a workflow without imputation leads to better estimates of the log2 fold-changes (Panel B). However, it goes hand in hand with a lower sensitivity as less UPS proteins can be picked up as DA. Hence, if a researcher

prefers a higher sensitivity while tolerating some bias in the reported fold changes, they can be better off with a workflow upon imputation with omicsGMF. Indeed, the sensitivity upon omicsGMF imputation is better for all comparisons. Interestingly, the bias in the fold changes estimates upon omicsGMF imputation also remains reasonably small. This is clearly stated in the manuscript:

Fig. 6, Panel B, and Supplementary Fig. 34 show that all imputation methods result in an underestimation of the fold change for spike-in UPS proteins. However, omicsGMF is the only approach that produces fold change estimates reasonably close to the ground truth, confirming that other methods struggle to correctly impute missing values due to low abundance. Interestingly, the bias in fold change estimates for spike-in UPS proteins disappears when only the observed PSM intensities are used in the msqrob2 analysis. However, this comes at the cost of reduced sensitivity as seen in Panel A of Fig. 6, because fewer data points are available for many spike-in proteins due to missingness.

For the proportion of missing values, we would like to refer the Reviewer to Supplementary Figure 10 in the manuscript, also shown in Reviewer 4 - Comment 21.

Reviewer 4 - Comment 25

"In general for results: It would be nice to have some number in the text to not go estimate these from the figures. This also helps me at least double check if the text and the figures align (kind of a double check). Do you support Supplementary Data for all the Figures (at some point I guess you will need to anyways)? "

Our response:

We now include Supplementary Data for all quantitative comparisons, i.e., the average silhouette widths, out-of-sample deviances of the cross-validation, the mean average imputation errors, and the FDP control at an FDR of 1%, 5% and 10%. Further, all figures can be reproduced with our github repository for the paper.

Reviewer 4 - Comment 26

"In general the results have a lot of interesting ideas and I have to admit that I did not check all supplementary figures.

Discussion

Brief and neat. Maybe add your SGD mini-batch approach. Similar to neural networks, but with a clearer linear parameter setup. So you gain computational efficiency without compromising on interpretability."

Our response: We thank the reviewer for their suggestion. We did not find a paragraph to smoothly incorporate the computational efficiency in the discussion. However, we have now included scalability benchmarks in the results section and Supplementary information, which clearly show the benefit of using our SGD mini-batch approach. Paragraph 7 of section "Dimensionality reduction and visualization of proteomics data" now reads as follows:

Note further that omicsGMF reduces to a kind of expectation-maximization (EM) PCA when no sample or feature-level covariates are included. However, conventional EM-PCA struggles to converge, probably due to the excessive number of missing values (Supplementary Fig. 13). Furthermore, EM-PCA is very slow, and does not scale well to larger datasets. Neither does NIPALS, which performs PCA by ignoring missing values. In contrast, omicsGMF solves these problems by optimizing its parameters through an efficient adaptive stochastic gradient descent algorithm. This makes omicsGMF fast and scalable to large datasets, especially with regards to PCA-like algorithms EM-PCA and NIPALS, and to imputation methods such as DAE, VAE and CF (Supplementary Fig. 14).

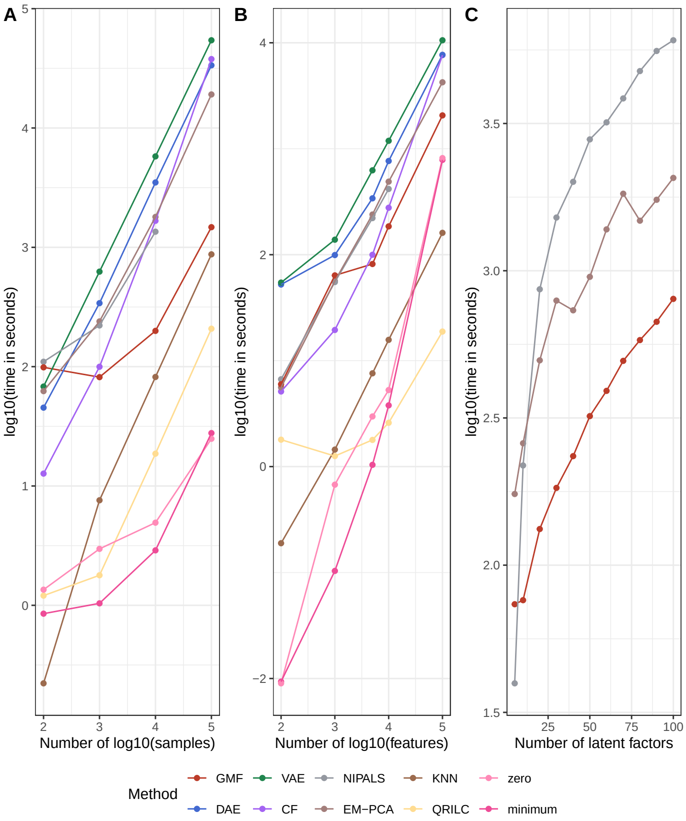

Supp. Fig. 14: Benchmark of computational time (in log10 of the number of seconds) in function of the number of samples (Panel A), the number of features (Panel B) and the number of latent factors for PCA-like algorithms (Panel C). Thousand samples are used in Panel B and C, five thousand features are used in Panel A and C, and ten latent factors are used in Panel A and B. omicsGMF is much faster compared to PCA-like algorithms that can deal with missing values (NIPALS and EM-PCA), and is faster than PIMMS imputation (DAE, VAE and CF) on large datasets. Methods like KNN, QRILC, zero and minimum imputation are faster than omicsGMF, but do not account for the correlation structure in the data. NIPALS could not return results in Panel A for 100000 samples.

Reviewer 4 - Comment 27

"Methods

L497: 'can contain' is there a default? I guess the user does not need to care about that?"

Our response: Indeed, the intercepts are included by default. We have changed first paragraph of subsection "omicsGMF Gaussian matrix factorization model" in the "Methods" of the revised manuscript as follows:

with $\mathbf{X}$ *a* $n \times p$ *matrix with known sample-level covariates, such as batch or cell type or spike-in concentration, and* $\boldsymbol{B}$ *a matrix with its corresponding* $J \times p$ *regression parameters,* $\mathbf{Z}$ *a* $J \times q$ *matrix with known feature specific covariates, and* $\boldsymbol{\Gamma}$ *a matrix with its corresponding* $n \times q$ *regression parameters. Both* $\mathbf{X}$ *and* $\mathbf{Z}$ *by default* ~~*can*~~ *contain a column of ones, corresponding to gene-specific and sample-specific intercept, respectively. The* $n \times d$ *latent covariate matrix* $\mathbf{U}$ *and its* $J \times d$ *loading matrix* $\mathbf{V}$ *explain the residual variation that are not captured by the known covariates.*

Reviewer 4 - Comment 28

"Data

L658-L659. So you mention peptides for yeast, but not for humans. Is there a reason? So it is still not clear how you get from peptides to proteins (or you do not) for the differential analysis. In the end I would expect to perform the DA analysis on peptides as the visualizations are based on peptides, right?"

Our response: We thank the reviewer for pointing this out. We refer the reviewer to the answers on Reviewer 4 - Comment 21 and Reviewer 4 - 22 that provide the changes implemented to discuss how msqrob2 uses peptide-level intensities to infer differentially abundant proteins. Further, we have now included the number of peptides for human proteins in Paragraph 4 of the "Data" subsection in the "Methods" as follows:

Third, data from an interlaboratory bulk label-free proteomics spike-in study of the CPTAC consortium was used [16]. Forty-eight human proteins were spiked in a background of yeast proteins at 5 different concentrations ($A = 0.25 fmol/\mu l$, $B = 0.76 fmol/\mu l$, $C = 2.2 fmol/\mu l$, $D = 6.7 fmol/\mu l$ and $E = 20 fmol/\mu l$) and three samples of each concentration were sent to three different labs resulting in data from forty-five different MS-runs. Hence, the ground truth on differential abundance is known, which is useful to assess the imputation of missing values for differential (UPS) and non-differential proteins (Yeast), and how the different imputation methods affects the downstream differential abundance analyses. The data were searched and quantified according to [25]. We started from the peptide table generated by MaxQuant, so every feature is defined by the peptide sequence. ~~Again, p~~Peptides with more than 90% missing values were removed, resulting in a total of 10105 peptides remaining from 1477 yeast proteins and 520 peptides from 44 human proteins and 42.5% missing values.

Reviewer 4 - Comment 29

"Well structured repository, although not executed version of all the files exist. The makefile should allow to re-run it fairly easy for the authors and it gives hints on the structure.

I have not seen how to set up the dependencies myself. A docker image could help or some details instructions. But I would only invest the time if the article goes further and their answers are convincing."

Our Response: We have included a renv file to setup the proper libraries and dependencies.

References

[1] Bader, J.M., Geyer, P.E., Müller, J.B., Strauss, M.T., Koch, M., Leypoldt, F., Koertvelyessy, P., Bittner, D., Schipke, C.G., Incesoy, E.I., Peters, O., Deigendesch, N., Simons, M., Jensen, M.K., Zetterberg, H., Mann, M.: Proteome profiling in cerebrospinal fluid reveals novel biomarkers of alzheimer's disease. Molecular Systems Biology **16**(6), 9356 (2020) https://doi.org/10.15252/msb.20199356 https://www.embopress.org/doi/pdf/10.15252/msb.20199356

[2] Niu, L., Thiele, M., Geyer, P.E., Rasmussen, D.N., Webel, H.E., Santos, A., Gupta, R., Meier, F., Strauss, M., Kjaergaard, M., Lindvig, K., Jacobsen, S., Rasmussen, S., Hansen, T., Krag, A., Mann, M.: Noninvasive proteomic biomarkers for alcohol-related liver disease. Nature Medicine **28**(6), 1277–1287 (2022) https://doi.org/10.1038/s41591-022-01850-y

[3] Leduc, A., Huffman, R.G., Cantlon, J., Khan, S., Slavov, N.: Exploring functional protein covariation across single cells using npop. Genome Biology **23**(1), 261 (2022) https://doi.org/10.1186/s13059-022-02817-5

[4] Petrosius, V., Aragon-Fernandez, P., Üresin, N., Kovacs, G., Phlairaharn, T., Furtwängler, B., Op De Beeck, J., Skovbakke, S.L., Goletz, S., Thomsen, S.F., Keller, U.a.d., Natarajan, K.N., Porse, B.T., Schoof, E.M.: Exploration of cell state heterogeneity using single-cell proteomics through sensitivity-tailored data-independent acquisition. Nature Communications **14**(1), 5910 (2023) https://doi.org/10.1038/s41467-023-41602-1

[5] Vanderaa, C., Gatto, L.: Replication of single-cell proteomics data reveals important computational challenges. Expert Review of Proteomics **18**(10), 835–843 (2021) https://doi.org/10.1080/14789450.2021.1988571 https://doi.org/10.1080/14789450.2021.1988571. PMID: 34602016

[6] Guldberg, S.M., Okholm, T.L.H., McCarthy, E.E., Spitzer, M.H.: Computational methods for single-cell proteomics. Annual Review of Biomedical Data Science **6**(Volume 6, 2023), 47–71 (2023) https://doi.org/10.1146/annurev-biodatasci-020422-050255

[7] Vanderaa, C., Gatto, L.: Revisiting the thorny issue of missing values in single-cell proteomics. Journal of Proteome Research **22**(9), 2775–2784 (2023) https://doi.org/10.1021/acs.jproteome.3c00227

[8] Estimation of Principal Components and Related Models by Iterative Least Squares. Multivariate Analysis, pp. 391–420. NYAcademic Press (1966)

[9] Stacklies, W., Redestig, H., Scholz, M., Walther, D., Selbig, J.: pcaMethods—a bioconductor package providing PCA methods for incomplete data. Bioinformatics **23**(9), 1164–1167 (2007) https://doi.org/10.1093/bioinformatics/btm069 https://academic.oup.com/bioinformatics/article-pdf/23/9/1164/49858899/bioinformatics_23_9_1164.pdf

[10] Roweis, S.: Em algorithms for pca and spca. Advances in neural information processing systems **10** (1997)

[11] Lazar, C., Gatto, L., Ferro, M., Bruley, C., Burger, T.: Accounting for the multiple natures of missing values in label-free quantitative proteomics data sets to compare imputation strategies. Journal of Proteome Research **15**(4), 1116–1125 (2016) https://doi.org/10.1021/acs.jproteome.5b00981

[12] Sticker, A., Goeminne, L., Martens, L., Gevaert, K., Clement, L.: Msqrob takes the missing hurdle: Uniting intensity- and count-based proteomics. Analytical Chemistry **92**(9), 6278–6287 (2020) https://doi.org/10.1021/acs.analchem.9b04375

[13] Webel, H., Niu, L., Nielsen, A.B., Locard-Paulet, M., Mann, M., Jensen, L.J., Rasmussen, S.: Imputation of label-free quantitative mass spectrometry-based proteomics data using self-supervised deep learning. Nature Communications **15**(1), 5405 (2024) https://doi.org/10.1038/s41467-024-48711-5

[14] Gianetto, Q.G., Wieczorek, S., Couté, Y., Burger, T.: A peptide-level multiple imputation strategy accounting for the different natures of missing values in proteomics data. bioRxiv (2020) https://doi.org/10.1101/2020.05.29.122770 https://www.biorxiv.org/content/early/2020/05/30/2020.05.29.122770.full.pdf

[15] Vanderaa, C., Gatto, L.: The current state of single-cell proteomics data analysis. Current Protocols **3**(1), 658 (2023) https://doi.org/10.1002/cpz1.658 https://currentprotocols.onlinelibrary.wiley.com/doi/pdf/10.1002/cpz1.658

[16] Paulovich, A.G., Billheimer, D., Ham, A.-J.L., Vega-Montoto, L., Rudnick, P.A., Tabb, D.L., Wang, P., Blackman, R.K., Bunk, D.M., Cardasis, H.L., Clauser, K.R., Kinsinger, C.R., Schilling, B., Tegeler, T.J., Variyath, A.M., Wang, M., Whiteaker, J.R., Zimmerman, L.J., Fenyo, D., Carr, S.A., Fisher, S.J., Gibson, B.W., Mesri, M., Neubert, T.A., Regnier, F.E., Rodriguez, H., Spiegelman, C., Stein, S.E., Tempst, P., Liebler, D.C.: Interlaboratory study characterizing a yeast performance standard for benchmarking lc-ms platform performance*. Molecular & Cellular Proteomics **9**(2), 242–254 (2010) https://doi.org/10.1074/mcp.M900222-MCP200

[17] Vanderaa, C., Gatto, L.: Scplainer: Using linear models to understand mass spectrometry-based single-cell proteomics data. Genome Biol. **26**(1), 237 (2025)

[18] Li, W., Yang, F., Wang, F., Rong, Y., Liu, L., Wu, B., Zhang, H., Yao, J.: scprotein: a versatile deep graph contrastive learning framework for single-cell proteomics embedding. Nature Methods **21**(4), 623–634 (2024) https://doi.org/

10.1038/s41592-024-02214-9

[19] Castiglione, C., Segers, A., Clement, L., Risso, D.: Stochastic gradient descent estimation of generalized matrix factorization models with application to single-cell RNA sequencing data (2024). https://arxiv.org/abs/2412.20509

[20] Troyanskaya, O., Cantor, M., Sherlock, G., Brown, P., Hastie, T., Tibshirani, R., Botstein, D., Altman, R.B.: Missing value estimation methods for DNA microarrays . Bioinformatics **17**(6), 520–525 (2001) https://doi.org/10.1093/bioinformatics/17.6.520 https://academic.oup.com/bioinformatics/article-pdf/17/6/520/48837104/bioinformatics_17_6_520.pdf

[21] Lazar, C., Burger, T.: imputeLCMD: A Collection of Methods for Left-Censored Missing Data Imputation. (2022). R package version 2.1. https://CRAN.R-project.org/package=imputeLCMD

[22] Risso, D., Ngai, J., Speed, T.P., Dudoit, S.: Normalization of rna-seq data using factor analysis of control genes or samples. Nature Biotechnology **32**(9), 896–902 (2014) https://doi.org/10.1038/nbt.2931

[23] Risso, D., Perraudeau, F., Gribkova, S., Dudoit, S., Vert, J.-P.: A general and flexible method for signal extraction from single-cell rna-seq data. Nature communications **9**(1), 284 (2018)

[24] Voß, H., Schlumbohm, S., Barwikowski, P., Wurlitzer, M., Dottermusch, M., Neumann, P., Schlüter, H., Neumann, J.E., Krisp, C.: Harmonizr enables data harmonization across independent proteomic datasets with appropriate handling of missing values. Nature Communications **13**(1), 3523 (2022) https://doi.org/10.1038/s41467-022-31007-x

[25] Goeminne, L.E., Gevaert, K., Clement, L.: Peptide-level robust ridge regression

improves estimation, sensitivity, and specificity in data-dependent quantitative label-free shotgun proteomics*. Molecular & Cellular Proteomics **15**(2), 657–668 (2016) https://doi.org/10.1074/mcp.M115.055897

[26] Sticker, A., Goeminne, L., Martens, L., Clement, L.: Robust summarization and inference in proteome-wide label-free quantification. Molecular & Cellular Proteomics **19**(7), 1209–1219 (2020) https://doi.org/10.1074/mcp.ra119.001624

[27] Shen, X., Shen, S., Li, J., Hu, Q., Nie, L., Tu, C., Wang, X., Poulsen, D.J., Orsburn, B.C., Wang, J., Qu, J.: Ionstar enables high-precision, low-missing-data proteomics quantification in large biological cohorts. Proceedings of the National Academy of Sciences **115**(21), 4767–4776 (2018) https://doi.org/10.1073/pnas.1800541115 https://www.pnas.org/doi/pdf/10.1073/pnas.1800541115

[28] Ahlmann-Eltze, C., Huber, W., Anders, S.: Deep learning-based predictions of gene perturbation effects do not yet outperform simple linear methods. bioRxiv (2024) https://doi.org/10.1101/2024.09.16.613342 https://www.biorxiv.org/content/early/2024/09/21/2024.09.16.613342.full.pdf

[29] Cai, J.-F., Candes, E.J., Shen, Z.: A Singular Value Thresholding Algorithm for Matrix Completion (2008). https://arxiv.org/abs/0810.3286

[30] Mazumder, R., Hastie, T., Tibshirani, R.: Spectral regularization algorithms for learning large incomplete matrices. The Journal of Machine Learning Research **11**, 2287–2322 (2010)

[31] Wang, S., Li, W., Hu, L., Cheng, J., Yang, H., Liu, Y.: Naguider: performing and prioritizing missing value imputations for consistent bottom-up proteomic analyses. Nucleic Acids Research **48**(14), 83–83 (2020)

https://doi.org/10.1093/nar/gkaa498 https://academic.oup.com/nar/article-pdf/48/14/e83/34131162/gkaa498.pdf

[32] Bernhardt, O., Selevsek, N., Gillet, L., Rinner, O., Picotti, P., Aebersold, R., Reiter, L.: Spectronaut: a fast and efficient algorithm for mrm-like processing of data independent acquisition (swath-ms) data. (2014)

[33] Cox, J., Mann, M.: Maxquant enables high peptide identification rates, individualized p.p.b.-range mass accuracies and proteome-wide protein quantification. Nature Biotechnology **26**(12), 1367–1372 (2008) https://doi.org/10.1038/nbt.1511

Point by point rebuttal

Reviewer #1 (Remarks to the Author):

"The authors have sufficiently addressed all my comments, I wish them best of luck with the remaining publication process."

Reviewer #1 (Remarks on code availability):

"Both the code for reproducing results from the paper and the package omicsGMF are available on github and well documented."

Our response: We thank the reviewer for their careful and positive evaluation of our manuscript.

Reviewer #2 (Remarks to the Author):

"*The authors have addressed most of my previous concerns, and the manuscript has been substantially improved. I only have a few remaining suggestions for further clarification and presentation improvement:*

1. In the revised manuscript, the authors have added assumptions regarding how their method handles two types of missing data, which is appreciated. However, the current description is still rather abstract and may not be sufficiently intuitive for readers to follow. In addition, the concept of Missing At Random (MAR) is newly introduced but not explicitly explained, which further affects the clarity of this part. I suggest that the authors revise this part to more clearly and concretely explain what assumptions are made for each type of missingness and how the proposed method handles each case."

Our response: We thank the reviewer for their suggestion. We have adapted both the first part of the Results section, as well as the '*Imputation of missing values*' of the Results section.

The second paragraph of the Results section now includes the following lines:

"Therefore, omicsGMF iteratively imputes the missing values with the estimated mean of the prior iteration. The advantages are threefold. First, the imputed values account for known sample- and feature-level covariates, as well as the latent structure. This implies that imputation is done under the Missingness At Random (MAR) assumption, specifically we consider that the probability of missingness only depends on the observed data,

i.e. it can be explained by the feature type, known sample- and feature-level covariates as well as by information in other features through the latent structure."

The section 'Imputation of missing values' of the Results now reads as follows:

"The final mean estimates obtained from omicsGMF can also be used for the imputation of missing values while accounting for latent factors, as well as for known sample- and feature-level covariates. This imputation strategy assumes missingness at random which assumes that the probability of missingness only depends on the observed data, i.e. it can be explained by the feature type, known sample- and feature-level covariates as well as by information in other features through the latent structure. ~~that can be explained by known covariates and latent structure.~~ Although it does not explicitly assume missingness due to low abundance, which is particularly present when considering data from a single feature (e.g. a precursor or peptide) in isolation, omicsGMF leverages the known covariates and the correlation structure across multiple precursors, peptides and proteins. This joint modelling of features allows to partially explain missingness by low abundance in one specific feature using the information in known covariates and in other features through latent factors, effectively reducing what would be considered MNAR in a univariate context to MAR, conditional on the observed covariates and latent factors. In cases where neither covariates or latent factors contain relevant information, the imputation strategy reduces to a kind of MCAR strategy where the probability of missingness is assumed to be independent of both observed and unobserved data."

2. "In Supplementary Figs. 35 and 36, the colors are difficult to distinguish. The figures should be revised to improve readability."

Our Response: We thank the reviewer for their suggestion. We have adapted these figures to use a conventional color palette as follows:

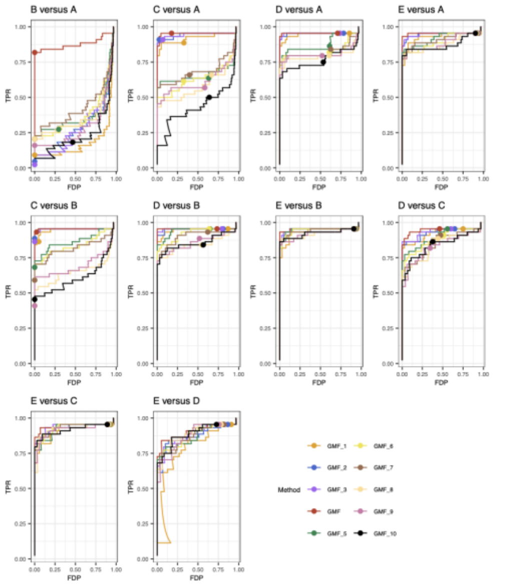

Supp. Fig. 34 Performance evaluation of differential abundance analyses using msqrob2 [7, 8] on the CPTAC dataset [4], for different numbers of latent factors used in omicsGMF. omicsGMF's cross-validation suggested four latent factors. Data from Lab 1, which suffers from ionization issues, are excluded. Human UPS proteins are differentially spiked between conditions while yeast background proteins serve as true negative control. Each curve shows true positive rate (TPR) in function of false discovery proportion (FDP). Dots on each curve represent working points for false discovery rate level at nominal 5% level. Results for all pairwise comparisons between spike-in concentrations are shown. Source data are provided on Zenodo [2].

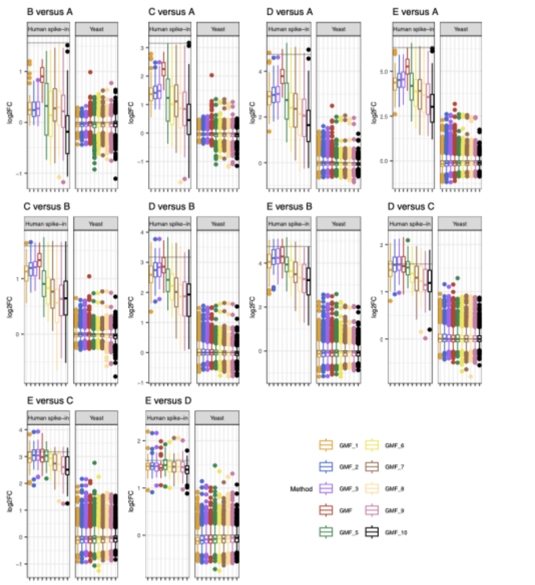

Supp. Fig. 35 Performance evaluation of differential abundance analyses using msqrob2 [7, 8] on the CPTAC dataset [4], for different numbers of latent factors used in omicsGMF. omicsGMF's cross-validation suggested four latent factors. Data from Lab 1, which suffers from ionization issues, are excluded. Human UPS proteins are differentially spiked between conditions while yeast background proteins serve as true negative control. Each plot shows estimated log2 fold changes (FC) by msqrob2 for 44 human spike-in proteins, and 1477 reference yeast proteins. The boxplots are defined by the 25, 50, and 75% percentiles. The whiskers have a maximum length of 1.5 times the interquartile range. Grey line indicates known log2 FC. Source data are provided on Zenodo [2].

Reviewer #3 (Remarks to the Author):

"*I thank the authors for their extensive point-by-point responses and for the inclusion of new quantitative benchmarks, such as the runtime analysis and Average Silhouette Width (ASW). While the technical quality of the work is evident and omicsGMF represents a robust addition to the Bioconductor ecosystem , my primary concerns regarding conceptual novelty and the lack of transformative biological insight remain. While the integration of preprocessing steps is practically useful, the model does not exhibit the level of methodological innovation or novel biological discovery expected for this venue. I have the following comments:*
1. The central claim of the manuscript is the "unified" nature of the omicsGMF framework, which simultaneously addresses batch correction, missing data, and dimensionality reduction. While this integration is practically useful for avoiding the "arbitrary choice of the sequence" in multi-step workflows, the underlying methodological novelty is limited.
(a) Covariate correction and matrix factorization are established practices in other 'omics fields, and omicsGMF largely translates these concepts to proteomics.
(b) The framework reduces to a form of expectation-maximization PCA (EM-PCA) when covariates are omitted, which the authors acknowledge.
(c) The primary innovation—using an efficient adaptive stochastic gradient descent (SGD) algorithm to unlock these models for large datasets—is a significant engineering optimization but represents an incremental statistical advance rather than a breakthrough in computational biology.

2. I appreciate the addition of Supplementary Figures 7 and 8 comparing omicsGMF to sequential workflows. On the Leduc dataset, the authors admit that ASW results are "similar between the most efficient methods" when comparing omicsGMF to workflows that perform batch correction and imputation sequentially. While omicsGMF provides a more user-friendly, single-step approach, the data suggest that a well-constructed traditional pipeline can achieve comparable biological separation.

3. A key requirement for Nature Communications is that a new tool must enable a biological insight that was previously unattainable. The authors demonstrate increased sensitivity in detecting differentially abundant (DA) proteins in spike-in benchmarks like CPTAC and Shen et al. However, these results rely on ground-truth datasets where the biology is already known. The manuscript still lacks a "discovery" component where omicsGMF identifies a novel, actionable biomolecular insight in a complex biological system that existing state-of-the-art tools could not resolve.

4. The authors clarify that their joint modeling approach reduces what would be Missing Not At Random (MNAR) in a univariate context to Missing At Random (MAR) conditional on latent factors. While this is a sensible statistical treatment, it does not fundamentally resolve the biological challenge of proteins missing due to low abundance—a "limit of detection" issue that remains a major hurdle in the field.

The authors have significantly improved the technical depth of the manuscript. However, omicsGMF remains a technical refinement of existing matrix factorization techniques. Given the lack of a primary biological discovery and the incremental nature of the "unified" framework, I maintain my initial assessment that the work does not meet the impact threshold for Nature Communications."

Our response: As Reviewer 3 only expressed their opinion on our manuscript, which appears to be a reiteration of their previous evaluation, without new actionable suggestions, we refer the editors and reviewers to the extensive rebuttal attached to our previous submission in which we elaborated in detail on Reviewer 3's evaluation.

Reviewer #4 (Remarks to the Author):

"I checked the answers to my previous remarks and re-read most of the article. I am overall satisfied and thanks the authors for the in-detail revision, making it easy to follow-up half a year later.

1. Figure1: 'Issues with conventional multi-step workflows..' The figures are showing imputation plus PCA. I would say this is an imputation-only workflow. It was confusing to me

and I only remembered later that you want to address both imputation and batch-correction in one go."

Our response: We thank the reviewer for their suggestions. Based on their remark and the remarks of the editor, we removed this figure from the manuscript, and have adapted the introduction section to motivate our work without showing results. In particular we deleted paragraph 5 of the previous version of the manuscript and adapted the previous paragraph 6 as follows:

~~In Fig. \ref{fig:introduction} and Supplementary Fig. \ref{fig:Introductionfig2}, we illustrate on three example datasets that it is the large number of missing values rather than the biological sources of variability that mainly drive the the PCA upon conventional imputation approaches. Panel B highlights another limitation: PCA does not remove batch effects, which are known to be major sources of variability in LSP and SCP experiments \citep{Vanderaa2021,Vanderaa2023}. Consequently, an additional preprocessing step is required to eliminate batch effects, resulting in lengthy and complex analytical workflows that necessitate proficiency with multiple computational tools.~~

More critically, imputation prior to PCA does not remove batch effects, which are known to be major sources of variability in LSP and SCP experiments \citep{Vanderaa2021,Vanderaa2023}. Consequently, an additional preprocessing step is required to eliminate batch effects, resulting in lengthy and complex analytical workflows that necessitate proficiency with multiple computational tools. Furthermore, the order in which batch correction and imputation are performed also has a substantial impact on downstream analyses \citep{Vanderaa2024}, underscoring the necessity for approaches that perform batch correction and imputation, simultaneously. In this context, scPROTEIN \citep{Li2023} represents an initial attempt to address both batch effects and missing data. However, its batch correction is limited to a single factor, lacking the flexibility to account for multiple technical artifacts and other confounding variables. To ensure robust and accurate proteomics data analysis for LSP and SCP experiments, comprehensive off-the-shelf solutions are needed that can account for the important dataset-specific sources of variation without requiring expert intervention.

2. "*OmicsGMF needs batch-metadata to work (to perform batch correction). It is clear to me that you need these information, but maybe you want to highlight the required inputs, especially as feature-specific variables are not used in the study. I see that the PCA will be an output no-matter what, but this is not the focus of the study in my opinion."*

Our response: We thank the reviewer for their suggestion. We have made this more explicit by adapting the following paragraphs in the Results and Methods sections:

Paragraph 2 of the results now starts as follows: *"omicsGMF estimates all the unobserved parameters* ***U, B****,* Γ *and* ***V****, based on the input sample- and feature-level covariate matrices* ***X*** *and* ***Z****, respectively (note, that these matrices always contain an intercept column)."*

Subsection "omicsGMF Gaussian matrix factorization model" in the Methods now starts as follows:

"Let y_{ij} *be the input intensity of peptide j ($j = 1, ..., J$) in sample i ($i = 1, ..., n$), which we model as a random variable following a Gaussian distribution with mean* μ_{ij}*"*

…

"with ***X*** *a n x p matrix with known sample-level covariates, such as batch or cell type or spike-in concentration, and* ***B*** *a matrix with its corresponding J x p regression parameters,* ***Z*** *a J x q matrix with known feature specific covariates, and* Γ *a matrix with its corresponding n x q regression parameters. Both* ***X*** *and* ***Z*** *are input matrices, and by default contain a column of ones, corresponding to gene-specific and sample-specific intercepts, respectively."*
